# Unveiling RCOR1 as a rheostat at transcriptionally permissive chromatin

**DOI:** 10.1038/s41467-022-29261-0

**Published:** 2022-03-23

**Authors:** Carlos Rivera, Hun-Goo Lee, Anna Lappala, Danni Wang, Verónica Noches, Montserrat Olivares-Costa, Marcela Sjöberg-Herrera, Jeannie T. Lee, María Estela Andrés

**Affiliations:** 1 Department of Cellular and Molecular Biology, Faculty of Biological Sciences, Pontificia Universidad Católica de Chile, Santiago, 8331150 Chile; 2 Department of Molecular Biology, Massachusetts General Hospital, Boston, MA 02114 USA; 3 Department of Genetics, The Blavatnik Institute, Harvard Medical School, Boston, MA 02114 USA

**Keywords:** Chromatin, Transcriptional regulatory elements

## Abstract

RCOR1 is a known transcription repressor that recruits and positions LSD1 and HDAC1/2 on chromatin to erase histone methylation and acetylation. However, there is currently an incomplete understanding of RCOR1’s range of localization and function. Here, we probe RCOR1’s distribution on a genome-wide scale and unexpectedly find that RCOR1 is predominantly associated with transcriptionally active genes. Biochemical analysis reveals that RCOR1 associates with RNA Polymerase II (POL-II) during transcription and deacetylates its carboxy-terminal domain (CTD) at lysine 7. We provide evidence that this non-canonical RCOR1 activity is linked to dampening of POL-II productive elongation at actively transcribing genes. Thus, RCOR1 represses transcription in two ways—first, via a canonical mechanism by erasing transcriptionally permissive histone modifications through associating with HDACs and, second, via a non-canonical mechanism that deacetylates RNA POL-II’s CTD to inhibit productive elongation. We conclude that RCOR1 is a transcription rheostat.

## Abstract (web summary)

The classical neuronal-gene corepressor RCOR1/CoREST is paradoxically enriched in transcriptionally active chromatin. Here the authors show RCOR1 is recruited during promoter-proximal pausing and negatively regulates the nascent-transcript synthesis. They also show that an RCOR1-LSD1- HDAC1 complex removes lysine acetylation from RNA polymerase II to repress transcription.

## Introduction

The state of eukaryotic gene expression is determined in part by a combination of transcription factors and chromatin-modifying enzymes^1,2^, which together establish and maintain specific chromatin landscapes^3–6^. Chromatin domains are associated with various transcriptional activities in accordance with relative enrichment of specific histone modifications^7,8^. For example, di and tri-methylation of histone H3 lysine-4 (H3K4me2/3) are linked to transcriptionally permissive chromatin, whereas tri-methylation of histone H3 lysine-9 and −27 (H3K9me3 and H3K27me3) are linked to constitutive and facultative heterochromatin domains^3,6,7^. The REST Corepressor 1 (RCOR1/CoREST1) was one of the first characterized co-repressor proteins based on its ability to induce transcriptional silencing of neuronal genes when interacting with RE1-Silencing Transcription Factor (REST)^9,10^. Among the three reported members of the RCOR family of proteins in mammals, RCOR1 has the highest repressive capacity over target reporter genes in vivo^11^. It forms a core co-repressor complex by associating with lysine-specific demethylase 1A (LSD1, KDM1A)^12^ and Class I histone deacetylases 1 and 2 (HDAC1/2)^10,13,14^. Therefore, in relation to the histone code hypothesis^7,15,16^, the LSD1-RCOR1-HDAC1/2 complex may be understood as a biochemical entity that represses transcription by erasing transcriptionally permissive histone modifications, such as mono- and di-methylation of histone H3 lysine 4 (H3K4me1/2) and histone acetylation^9,10,12–14^. RCOR1 is the only RCOR family member that efficiently stimulates LSD1 and HDAC1 activities on nucleosomal substrates^17–20^.

RCOR1 structure includes one ELM2 (Egl-27 and MTA1 homology 2) domain and two SANT (Swi3, Ada2, N-Cor, and TFIIIB) domains. The first SANT domain localizes contiguously to the ELM2 domain, and both are required for RCOR1 interacting with HDAC1/2. Structural evidence has shown that the SANT2 domain establishes contacts between the RCOR1-LSD1 complex and nucleosomes^20–23^, suggesting it can interact with chromatin without a bona fide transcription factor. Interestingly, since RCOR1-LSD1 complex interaction either with naked DNA or with nucleosomes is impaired at high concentrations of monovalent ions and limited lengths of internucleosomal DNA^21,23^, it has been suggested that electrostatic interactions and accessible nucleosome conformations could favor the recruitment of the complex to chromatin. Furthermore, a recent report described the first structural evidence for the ternary LSD1-RCOR1-HDAC1/2 complex, which shows a bilobed structure in which only one of the enzymes can interact with the substrate at a given time^24^, suggesting that the complex works in a coordinated way to erase transcriptionally permissive marks from histones. This finding is consistent with classical biochemical observations where the LSD1 activity is weaker when HDACs are pharmacologically inhibited^18,19^.

Although some transcription factors such as REST, Gfi, and Nurr1 have been proposed as RCOR1 recruiters^9,25,26^, structural data show that the LSD1-RCOR1-HDAC1/2 complex interacts directly with nucleosome components and extranucleosomal DNA^20–24^, suggesting other ways for its function in chromatin. Although the substrate of this complex is present in active chromatin, it is still an unsolved hypothesis if the role of this complex is mostly exerted for the maintenance of heterochromatin compartments or it is instead actively removing transcriptionally permissive marks in euchromatin. Here, we take a multidisciplinary approach to investigate RCOR1 dynamics and uncover an unconventional mechanism of gene repression in which RCOR1 preferentially localizes to actively expressed genes. We find that, surprisingly, RCOR1 not only erases transcriptionally permissive histone modifications through associating with HDACs, but also deacetylates RNA POL-II’s CTD at lysine 7. We provide evidence that this non-canonical activity is linked to regulation of POL-II transcription pause-release and/or elongation.

## Results

### Different RCOR1-containing complexes segregate inside cells and are mostly enriched in interchromatin space

**Fig. 1:**
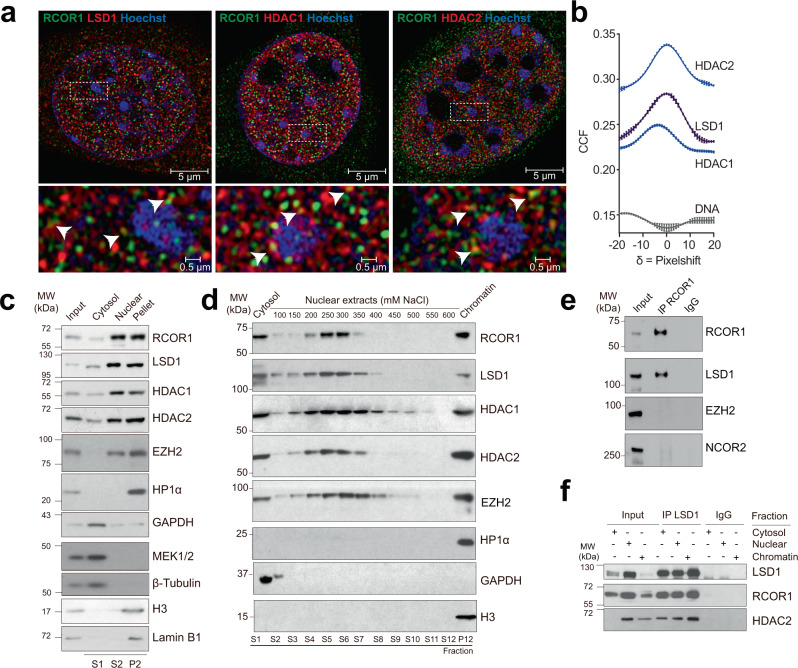
Three different subpopulations of the LSD1-RCOR1-HDAC1/2 complex coexist in cells. **a** HT22 cells were stained with double immunolabeling of RCOR1 (green) and LSD1, HDAC1, or HDAC2 as shown in red. Bottom panels show magnified regions of original images highlighted inside dashed rectangles. White arrows indicate regions where RCOR1 and its binding partners are colocalizing. Images are representative of three independent experiments. **b** Van Steensel’s plot of 2D colocalization between RCOR1 and each co-stained protein or DNA. CCF: Cross correlation function. **c** Western blot analysis of subcellular fractionation in HT22 cells. GAPDH, MEK1/2, and β-Tubulin were assayed as cytosolic markers, while H3 and Lamin B1 as chromatin and nuclear lamina markers, respectively. S: Supernatant. P: Pellet. MW: Molecular weight. kDa: Kilodalton. Panels are representative of three independent experiments. **d** Western blot analysis of sequential salt extractions of nuclear contents in HT22 cells. GAPDH and H3 were assayed as cytosolic and chromatin markers, respectively. Images are representative of two independent experiments. **e** Western blot analysis of the immunoprecipitation of RCOR1. LSD1 was assayed as a positive control of co-immunoprecipitation. IP: Immunoprecipitation. IgG: Immunoglobulin. This experiment was performed once. **f** Western blot analysis of the immunoprecipitation of LSD1 in cytosolic, nuclear soluble, and chromatin fractions. IP: Immunoprecipitation. IgG: Immunoglobulin. Panels are representative of two independent experiments. Source data are provided as a Source Data file.

Based on reports showing the RCOR1 complex as the strongest co-repressor of its family^11^, we aimed to explore its subnuclear distribution. We analyzed the subcellular distribution of RCOR1, LSD1, and HDAC1/2 in undifferentiated HT22 where the ubiquitous LSD1 variant is predominant (Supplementary Fig. 1a). Immunostainings to double-label RCOR1 and each subunit of the complex were analyzed at high-confocal resolution using Airyscan acquisition and super-resolution processing. We observed that the subunits of the complex were enriched inside the nucleus, although some cytoplasmic localization was detected (Fig. 1a). As expected, we found RCOR1 establishing contacts or localizing in close proximity to its partners LSD1, HDAC1, and HDAC2 (Fig. 1a). These observations were confirmed by finding a significant and positive correlation of fluorescent signals for each pair of proteins (Fig. 1b). This correlation analysis reached a maximum value of Pearson’s coefficient at δ = 0, indicating a significant correlation between the two fluorophores that is not due to random overlapping of fluorophore signals. We highlight that the correlation between RCOR1 and DNA reached a minimal value at δ = 0, indicating a negative global colocalization with dense DNA regions stained by Hoechst (Fig. 1b and Supplementary Figs. 1b, c), which are mostly enriched in heterochromatin domains^27,28^. These findings indicate that, while RCOR1 is concentrated in the nucleus, it is mostly excluded from heterochromatin domains, suggesting that RCOR1 localizes at euchromatin domains and/or nucleoplasmic space. To examine this intriguing possibility, we performed biochemical fractionations on HT22 cells and obtained a cytosolic fraction (enriched in the markers GAPDH, MEK1/2, and β-Tubulin), a DNA-free soluble nuclear fraction, and a pellet enriched in both chromatin and nuclear lamina components such as histone H3 and Lamin B1 (Fig. 1c and Supplementary Fig. 1d). Under these conditions, we found that RCOR1, LSD1, and HDAC1/2 showed a similar distribution pattern and were enriched in the nuclear fractions (Fig. 1c).

Given that we detected all the subunits of the LSD1-RCOR1-HDAC1/2 complex in the three cell fractions, we wondered if they correspond to different subcellular populations. To this end, we performed sequential nuclear extractions using increasing concentrations of NaCl, and analyzed the distribution of proteins among the different fractions. We observed that RCOR1, LSD1, and HDAC1/2 were distributed in three different subcellular populations: a cytosolic one, a nuclear soluble that was efficiently extracted between 250–300 mM NaCl, and an additional population that resisted all the salt-induced extractions and remained enriched in chromatin (Fig. 1d). We found that the EZH2 was distributed in two subnuclear populations, one that was efficiently extracted at higher ionic strength than RCOR1 (300–350 mM NaCl) and another one that remained on chromatin like RCOR1. Since EZH2 and RCOR1 were co-extracted in similar fractions, we performed RCOR1 immunoprecipitation and found that EZH2 was not present as part of the RCOR1 immunocomplex (Fig. 1e). On the other hand, HP1α resisted all the extractions, reflecting its strong binding to chromatin, as we could only detect it on the final chromatin pellet (Fig. 1d). Thus, we confirmed that the presence of RCOR1, LSD1, and HDAC1/2 in the different subcellular fractions evidenced three subpopulations of the complex subunits. Furthermore, given the lack of interaction or higher extractability shown by the RCOR1-related soluble nuclear species compared to factors that are classical markers of heterochromatin, it suggests that RCOR1 complexes might be located at different chromatin and/or nucleoplasmic domains than EZH2, NCOR2, and HP1α.

To check if RCOR1 forms complexes with LSD1 and HDAC1/2 in the three different subcellular populations, we performed LSD1 immunoprecipitation on cytosolic, nuclear soluble, and chromatin soluble fractions. As expected, we found that LSD1 co-precipitated RCOR1 and HDAC2 in the three analyzed fractions (Fig. 1f), confirming the three detected RCOR1-subpopulations form complexes inside subcellular environments with and without chromatin. Altogether, these data show that RCOR1 complexes are distributed in different cell compartments and its association with chromatin is weaker than canonical repressive complexes.

### RCOR1 is mostly enriched at transcriptionally permissive chromatin

**Fig. 2:**
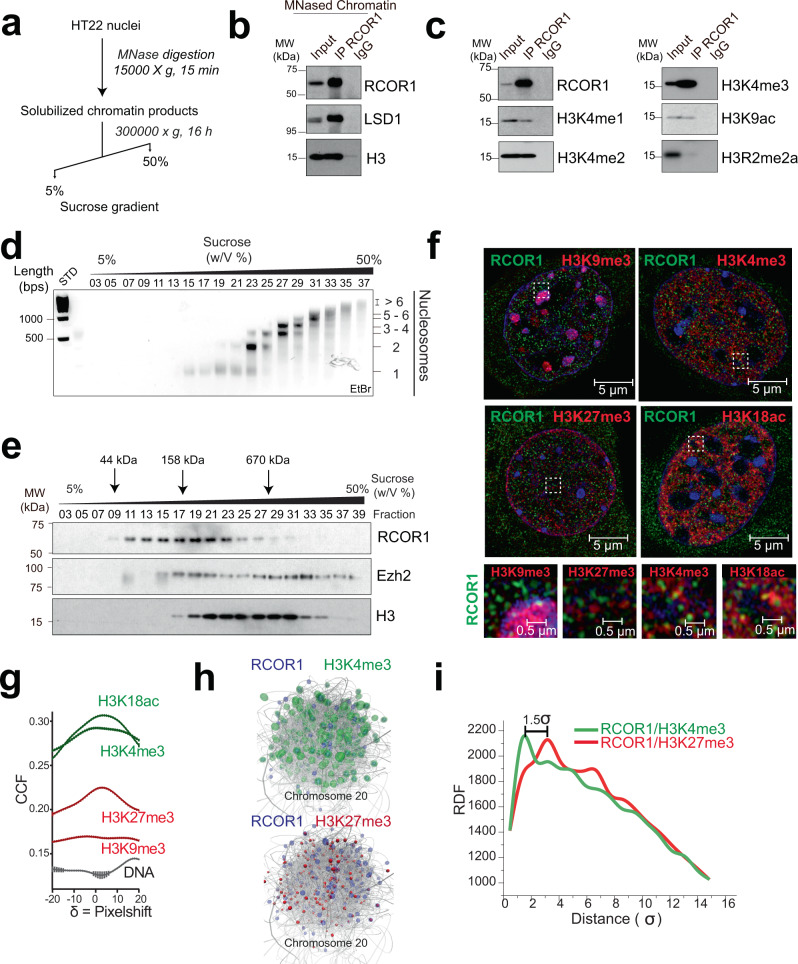
RCOR1 is enriched in accessible, transcriptionally permissive chromatin. **a** Scheme depicting the MNase digestion of chromatin coupled to ultracentrifugation for chromatin accessibility assays. **b** Western blot analysis of the immunoprecipitation of RCOR1 on MNase-treated chromatin. Panels are representative of two independent experiments. **c** Western blot analysis of RCOR1 coimmunoprecipitated histone H3 modifications from MNased chromatin extracts. Panels are representative of two independent experiments. **d** Agarose gel showing the distribution of DNA fragments among the different fractions obtained from the ultra-centrifuged chromatin products. Bps: base pairs. STD: DNA Ladder. EtBr: Ethidium Bromide. This experiment performed once. **e** Western blot analyses of distribution of RCOR1, EZH2 and H3 in the sucrose gradient sedimentation equilibria. Upper arrows indicate standard molecular sizes resolved by this method. This experiment was performed once. **f** HT22 cells were stained with double immunolabeling of RCOR1 (green) and different histone modifications as shown in red. Right panels show magnified regions of original images highlighted inside dashed squares. Images are representative of three independent experiments. **g** Van Steensel’s plot of 2D colocalization between RCOR1 and each co-stained histone modification or DNA. CCF: Cross correlation function. **h** RCOR1 (blue) and H3K4me3 (green) or H3K27me3 (red) ChIP-seq positions mapped in 3D simulated chromosome 20 of human K562 cells based on Hi-C information as constraints. **i** Radial distribution function analysis of the 3D colocalization between RCOR1 and histone modifications. RDF: Radial distribution function. Source data are provided as a Source Data file.

Previous results prompted us to characterize the properties of RCOR1 interaction with nucleosomes in the context of chromatin. To this end, we tested whether RCOR1 nuclear populations display detectable interactions with nucleosomes. We performed MNase treatments on HT22 nuclei (Fig. 2a), and soluble products (Supplementary Fig. 2a) from the digestion – containing between 1 and >6 nucleosomes – were cleared and subjected to RCOR1 immunoprecipitation. We found that interaction with histone H3 was noticeable (Fig. 2b), confirming that RCOR1 forms stable complexes with nucleosomes in chromatin. We also analyzed the histone modifications that co-precipitate with RCOR1. We detected interactions with transcriptionally permissive modifications such as H3K4me1, H3K4me2, H3K4me3, and H3K9ac, but not with H3R2me2a (Fig. 2c), supporting that nuclear RCOR1 interacts with euchromatin. Next, we tested whether RCOR1 is enriched at accessible chromatin. Therefore, we scaled up the MNase digestion procedure, and the solubilized products were loaded on a 5 to 50% sucrose gradient and then subjected to ultracentrifugation. The gradient showed an efficient separation of nucleosome-free nuclear fractions (Fig. 2d, Fractions 03–13), mononucleosomes (Fraction 19), dinucleosomes (Fraction 23), and oligonucleosomes between 3 and 6 nucleosome units (Fractions 27–35). Under these conditions, most of the non-histone proteins from the loaded nuclear material were separated in nucleosome-free fractions while histones were consistently distributed among the fractions enriched in nucleosomal DNA (Supplementary Fig. 2b).

RCOR1 was distributed at fractions between 70 and 350 kDa (Fig. 2e), indicating the co-existence of monomers and distinct RCOR1-containing complexes. A considerable amount of RCOR1 sedimented at fractions containing mono and dinucleosomes (Fig. 2d, Fractions 17–23), suggesting that RCOR1 is mostly enriched at MNase-accessible chromatin. To confirm this, we compared its sedimentation equilibrium with EZH2, which besides showing a peak at accessible chromatin (Fraction 19), also showed a second peak at denser fractions than RCOR1 (Fraction 33). Thus, RCOR1 is mostly enriched at chromatin domains more accessible than the heterochromatin marker EZH2.

To further validate our results, we performed immunofluorescence assays to visualize if RCOR1 is localized closer to transcriptionally permissive chromatin. Double staining RCOR1 with H3K18ac and H3K4me3 as euchromatin markers, or with H3K9me3 and H3K27me3 as heterochromatin markers showed that RCOR1 is closer and establishes more frequent contacts with H3K18ac and H3K4me3 than with H3K9me3 and H3K27me3 (Fig. 2f). Quantitative analyses of colocalization revealed a higher partial colocalization of RCOR1 with transcriptionally permissive histone modifications than with repressive ones (Fig. 2g).

To have a global view of this finding, we created a high-resolution 3D model of chromosome 20 of human K562 cells. We conducted Monte Carlo simulations by representing the 63 Mb of chromosome 20 as a polymer made of 12,592 beads spanning 5 Kb each. The simulation folded chromosome 20 (Supplementary Fig. 2c) guided by 3D contacts as constraints obtained from public Hi-C datasets. Localization of H3K4me3 and H3K27me3 ChIP-seq peaks derived from ENCODE datasets on K562 cells was assessed to distinguish transcriptionally active and repressed compartments (Supplementary Fig. 2d). We compared the 3D distribution of RCOR1 ChIP-seq peaks to these histone modifications, and observed RCOR1 colocalizing more frequently with H3K4me3 rather than H3K27me3 (Fig. 2h, Supplementary Fig. 2e). This observation was significantly distinguishable, as radial distribution functions of RCOR1 and each histone modification showed RCOR1 closer to H3K4me3 rather than H3K27me3 (Fig. 2i). We conclude that RCOR1 is primarily found in accessible and actively transcribed chromatin.

### The LSD1-RCOR1-HDAC1 complex marks proximal promoters in euchromatin

**Fig. 3:**
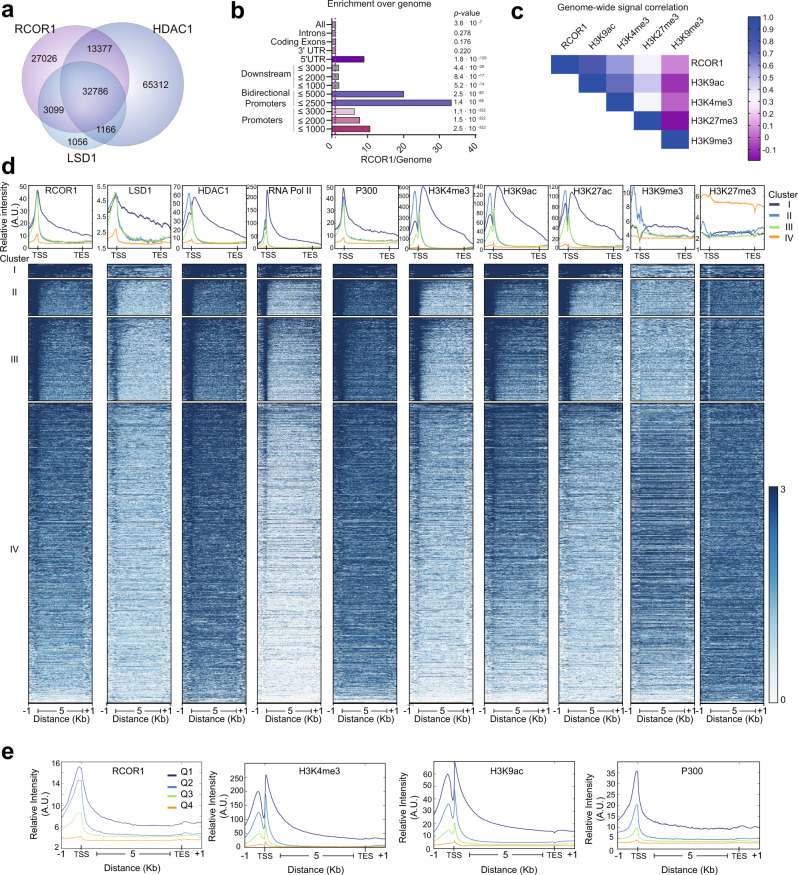
RCOR1 is enriched in proximal promoters of active genes. **a** Venn diagram showing the number of ChIP-seq peaks of RCOR1, LSD1, and HDAC1 on K562 cells. **b** Enrichment of ChIP-seq peaks on DNA elements over the natural abundance of these elements across the genome. Dashed violet line shows enrichment over genome abundance. P values are shown next to each bar. **c** Genome-wide signal correlation heatmap showing the Spearman correlation coefficient between genome-wide signals of RCOR1 and different histone modifications. **d** Meta-gene profiling of RCOR1, LSD1, HDAC1, RNA POL-II, P300, and histone modifications in K562 cells. Upper plots show the normalized signal profiles for the occupancies of each protein or modification analyzed in different RCOR1 clusters. Lower plots show the signal heatmap from the genes in the four clusters. **e** Quartile-based comparison of relative occupancies of RCOR1, H3K4me3, H3K9ac, and P300 on RCOR1-sorted gene quartile groups.

The provided microscopical and biochemical evidence suggested a preferential association between RCOR1 and euchromatin. This was mostly unexpected, given that RCOR1 complexes have primarily been associated with transcriptional repression^29^. We further performed bioinformatic analyses of available RCOR1, LSD1, and HDAC1 ChIP-seq datasets from the ENCODE project on human K562 cells. Our analysis showed that 35,885 out of 38,117 LSD1 peaks (94.1%) are co-occupied with RCOR1 (Fig. 3a). In addition, 46,163 out of 112,641 HDAC1 peaks (41.0%) are co-occupied with RCOR1. Notably, 32,786 out of 47,329 LSD1/RCOR1 or HDAC1/RCOR1 shared peaks were co-occupied by the three subunits of the complex, highlighting the significant co-occupancy of the core RCOR1 complex components at the genome-wide scale. Next, we explored the genomic elements where RCOR1 binding was enriched. We tested it in different genomic features over genomic background and found that RCOR1 peaks were significantly over-represented at promoters and 5’ UTR (Fig. 3b and Supplementary Fig. 3a). Interestingly, bidirectional promoters were also enriched. Similar results were observed in mouse CH12 cells (Supplementary Fig. 3b).

In order to determine the chromatin features where RCOR1 is enriched, we conducted a peak-centric meta-analysis and found that RCOR1 binding sites were enriched with H3K27ac with strong correlation but not with H3K27me3 (Supplementary Fig. 3c). In addition, we analyzed unbiased global correlation between the signal distributions of RCOR1 and various histone marks by calculating Spearman correlation in 10 kb bins throughout the genome and observed a higher correlation between RCOR1 and euchromatin marks such as H3K9ac and H3K4me3 than with the repressive marks H3K9me3 and H3K27me3 at the genome-wide scale (Fig. 3c), supporting our previous experiments. We next decided to look at the RCOR1 distribution in a gene-centric way, generating 4 clusters of RCOR1-targeted genes by the k-means clustering approach (Fig. 3d and Supplementary Fig. 3d). Cluster I and II showed a subset of genes with the highest RCOR1 enrichment, but depicting a peak of H3K4me3 downstream or upstream of their TSS, respectively. Cluster III showed weaker binding of RCOR1 and weaker signals of active histone marks and cluster IV was mostly depleted of RCOR1 enrichment at the TSS. In conditions where the genomic distributions of LSD1 and HDAC1, as bonafide RCOR1 interactors, were similar, we observed a positive correlation between RCOR1 and markers of transcriptionally permissive chromatin such as RNA Polymerase II (RNA POL-II), the acetyltransferase P300, H3K4me3, H3K9ac, and H3K27ac. In addition, we detected a negative or no correlation between RCOR1 peaks and markers of heterochromatin, such as H3K27me3 and H3K9me3, suggesting that RCOR1 is preferentially enriched at euchromatin domains. To confirm these findings, we regrouped genes into quartiles (Q) according to the RCOR1 binding level and verified that occupancy of RCOR1 correlated with high occupancy of H3K4me3, H3K9ac, and P300 (Fig. 3e). We also analyzed the co-occupancy of RCOR1 with other co-repressors SIN3A and NurD (CHD4) complexes and found that certain regions are co-occupied by multiple co-repressors but the majority of RCOR1 peaks were distinct from the other co-repressors (Supplementary Fig. 3e). In line with this, RCOR1 binding showed little or no correlation with the binding signals of two PRC2 components EZH2 and SUZ12 (Supplementary Fig. 3f, g).

Intriguingly, the clustering analysis revealed two different patterns of RCOR1 binding. While clusters I, II, and III showed a clear enrichment of RCOR1 around the TSS, the cluster I, which contained 849 target-genes, also showed an occupancy significantly higher on gene body regions (Fig. 3d). A representative example of this cluster is the FKBP2 gene, where RCOR1 is distributed at its TSS as well as downstream on its gene body, along with RNA POL-II and other active-transcription markers (Supplementary Fig. 4a). On the other hand, we observed that genes with bidirectional promoters^30,31^ were overrepresented in cluster II, suggesting a role for RCOR1 on the regulation of this kind of genes, as exemplified by the SNX5/MGME1 promoter (Supplementary Fig. 4b). When we analyzed the distance between the TSS of the genes on each cluster and their nearest bidirectional transcripts, we found that the median distance measured for cluster II was two orders of magnitude lower than the other clusters, underlining RCOR1-regulated genes in cluster II are located very near to their neighboring divergent genes (Supplementary Fig. 4c). Altogether, these data show that LSD1-RCOR1-HDAC1 complex preferentially binds TSS regions, gene bodies, and bidirectional promoters in euchromatin.

### RCOR1 is preferentially enriched in highly expressed genes

**Fig. 4:**
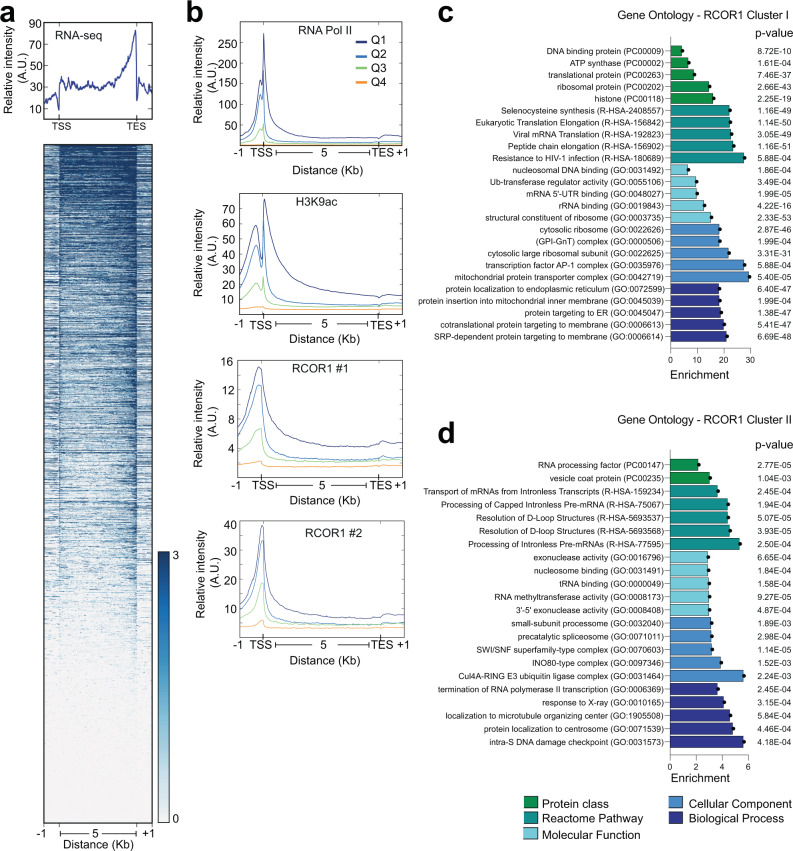
RCOR1 occupancies are positively correlated with gene expression. **a** Meta-gene plots of RNA-seq data in K562 cells. Lower diagram shows the heatmap of the signal intensity from RNA-seq data sorted by intensity. **b** Quartile-based comparison of relative RNA POL-II, H3K9ac, and RCOR1 occupancies on the genes in different quartile groups based on RNA-seq signal intensity. Two different RCOR1 datasets (using different antibodies) were analyzed (labeled as #1 and #2). **c** and **d** Gene ontology analysis of RCOR1 clusters I and II, respectively. Bar colors represent different ontology categories, highlighted at the bottom.

The positive correlation between RCOR1 and markers of actively transcribed chromatin prompted us to examine whether RCOR1 peaks were enriched in genes that are actually being transcribed. For this purpose, we performed a cross-examination between RCOR1 ChIP-seq and RNA-seq data from ENCODE datasets. Genes were sorted according to their transcription levels from RNA-seq data (Fig. 4a) and grouped into four quartiles, and as expected, they positively correlated to euchromatin markers such as RNA POL-II and H3K9ac (Fig. 4b). Importantly, the highly expressed genes (Quartiles Q1 and Q2) were more enriched in RCOR1 than the less-expressed ones (Quartiles Q3 and Q4) (Fig. 4b). To inquire about the functions related to RCOR1-regulated genes, we performed a Gene Ontology (GO) analysis for RCOR1 clusters I and II (Fig. 4c, d), which revealed significant enrichment in actively expressed genes such as histones, ATP synthase, translation-related proteins, and others. Altogether, bioinformatics analyses suggest that RCOR1 is preferentially positioned in proximal promoters of highly expressed genes in euchromatin.

### RCOR1 interacts with RNA POL-II after assembly of the pre-initiation complex

**Fig. 5:**
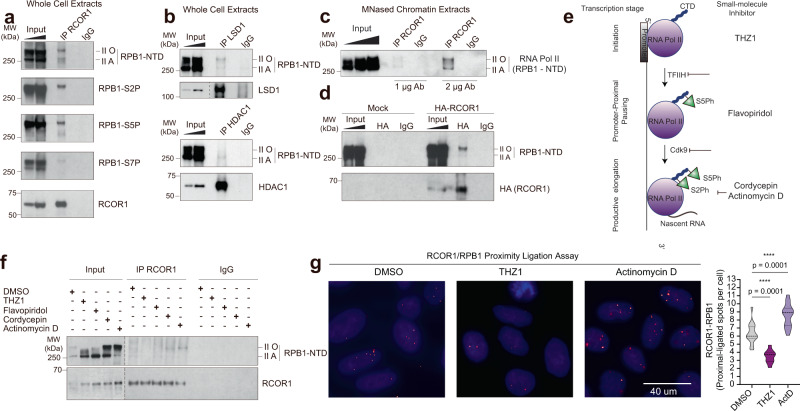
RCOR1 establishes an interaction with RNA POL-II after initiation and before elongation. **a** Western blot analyses of immunoprecipitation of RCOR1 and co-precipitation of RPB1 and its phosphorylated isoforms from whole cell extracts. II-O: Hyperphosphorylated isoforms. II-A: Hypophosphorylated isoforms. RPB1-NTD CoIP panel is representative of four independent experiments while others are representative of two independent experiments. **b** Western blot analyses of immunoprecipitation of LSD1 (top) or HDAC1 (bottom) and co-precipitation of RPB1. Experiment performed once. **c** Western blot analysis of immunoprecipitation of RCOR1 and RNA POL-II from MNased chromatin using 2 different RCOR1 antibody concentrations. Panels are representative of two independent experiments. **d** HA-Pulldown on extracts derived from empty-vector and HA-RCOR1 overexpressing HEK293 cells. Panels are representative of two independent experiments. **e** Scheme depicting different stages of eukaryotic RNA POL-II transcription. It highlights the steps that are inhibited by THZ1, Flavopiridol, Cordycepin, and Actinomycin D. CTD: Carboxy-terminal domain. S5Ph: Phosphorylated serine 5. S2Ph: Phosphorylated serine 2. **f** Western blot analyses of RCOR1 immunoprecipitation and RPB1 co-precipitation under inhibition of transcription at different steps. Panels are representative of three independent experiments. **g** Proximity ligation assay between RCOR1 and RPB1 under DMSO, THZ1 and Actinomycin D treatments in HT22 cells. Right graph shows the distribution of proximal-ligated spots per cell under each treatment. More than 128 cells from three independent experiments were analyzed per treatment. Statistical significance was checked on Graphpad Prism by confirming normal distribution of data by D’Agostino & Pearson, Shapiro-Wilk and KS normality tests. Data was subjected to an ordinary, unpaired, one-way ANOVA test with 99% confidence intervals, **** means *p* < 0.01. Source data are provided as a Source Data file.

RCOR1’s association with highly expressed genes is counterintuitive but is reminiscent of PRC2’s association with active genes^32^. We asked if there could be a functional relationship to RNA POL-II—specifically with the largest subunit, RNA Polymerase Subunit B1 (RPB1)^33^. Notably, RPB1 has a C-terminal domain (CTD) heptapeptide tandem YSPTSPS repeat that is actively phosphorylated at different stages of the transcription cycle^34,35^. RCOR1 immunoprecipitation experiments from native HT22 extracts showed that both the hypo (II A) and hyper (II O) phosphorylated RPB1 isoforms co-precipitated with it (Fig. 5a). Independent immunoprecipitations showed complexes between RCOR1 and specific RPB1 phosphorylations at serine 2, 5, and 7 on its C-terminal domain (Fig. 5a). These observations demonstrate that RCOR1 interacts specifically with the active POL-II holoenzyme. In line with this, we also detected RPB1 as an LSD1 and HDAC1 interactor (Fig. 5b). In addition, RCOR1-RPB1 interaction was detectable on solubilized, MNase-treated chromatin (Fig. 5c). To confirm this RCOR1 interaction, we transiently overexpressed an HA conjugated N-terminal tagged RCOR1 construct on HEK293T cells. HA-pulldown experiments demonstrated that RPB1 was co-precipitated only when HA-RCOR1 was transfected (Fig. 5d), confirming the specificity of this interaction. High-resolution confocal microscopy showed that RCOR1 partially colocalized with RPB1 both in mouse (HT22) and human (HeLa) cells (Supplementary Fig. 5a, b).

Finally, to further determine at which stage of the transcription cycle RCOR1 is recruited to the transcriptional machinery, we treated HT22 cells with THZ1, Flavopiridol, and Cordycepin or Actinomycin D for 1 h, in order to inhibit transcription before initiation, at promoter pausing, before productive elongation, or at elongation, respectively^36^ (Fig. 5e). These treatments caused global variations on the phosphorylation degree of RPB1, consistent with THZ1 and Flavopiridol upregulating hypo-phosphorylated variants and Cordycepin and Actinomycin D causing hyper-phosphorylated states^36^. We observed that THZ1 treatment decreased RCOR1 interaction with RPB1 (Fig. 5f and Supplementary Fig. 5c), suggesting that RCOR1 engagement of POL-II depends on formation of the pre-initiation complex (PIC). On the other hand, treating with Flavopiridol, Cordycepin, and Actinomycin D did not affect RCOR1’s interaction with POL-II (Fig. 5f), suggesting that RCOR1 must load onto the POL-II complex prior to pausing and productive elongation. Complementary proximity ligation assays (PLA) between RCOR1 and RPB1 confirmed that colocalization was significantly and specifically reduced under THZ1 treatment but augmented in presence of actinomycin D (Fig. 5g and Supplementary 5d, e). Thus, RCOR1 associates with POL-II only after the PIC is formed.

Based on these results, we hypothesized that RCOR1’s localization on chromatin would be sensitive to transcriptional perturbations. To test this idea, we performed a sequential salt-extraction of nuclear proteins in THZ1 versus Actinomycin D-treated cells. Whereas THZ1 caused enrichment of hypo-phosphorylated RPB1 in the cytosol, it had no effect on RPB1 levels nor RCOR1’s subcellular distribution (Supplementary Fig. 5f, g), consistent with THZ1 blocking recruitment of RCOR1 to POL-II during PIC formation. On the other hand, Actinomycin D treatment “trapped” hyperphosphorylated POL-II in chromatin and this in turn caused RCOR1 to be similarly trapped in the chromatin fraction, as RCOR1 needed higher salt concentrations for extraction in Actinomycin D-treated cells relative to DMSO-treated cells. Altogether, these data suggest that RCOR1 interacts with RNA POL-II after formation of the PIC at the stage of promoter-proximal pausing. Our data indicate that, if RCOR1 regulates transcription directly, it must do so at the step of pausing, pause-release, or elongation.

### RCOR1 dampens transcription at active genes

**Fig. 6:**
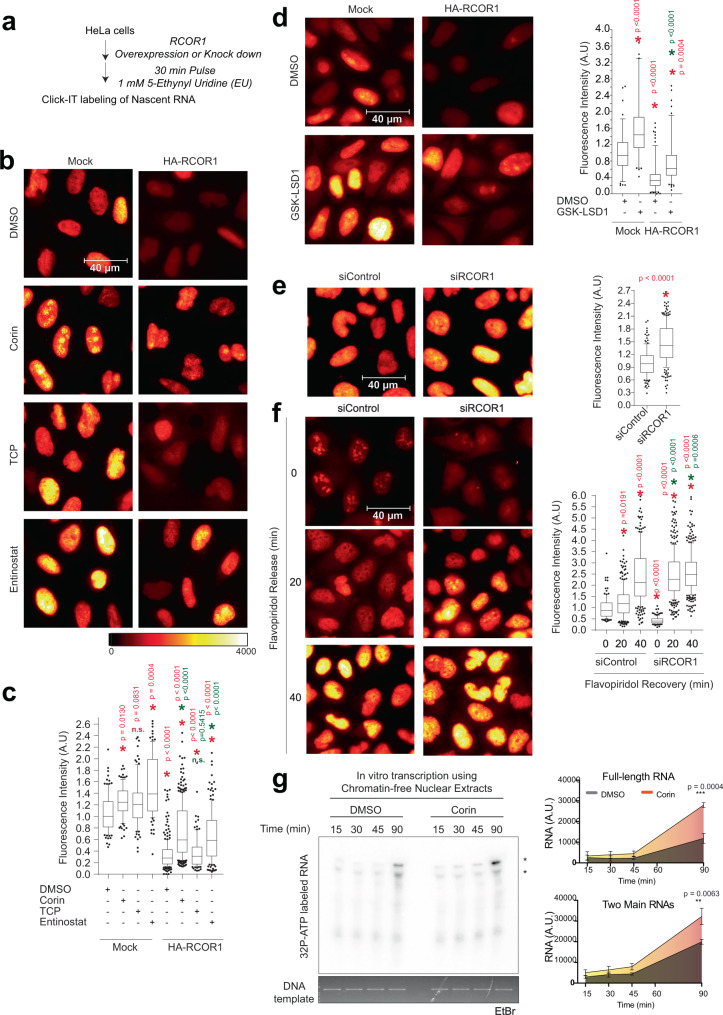
RCOR1 globally represses transcription of newly synthesized transcripts. **a** Workflow depicting the followed steps for imaging of newly synthesized RNAs under RCOR1 overexpression or knock down in HeLa cells. **b** Pseudocolored images of labeled transcripts under HA-RCOR1 overexpression. Scale bar represents 40 µm. **c** Box plots showing the quantitation of the relative fluorescent intensities per cell on each condition. Red asterisks indicate *p* < 0.05 respect to Mock-DMSO control. Green asterisks indicate *p* < 0.05 as significant rescue of the decreased transcription produced by RCOR1 overexpression. Non parametric, unpaired Kruskal Wallis tests were performed to evaluate statistical significance. **d** Pseudocolored images of labeled transcripts under HA-RCOR1 overexpression and DMSO or GSK-LSD1 treatments. Scale bar represents 40 µm. Right box plot shows the quantitation of the relative fluorescent intensities per cell on each condition. Asterisks indicate statistical significance as in (**c**). **e** Pseudocolored images of labeled transcripts under RCOR1 KD. Scale bar represents 40 µm. Right box plot shows quantitation of the relative fluorescent intensities per cell under RCOR1 KD conditions. Red asterisk indicates *p* < 0.05 respect to siControl according to non parametric, two tailed Mann Whitney test. **f** Pseudocolored images of labeled nascent transcripts when RCOR1 KD cells were subjected to recovery after washing out flavopiridol at 0, 20 and 40 min. Scale bar represents 40 µm. Box plots to the right show the quantitation of the relative fluorescent intensities per cell after each time point. Red asterisks represent *p* < 0.05 with respect to siControl cells at time 0 min according to non parametric, unpaired Kruskal Wallis tests were performed to evaluate statistical significance. Green asterisks represent *p* < 0.05 significantly different nascent transcription between the two groups at 20 min according to non parametric, two tailed Mann–Whitney test. **g** In vitro transcription using chromatin-free nuclear extracts from HeLa cells treated with Corin. Transcripts were labeled with 32P-α-ATP and the DNA template used was stained with EtBr as a parallel experiment. Right plots show the quantitation of the main or both transcript(s) obtained in this assay at each time point. *** indicates *p* < 0.001 whereas ** indicates *p* < 0.05 significantly different activity of Corin versus DMSO treated extracts (*n* = 3). *Additional representative images of EU-incorporation experiments are available in Supplementary Fig. 7. Statistical tests used for Fig. 6c–f were two-sided, unpaired *T*-tests.

The association with active genes remains curious. Does RCOR1 function as a co-activator or a co-repressor at actively expressed genes? To address this question, we modulated the steady-state RCOR1 protein levels by transient overexpression (Fig. 6a and Supplementary Fig. 6a), or by post-transcriptional silencing with siRNA in HeLa cells (Fig. 6a and Supplementary Fig. 6b). Twenty-four hours after transfection, we incubated cells with a 30-min pulse of 1 mM 5 Ethynyl Uridine (EU), which is incorporated into nascent RNA molecules and that in the presence of divalent copper ions can then be tagged by a chemical reaction with fluorophore-conjugated azide groups^37^. Therefore, we were able to label and visualize transcripts that were synthesized for 30 min in cells. When RCOR1 was overexpressed, we detected a decrease in the fluorescence intensity of nascent transcripts (Fig. 6b, c) even while no noticeable changes were detected in RPB1 phosphorylation patterns (Supplementary Fig. 6c). This difference was rescued when Corin, a dual inhibitor for LSD1 and HDAC1/2^38^ was added to the cells 2 h prior EU labeling (Fig. 6b, c), consistent with RCOR1 being in a functional complex with LSD1 and HDAC1/2. To confirm that this was an effect of inhibiting LSD1 or HDACs, we added TCP (LSD1 inhibitor) or Entinostat (HDAC1/2 inhibitor) 2 h prior to EU labeling and observed that Entinostat treatment rescued transcriptional activity (Fig. 6b, c). With a more specific LSD1 inhibitor, GSK-LSD1, there was also rescue, albeit at a less robust level (Fig. 6d). These data confirm that RCOR1 suppresses transcription in a functional complex with LSD1 and HDAC1/2.

Conversely, when RCOR1 was knocked down (KD), we observed a significant increase in the fluorescent signal (Fig. 6e), even when phosphorylation levels of RPB1 were not affected (Supplementary Fig. 6d), highlighting RCOR1 as a negative regulator of de novo transcription. To confirm this, we blocked transcription in promoter-proximal pausing using Flavopiridol in RCOR1 KD cells for 2 h, and after washing it out we followed EU incorporation at 0, 20, and 40 min (Fig. 6f). The recovery of transcriptional activity was significantly faster in RCOR1-deficient cells. In addition, in vitro transcription assays using nuclear extracts of cells treated with Corin showed significantly higher activity than the control assays (Fig. 6g). These data support RCOR1 as a general repressor of gene expression in short time scales and that altering its levels can impact transcription globally.

### The RCOR1 complex regulates RNA POL-II by deacetylating the CTD at lysine 7

**Fig. 7:**
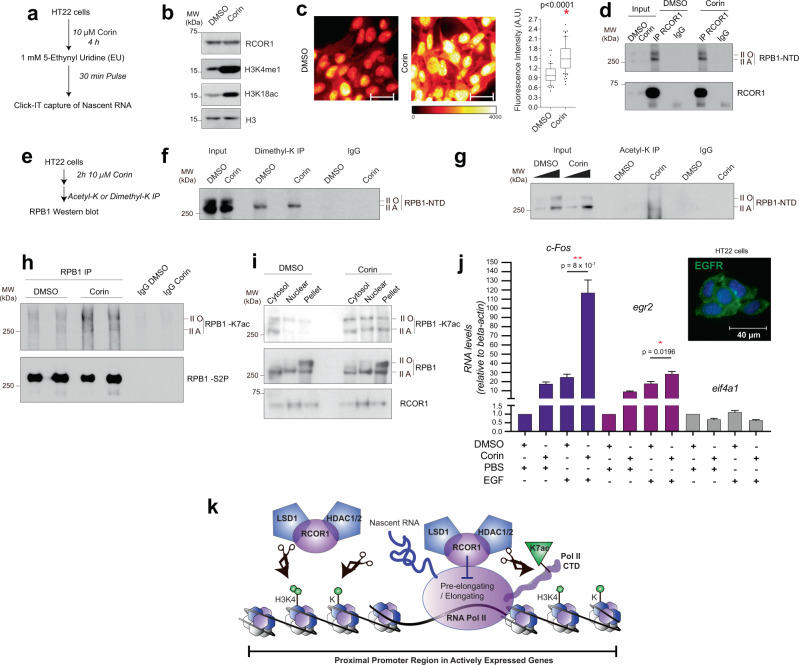
RCOR1 globally represses transcription of newly synthesized transcripts. **a** Scheme depicting the strategy used to label nascent transcripts in Corin-treated HT22 cells. **b** Western blot analysis showing the effect on H3 modifications after treating HT22 cells with Corin. Panels are representative of two independent experiments. **c** Pseudocolor images showing labeling of nascent transcripts in HT22 cells. Scale bar represents 40 µm. Right box plots showing quantitation of fluorescence intensity of nascent transcripts. Red asterisk indicates *p* < 0.05 statistical significance compared to DMSO treated cells according to non parametric, two-tailed, Mann–Whitney test. **d** RCOR1 immunoprecipitation showing co-immunoprecipitated RPB1 levels after treating HT22 cells with Corin. This experiment was performed once. **e** Scheme depicting the strategy used to analyze RPB1 post-translational modifications after Corin treatment. **f** Analyses of RPB1 dimethylation under Corin treatment. Panels are representative of two independent experiments. **g** Analyses of RPB1 acetylation under Corin treatment. Panels are representative of two independent experiments. **h** RPB1-K7ac western blot after performing RPB1 immunoprecipitation from two different replicates of DMSO or Corin treated HT22 cells. RPB1-S2P was assayed as a control showing the precipitation of RPB1. Panels are representative of three independent experiments. **i** Subcellular fractionation of HT22 cells treated with DMSO or Corin. Cytosol, Nuclear soluble, and chromatin fractions were analyzed for RPB1-K7ac, RPB1, and RCOR1. Panels are representative of two independent experiments. **j** Graph depicting the RNA levels of c-Fos, egr2 (genes induced by EGF), and eif4a1 (negative control) when DMSO or Corin-treated HT22 cells are stimulated with EGF or PBS. Upper right corner shows the positive immunostaining of the EGF receptor (EGFR) in HT22 cells. Results are shown as columns depicting the mean plus SEM. Red asterisks indicate **p < 0.001 and *p < 0.05 statistical significance compared to DMSO treated, EGF-stimulated cells by unpaired, two-sided *T*-tests. This experiment was performed using three biological replicates with two technical replicates each. **k** Working model. Source data are provided as a Source Data file.

Given that POL-II’s CTD contains heptapeptide YSPTSPS tandem repeats that are dynamically phosphorylated during the transcription cycle^34,35^, we wondered if enzymatic activities of the RCOR1 complex could impact RPB1 acetylation and methylation that are associated with early stages of transcription^39,40^. We treated HT22 cells with Corin (Fig. 7a), and saw both accumulation of the RCOR1 complex substrates H3K4me1 and H3K18ac (Fig. 7b) and upregulated global transcription (Fig. 7c). This inhibitor did not prevent RCOR1 from interacting with RNA POL-II subunit RPB1 (Fig. 7d), suggesting that the associated enzymes are not required for RCOR1 recruitment to the transcriptional machinery. We then carried out immunoprecipitation of acetylated or dimethylated proteins in cells treated with Corin (Fig. 7e) and found that Corin treatment did not change the dimethylation levels of RPB1 (Fig. 7f), but increased RPB1 acetylation (Fig. 7g). This led us to ask if the observed RPB1 hyperacetylation occurred at lysine 7 of the RPB1-CTD (RPB1-K7ac), which is enriched in promoter-proximal pausing and elongation steps of transcription^40^. Corin induced an accumulation of RPB1-K7ac in both isoforms of RPB1 (Fig. 7h). Although Corin did not change the subcellular distribution of RPB1, it enhanced its K7 acetylation on cytosolic, nuclear, and chromatin pools of RPB1 (Fig. 7i).

Finally, we wondered if the RCOR1 complex could play a role in the regulation of EGF-induced transcription, as it has been reported that *c-Fos* and *egr2* respond to this stimulation in an RPB1-K7ac dependent way^40^. We found that Corin by itself was enough to upregulate their transcription (Fig. 7j), suggesting the RCOR1 complex as a regulator of RPB1-K7ac dependent genes. More importantly, Corin enhanced the EGF-mediated induction of both transcripts confirming that the RCOR1 complex is repressing genes whose transcription is dependent on RPB1-K7ac. Altogether, our data indicate that RCOR1 represses transcription in two ways—non-canonically through deacetylation of RNA POL-II’s CTD at lysine 7, as well as canonically by erasing transcriptionally permissive histone modifications (Fig. 7k).

## Discussion

Here we have unveiled RCOR1 as a transcriptional rheostat for gene repression within active chromatin. Indeed, despite being a repressor of transcription, we first observed an unexpected association with highly expressed genes and euchromatin. Further investigation revealed specific enrichment at promoter-proximal regions. Biochemical analyses then demonstrated a specific engagement of RCOR1 with the transcriptional machinery after promoter clearance. Importantly, RCOR1 represses transcription by regulating RNA POL-II activity, either at the level of pause-release or productive elongation. This is dependent on both canonical activities on histone substrates and non-canonical activities on the POL-II CTD—both of which result in transcriptional downregulation, even within active genes (Fig. 7k).

### A distribution equilibrium between cytosolic, nucleoplasm, and chromatin for the RCOR1 complex subunits

RCOR1 complexes are distributed in cytosolic and nuclear soluble fractions, in addition to their chromatin-bound state. Since we detected the complex subunits both in cytosol and nucleus in single cells by microscopy, the biochemical subpopulations detected by fractionations reflect the coexistence of different complex states inside cells, possibly as the result of a dynamic distribution equilibrium between soluble cell compartments and chromatin at steady state. Curiously, the colocalization of RCOR1 with Hoechst was barely detectable, suggesting that the distribution equilibrium of RCOR1 complexes must be occurring mostly at euchromatin regions. Since we detected an abundant chromatin-bound RCOR1 pool that resisted high-salt extractions, further work will be needed to determine how the complex is stabilized in chromatin. In the same way, future studies will explore if their presence in cytosolic fractions is due to the synthesis of new complex-subunits and/or it is playing a non-canonical role on extranuclear demethylation and deacetylation reactions occurring on newly synthesized histones^41–43^ as well as on non-histone cytosolic proteins^44,45^.

### Enrichment of RCOR1 at accessible and transcribed chromatin

When chromatin is digested by MNase, the digestion reaction preferentially occurs at nucleosome-free regions. Kinetically, the first products are chromatosomes (nucleosomes containing histone H1), which are then further digested to produce free nucleosomes, releasing histone H1 and short sequences of linker DNA^46,47^. However, since chromatin architecture is not homogeneous, MNase-accessible genomic regions are digested first, and their products are enriched in partially digested chromatin^48,49^. We showed that RCOR1 was mostly distributed in fractions spanning nucleosome-free regions, mono, and di nucleosomes, suggesting that a substantial population of RCOR1 complexes is enriched in MNase-accessible chromatin, and it might be stabilized by linker DNA and/or by histone H1. In this context, it was shown that linker DNA stabilizes the binding of the RCOR1-LSD1 complex to nucleosomal substrates^21^, and a recent report showed structural evidence of LSD1 direct binding to internucleosomal DNA^22^. How this complex would display crosstalk with factors that bind linker DNA remains unexplored.

In addition to its prevalence on MNase-accessible chromatin, we presented biochemical, microscopical and chromosome 3D modeling evidence showing that RCOR1 interacts and colocalizes with nucleosomes harboring transcriptionally permissive histone modifications. Our findings may reflect a common role of RCOR1 complexes since bioinformatic analyses on human K562 cells revealed that the complex is enriched at proximal promoters and 5’UTRs of chromatin marked by co-activators and histone modifications that are permissive to transcription. Consistently, we found a positive correlation between RCOR1 occupancy and gene expression, suggesting that the genes that contain higher levels of RCOR1 are more frequently transcribed. We also showed a negative correlation with heterochromatin marks, supporting the exclusion of the complex from heterochromatin domains.

Many questions emerged regarding the unexpected role of a co-repressor complex at euchromatin domains. It has been previously suggested that the presence of different HDAC enzymes at active genes can reflect the need of histone deacetylation reactions to reset genes after transcription, since HDAC inhibition increases histone acetylation on active promoters^50^. In addition, as we detected RCOR1 occupying gene body segments on genes with the highest RCOR1 occupancy, we propose that the complex could be acting on the resetting of histone modifications after transcription in regions where transcription elongation occurs. In this sense, it has been shown that H3K9ac can recruit factors required for RNA POL-II-mediated elongation, and HDAC inhibition leads to impaired transcriptional elongation^51,52^. Interestingly, NuRD, another co-repressor complex that functions with histone deacetylation, has also been detected at active regions as an acetylation regulator^53,54^. In this regard, the dual inhibition of LSD1 and HDAC1 in the context of the RCOR1 complex by Corin has revealed increased H3K27ac and H3K4me1 levels on both the TSS and gene body segments of active genes^55^. Since Corin rescued the loss of transcription produced by overexpression of RCOR1, and also impacted the acetylation of RPB1-K7, additional studies are needed to test the dynamics of the RCOR1 complex when regulating nascent transcription by modulating RPB1 acetylation and histone modifications.

### Insights into the recruitment of RCOR1 at specific transcription stages

Our study revealed a specific interaction between RCOR1 and RNA POL-II occurring in chromatin, which provides a mechanism for RCOR1 recruitment to active genes. Interestingly, this interaction was sensitive to chemical inhibition of different transcription stages and suggested that RCOR1 interacts with RNA POL-II after initiation and before productive elongation. These data support a model where the RCOR1 complex might be participating in the removal of histone modifications and RNA POL-II K7 acetylation in a co-transcriptional way. The acetylation of lysine residues in CTD-YSPTSPK non-canonical repeats of RPB1 has been detected both in promoter-proximal paused RNA POL-II and in elongating RNA POL-II^40,56^, and we showed RCOR1 complex as an in vivo eraser of RPB1-K7ac.

Given that the chemical inhibition of the RCOR1 complex by Corin results in an increase of transcriptionally permissive marks on its target genes^38,55^, and our results suggested that RCOR1 may have a role regulating transcription of genes that are highly expressed, we studied the effect of modulating RCOR1 protein levels on transcripts that were synthesized in a short time scale (30 min). We showed that RCOR1 upregulation globally represses transcription. This observation suggests that RCOR1 may work as a negative global regulator of highly expressed genes by presumably stabilizing promoter-proximal pausing, slowing down the transcription speed, or other parameters of transcriptional bursting, such as burst size or frequency, as it has been reported for HDACs^57^. According to the transcriptional bursting hypothesis, mammalian gene expression occurs in pulses known as bursts as genes can switch from an inactive to an active state depending on stochastic collisions of chromatin regulators of transcription rather than relying on the deterministic nature of biochemical pathways, we can infer from cell population studies^58–60^. The cascade of events that marks the transition from the ON to OFF state in active transcription has not been clarified yet, but our evidence suggests that RCOR1 might be playing a role in it. Finally, we highlight our discovery of non-canonical roles for RCOR1 arising from its interaction with active RNA POL-II, which expands the scope of processes where RCOR1 can be involved in, from chromatin remodeling to regulation of transcriptional dynamics.

## Methods

### Cell culture

HT22, HeLa and HEK293-T cells were cultured in Dulbecco’s Modified Eagle’s Medium (DMEM, Gibco) supplemented with 10% fetal bovine serum (FBS, Gibco) and 1% Penicillin/Streptomycin. Cells grew at 37 °C in an atmosphere containing 5% CO_2._ Transfections were carried out using Lipofectamine 3000® (Invitrogen) according to the manufacturer’s instructions. Plasmids and Lipofectamine were mixed in ratios of 3 μL Lipofectamine per µg of DNA, and the resulting complexes were dropped to growing cells after 20 min incubation at room temperature. Cells were harvested 24 h after transfection. For knockdown experiments, cells were transfected for 24 h with RCOR1 siRNA mix (Dharmacon, M-014076-01-0010). siRNAs were transfected at a ratio of 33 pmol per µL of Lipofectamine 2000®. Then, western blots were performed to check protein levels. For chemical inhibition of different stages of transcription 1-h treatments with THZ1, Flavopiridol, Cordycepin, or Actinomycin D were performed as previously described^36^.

### Antibodies

Mouse anti-RCOR1 (NeuroMab, 75-039, WB 1:1000); Mouse anti-RCOR1 (BD Biosciences, #612146, IF: 1:100), Rabbit anti-RCOR1 (Abcam, ab183711, IP: 2 µg) rabbit anti-LSD1/KDM1 (Abcam, ab17721, WB: 1:1000, IF: 1:500, IP: 2 µg); rabbit anti-HDAC1 (Abcam, ab7028, WB: 1:5000, IF: 1:1000, IP: 2 µg); mouse anti-HDAC2 (Abcam, ab51832, WB: 1:1000, IF: 1:500); rabbit anti-EZH2 (Cell Signaling Technology #5246, WB: 1:1000), rabbit anti-HP1α (Cell Signaling Technology, #2616, WB 1:1000), rabbit anti-RPB1 NTD (Cell Signaling Technology, #14958, WB: 1:1000, IF: 1:500, IP: 2 µg), rabbit anti-phospho-RPB1 CTD (Ser2) (Cell Signaling Technology, #13499 WB: 1:1000, IF: 1:500), rabbit anti-phospho-RPB1 CTD (Ser5) (Cell Signaling Technology, #13523, WB: 1:1000, IF: 1:500), rabbit anti phosphor-RPB1 CTD (Ser7) (Cell Signaling Technology, #13780, WB: 1:1000, IF: 1:500), rabbit anti-H3 (Novus Biologicals, NB500-171, WB: 1:1000), rabbit anti-H3K4me1 (Cell Signaling Technology, #5326, WB:1:1000), rabbit anti-H3K4me2 (Cell Signaling Technology, #9725, WB: 1:5000); rabbit anti-H3K4me3 (Cell Signaling Technology, #9751, WB: 1:1000, IF: 1:500), rabbit anti-H3K9me3 (Abcam, ab8898, IF: 1:1000); rabbit anti-H3K27me3 (Active Motif, #39055, WB:1:1000, IF: 1:250), rabbit anti-H3K9ac (Cell Signaling Technology, #9649, WB: 1:1000), rabbit anti-H3K18ac (Cell Signaling Technology, #13998, WB: 1:1000, IF: 1:500), mouse anti-GAPDH (Cell Signaling Technology, #5174, WB: 1:10000), rabbit anti-H3R2me2a (Epigentek, #A-3714: WB: 1:500), rabbit anti-HA tag (Cell Signaling Technology, #3724, WB:1:5000), rabbit anti-NCOR2 (Invitrogen PA1-843, WB: 1:1000), rabbit anti-MEK1/2 (Cell Signaling Technology, #8727, WB: 1:1000), mouse anti-β Tubulin (Sigma Aldrich #T5201, WB: 1:5000), rabbit anti EGFR (Cell Signaling Technology, #4267, IF: 1:500), rabbit anti Lamin B1 (Abcam, #ab65986, WB: 1:5000), rabbit anti-acetyl-Lysine (Active Motif, #91315, IP: 5 µg), rabbit anti-dimethyl-Lysine (Cell Signaling Technology, #14117, IP: 1:100).

### Cell immunofluorescence

Coverslips-grown cells were fixed with 4% paraformaldehyde in PBS for 15 min. After three washes with PBS, cells were permeabilized by 5 min incubation with 0.25% Triton-X100 in PBS and blocked by 1-h incubation with 3% BSA in PBS. After incubation with primary antibodies in a humid chamber at room temperature during 1-h, extensive washes in 1X PBS, coverslips were incubated with secondary anti-rabbit IgG conjugated to Alexa 488 and anti-mouse IgG conjugated Alexa 594, respectively. All incubations were performed at room temperature and primary/secondary antibodies incubations were done in humid chambers. Coverslips were mounted on DAKO Fluorescence Mounting Medium (Agilent) after counterstaining with 1 µg/mL Hoechst 33342. Images were acquired on a Zeiss LSM 800 Airyscan confocal microscope, with Airyscan acquisition mode and super-resolution processing was performed.

### Image analyses

Colocalization analyses were performed using ImageJ software (NIH, Baltimore, MD) by using the JACoP (Just another colocalization plugin) plugin^61^ to determine Van Steensel parameters for single Z-stacks of images. Fluorescence intensity was measured from images using the raw integrated densities of each cell over background measurements. Intensities were normalized as the percentage of total fluorescence counts.

### Subcellular fractionation and sequential extraction of chromatin-bound proteins

Cells were washed twice in 1X PBS and collected by trypsinization. Trypsin was inactivated with complete growth media. Then, cells were centrifuged and washed twice in 1X PBS. The cell pellet was incubated for 10 min in 5 volumes of hypotonic buffer (10 mM Tris, pH 7.9, 1.5 mM MgCl_2_, 10 mM KCl, 0.5 mM DTT, 0.2 mM PMSF, and 1X protease inhibitor complex (Roche)), cells were then centrifuged, resuspended in 2 volumes of hypotonic buffer and finally lysed by mechanical homogenization. The supernatant was supplemented with additional 30 mM Tris pH 7.9, 140 mM KCl, and 3 mM MgCl_2_, then it was cleared by centrifugation at 15,000 × *g* for 30 min at 4 °C and stored as a cytosolic extract. Nuclei were collected and washed 3 times in hypotonic buffer, then were sequentially resuspended in nuclear extraction buffers (20 mM Tris, pH 7.9, 25% glycerol, 1.5 mM MgCl_2_, 0.2 mM EDTA, 0.5 mM DTT, 0.5 mM PMSF, and 1X protease inhibitor complex (Roche)), starting with 100 mM NaCl and increasing salt concentration by steps of 100 mM until 600 mM NaCl was reached. For each step, nuclei were incubated for 7 min at 4 °C and then centrifuged at 4000 × g for 5 additional minutes. Supernatants were collected and cleared by centrifugation at 15,000 × *g* for 30 min at 4 °C. Fractions were analyzed by western blot, loading equal volumes of each one.

### Immunoprecipitation

Cells were lysed in Immunoprecipitation buffer (20 mM Tris-HCl pH 7.5, 150 mM NaCl, 1 mM EDTA, 1 mM EGTA, 1% NP40, 1 mM PMSF, 1 µg/mL leupeptin, and 1 µg/mL aprotinin). Sonication was applied to improve the solubilization of chromatin-bound material, and the homogenate was cleared by centrifugation at 12,000 × *g* for 20 min at 4 °C. Immunoprecipitation was performed using 50 uL SureBeads Protein A Magnetic beads (BioRad) and 1–2 µg of primary antibody every 700 µg of protein. Immunocomplexes were magnetically separated after 12 h of incubation. Then, beads were extensively washed against CoIP buffer and immunocomplexes were eluted by boiling the beads in 1X Laemmli Sample Buffer (60 mM Tris-HCl pH 6.5, 2% SDS, 5% glycerol, and 1.8 M β-mercaptoethanol).

### Western blot

Whole-cell extracts were prepared by homogenization in RIPA buffer (Millipore) in the presence of 1 mM PMSF, 1 µg/mL leupeptin, and 1 µg/mL aprotinin as protease inhibitors. Sonication was applied to optimize lysis and protein extraction. Protein content was measured by the Micro-BCA method (Thermo-Scientific). Protein samples were mixed with 5X Laemmli Buffer and denatured at 100 °C for 5 min. SDS-PAGE was run at constant 80–100 V in denaturing running buffer (25 mM Tris, 200 mM glycine, 1% SDS) and transferred to 0.45 µm pore-sized PVDF membranes at constant current 400 mA in transfer buffer (25 mM Tris, 200 mM glycine). Membranes were blocked 1 hr with 5% non-fat dry milk in TBS-Tween 20 buffer (25 mM Tris-HCl pH 7.6, 275 mM NaCl, 0.1% Tween 20). Incubation with primary antibodies was carried out overnight at 4 °C, and secondary antibody incubation was performed for 1 h at room temperature. Chemiluminescence development (ECL, Amersham) was used to detect protein bands.

### MNase digestion and sucrose gradient ultracentrifugation

2 × 10^8^ HT22 nuclei were partially digested with 20 U micrococcal nuclease (Worthington, LS004798) during 15 min at RT in 20 mM Tris-HCl pH 7.5, 70 mM NaCl, 20 mM KCl, 5 mM MgCl_2_, 3 mM CaCl_2_, and protease inhibitor cocktail (Roche). The reaction was stopped by adding 2 mM EDTA. Digestion products were extracted by incubating the suspension with 300 mM NaCl and centrifuged at 15000 × *g* during 15 min at 4 °C. Supernatants were loaded on 5–50% sucrose gradients and ultracentrifuged at an average speed of 300,000 × *g* during 16 h.

### Analysis of ChIP-seq data sets

RCOR1, LSD1, HDAC1, p300, Pol-II, and various histone marks ChIP-seq data and peak information for K562 cell line were obtained from the ENCODE project. Deeptools program (v3.1.2) was used for peak-centric and gene-centric meta-analysis for ChIP-seq and RNA-seq data^62^. K-means clustering was performed with the parameter *k* = 4 by choosing the minimal cluster number that allows to recapitulate all major patterns and features in different clusters. Peak overlap analysis between RCOR1, LSD1, and HDAC1 was performed using the R package ChIPpeakAnno^63^. MultiBigwigSummary program in deeptools (v3.1.2) package was used for analyzing the global correlation between the signal distributions of RCOR1 and various histone marks by calculating spearman correlation in 10 kb bins throughout the genome. Gene Ontology analysis was performed by using the analysis tools in gene ontology website (http://geneontology.org).

### Analysis of genomic feature enrichment

The analysis for the enrichment of RCOR1 peaks over genomic features (promoters, intergenic, etc) was done by mapping peaks to the annotated genome with CEAS Python package 1.0.2.^64^

### Distance distribution analysis

The distance distribution analysis of nearest bidirectional gene transcription start site (TSS) or nearest bidirectional transcript for each cluster was done by calculating the distance from the TSS of each annotated gene to the closest bidirectional gene TSS. Box plots were drawn by R.

### High resolution chromosome modeling

Simulations described in this work were performed using the Large-Scale Atomic/Molecular Massively Parallel Simulator (LAMMPS)^65,66^. The initial structure consisted of *N* = 12,592 beads that form a linear polymer chain as a result of a self-avoiding random walk (SARW); these beads correspond to ~63 Mb. Experimental HiC constraints were used directly to form harmonic bonds between interacting particles, and those were forced to form connected pairs via the Monte Carlo algorithm. Once all the bonding constraints were satisfied, the bonds are preserved, and the structure was allowed to equilibrate using Brownian Dynamics with implicit solvent. Defined for the simulation were pair interactions between bonded particles using FENE and Lennard–Jones potentials:

$${U}_{{FENE}-{LJ}}=-\frac{1}{2}\kappa {R}_{0}^{2}{{{{{\rm{ln}}}}}}\left[1-{\left(\frac{{{{{{\rm{r}}}}}}}{{{{{{{\rm{R}}}}}}}_{0}}\right)}^{2}\right]+\left\{\begin{array}{c}4{\varepsilon }^{* }\left[{\left(\sigma /r\right)}^{12}\,-\,{\left(\sigma /r\right)}^{6}+\left.{\varepsilon }^{* }\right],\,r\le {2}^{1/6}\sigma \right.\\ 0,\,r \, > \, {2}^{1/6}\sigma \end{array}\right.$$

where *σ* is a dimensionless quantity that characterizes distance, and the optimal parameter set of the maximum bond length *R*_0_ = 20 *σ* and the spring constant *κ* = 30 ε^*^/*σ*^2^. We choose the repulsive LJ strength ε^*^ = 1 in non-dimensional units for this bonded potential, which makes the equilibrium bond length *r*_bond_ = 0.99 *σ* yet allowing the bond to be stretched up to 20 *σ*.

For nonbonded atoms, only the repulsive part of the Lennard–Jones interaction potential was used:

$${U}_{{LJ}}=\left\{\begin{array}{c}4\varepsilon \left[{\left(\sigma /r\right)}^{12}\,-\,{\left(\sigma /r\right)}^{6}+\left.1/4\right],\,r\le {2}^{1/6}\sigma \right.\\ 0,\,r \, > \, {2}^{1/6}\sigma \end{array}\right.$$

Finally, we used harmonic constraints originating from experimental HiC data, ${U}_{{HiC}}=K{\left(r-{r}_{0}\right)}^{2}$, where *K* = 1 *ε/σ*^*2*^ and r_0_ = 2.2 *σ*. This ensures that the initial random structure of the polymer chain converges and satisfies the constraints originating from Hi-C experiments. The average simulation temperature was controlled by the Langevin thermostat (kept constant at T_start_ = T_end_ = 1 in dimensionless units, with the damping coefficient set to 1 τ^−1^. A timestep of 0.01τ was used, where τ is the reduced (Lennard-Jones) time-a measure of how long it takes for the particle to move across its own size, defined as $\tau =\sigma \sqrt{\left(m/u\right)}$, where *m* is the characteristic mass, and *u* is the intrinsic energy of the system that is the same as parameter ε* in the spring constant κ.

### Imaging of nascent transcripts

Cells were seeded on coverslips at 50% confluency. 16 h later, cells were incubated with 1 mM 5 ethynyl uridine (EU) during 30 min. Right after 30 min, cells were fixed in 4% paraformaldehyde—PBS and permeabilized in 0.5% Triton X100—PBS at RT. Coverslips were then washed with PBS and biotinylation reactions were proceeded with Alexa Fluor 594—conjugated sodium azide in the presence of CuSO_4_. Finally, coverslips were extensively washed, counterstained with Hoechst, and mounted with DAKO. Image acquisition was performed on a Nikon 90i Microscope equipped with 603/1.4 NA. VC Objective lens, Orca ER CCD Camera (Hamamatsu) and Volocity Software (Perkin Elmer). Fluorescence intensity was calculated for each condition using ImageJ as the ratio of background-subtracted Raw Integrated Density and each cell nuclear area. Results are expressed in arbitrary units (A.U.) as the result of normalizing the ratio of each condition against the ratio of the control.

### In vitro transcription from soluble nuclear extracts

Nuclear extracts were prepared as already described using a 420 mM NaCl containing buffer on HeLa cells treated for 2 h with DMSO or 10 µM Corin. Extracts were dialyzed 2 h at 4 °C against 1000 volumes of 20 mM HEPES pH 7.9, 20% Glycerol, 100 mM KCl, 0.2 mM EDTA, 0.5 mM PMSF, and 0.5 mM DTT. 40 µg of proteins were assayed for transcriptional activity in a buffer containing 10 mM HEPES pH 7.9, 10% Glycerol, 0.5 mM DTT, 0.1 mM EDTA, 60 mM KCl, 12 mM MgCl_2_, 0.4 mM CTP/UTP/GTP, 15 µM cold ATP, and 0.2 µCi/uL of 32P-α-ATP. The linear template used as a substrate consisted on the GAPDH coding sequence including the following promoter upstream: 5′-CCCCCTATTGACGTCAATGACGGTAAATGGCCCGCCTGGCATTATGCCCAGTACATGACCTTATGGGACTTTCCTACTTGGCAGTACATCTACGTATTAGTCATCGCTATTACCATGGTGATGCGGTTTTGGCAGTACATCAATGGGCGTGGATAGCGGTTTGACTCACGGGGATTTCCAAGTCTCCACCCCATTGACGTCAA (CRE ELEMENT) TGGGAGTTTGTTTTGGCACCAAAATCAACGGGACTTTCCA AAATGTCGTAACAACTCCGCCCCATTGACGCAAATGGGCGGTAGGCGTGTACGGTGGGAGGTCTATATAA (TATA Box) GCAGAGCTCTCTGGCTAACTAGAGA ACCCACTGCTTACTGGCTTATCGAAATT-3′.

### EGF stimulation

HT22 cells were cultured in 1% FBS containing DMEM, after 2 h cells were treated with DMSO or 10 µM Corin. After 3 h 30 min, cells were stimulated with PBS or 12.5 ng/mL EGF during 30 min. Cells were immediately harvested and total RNA was extracted using Trizol. The following primers were used to detect specific transcripts by RT-qPCR:

*c-Fos* Forward: 5′-CCAGTCTGCTGGGGCTTAC-3′

Reverse: 5′-GCAGCCATCTTATTCCGTTC-3′.

*Egr2* Forward: 5′-TTGACCAGATGAACGGAGTG-3′

Reverse 5′-ACCAGGGTACTGTGGGTCAA-3′

*Eif4a1* Forward: 5′-CGGAGATATGGACCAAAAGG-3′

Reverse: 5′-CTGTTGGTGGGAAGGTCATAG-3′

### Exon inclusion frequency by relative quantity fluorescent-PCR analysis (Rqf-PCR)

Total RNA was isolated from HT22 using Trizol reagent (Invitrogen Life Technologies), and reverse transcribed using MMulV (Thermo Scientific). Rqf-PCR was performed as previously described^67^. qPCR primers were designed to amplify 8a exon region: Ex8_FW: 6-Fam-5′TCCCATGGCTGTCGTCAGCA3′; Ex11_RV:5′CTACCATTTCATCTTTTTCTTTTGG3′. The ratio of uLSD1/nLSD1 was analyzed by peak scanner software v.1.0.

### Statistical analyses

Proximity ligation assays were plotted as violin plots highlighting quartiles for each dataset with continuous lines and median values as a dashed line. Statistical significance was checked using Graphpad Prism by confirming normal distribution of data by D’Agostino & Pearson, Shapiro–Wilk, and KS normality tests. Data was subjected to an ordinary, unpaired, one-way ANOVA test with 99% confidence intervals, individual p values are present on each panel. For EU-incorporation experiments, data are shown as box plots, with each box extending from the 25th to 75th percentiles and whiskers extend from the 5th to the 95th percentile. Individual dots show datapoints outside whisker limits. Lines represent the median value. Statistical significance was evaluated using Graphpad Prism, by first confirming a data distribution considered not-normal according to D’Agostino & Pearson, Shapiro-Wilk, and KS normality tests. Thus, for testing significance in multiple comparisons, non parametric, unpaired Krukal Wallis tests were run with uncorrected Dunns test (considering that each comparison standed alone). For comparisons between two groups, non parametric, two-tailed Mann–Whitney tests were performed and individual *p* values are shown on each graph. Gene expression analyses were plotted showing the mean ± standard error of the mean (SEM). Student’s *t*-tests were used to analyze statistical significance. The number of replicates and calculated *p*-values is stated in figure legends.

### Reporting summary

Further information on research design is available in the Nature Research Reporting Summary linked to this article.

## Supplementary information

Supplementary information

Peer review file

Description of additional Supplementary File

Supplementary Dataset 1

Supplementary Dataset 2

Reporting Summary

## Data Availability

The data that support this study are available from the corresponding authors upon reasonable request. Publicly available ChIP-seq datasets of K562 cells analyzed in the study include: RCOR1 (GSM935439), RCOR1 (Antibody #2, GSM935385), P300 (GSM935401), LSD1 (GSM1003570), HDAC1 (GSM1003448), POL2 (GSM935358), H3K4me3 (GSM788087), H3K9ac (GSM788082), H3K27ac (GSM733656), H3K27me3 (GSM788088), H3K9me3 (GSM733776), CHD4 (GSM1003510), SIN3A (GSM2424155), EZH2 (GSM1003576), SUZ12 (GSM1003545). RNA-seq data of K562 cells was obtained from accession number GSM958729. Details on files available via ENCODE can be found in Supplementary Table 1. Source data are provided with this paper.

## Source data

Source Data
