# Supplementary material for: Unveiling RCOR1 as a rheostat at transcriptionally permissive chromatin
Source: Nat Commun. 2022 Mar 23;13:1550. doi: 10.1038/s41467-022-29261-0 (PMC8943175; doi:10.1038/s41467-022-29261-0)
Supplement: Supplementary file 1 — Supplementary information [file 41467_2022_29261_MOESM1_ESM.pdf]

SUPPLEMENTARY INFORMATION

Unveiling RCOR1 as a rheostat at transcriptionally permissive chromatin

Carlos Rivera [1,2,3,‡], Hun-Goo Lee [2,3,‡], Anna Lappala[2,3], Danni Wang[2,3], Verónica Noches[1], Montserrat Olivares-Costa[1], Marcela Sjöberg-Herrera[1], Jeannie T. Lee [2,3,#,*] & María Estela Andrés [1,#,*]

[1] Department of Cellular and Molecular Biology, Faculty of Biological Sciences, Pontificia Universidad Católica de Chile. Santiago, 8331150, Chile. [2] Department of Molecular Biology, Massachusetts General Hospital. Boston, MA 02114, USA. [3] Department of Genetics. The Blavatnik Institute, Harvard Medical School. Boston, MA 02114, USA.[‡] These authors contributed equally to this work. [#] These authors agreed to be considered as corresponding authors. [*] To whom correspondence should be addressed: María Estela Andrés, Ph.D. E-mail: mandres@bio.puc.cl. Jeannie T. Lee, MD, Ph.D. E-mail: lee@molbio.mgh.harvard.edu

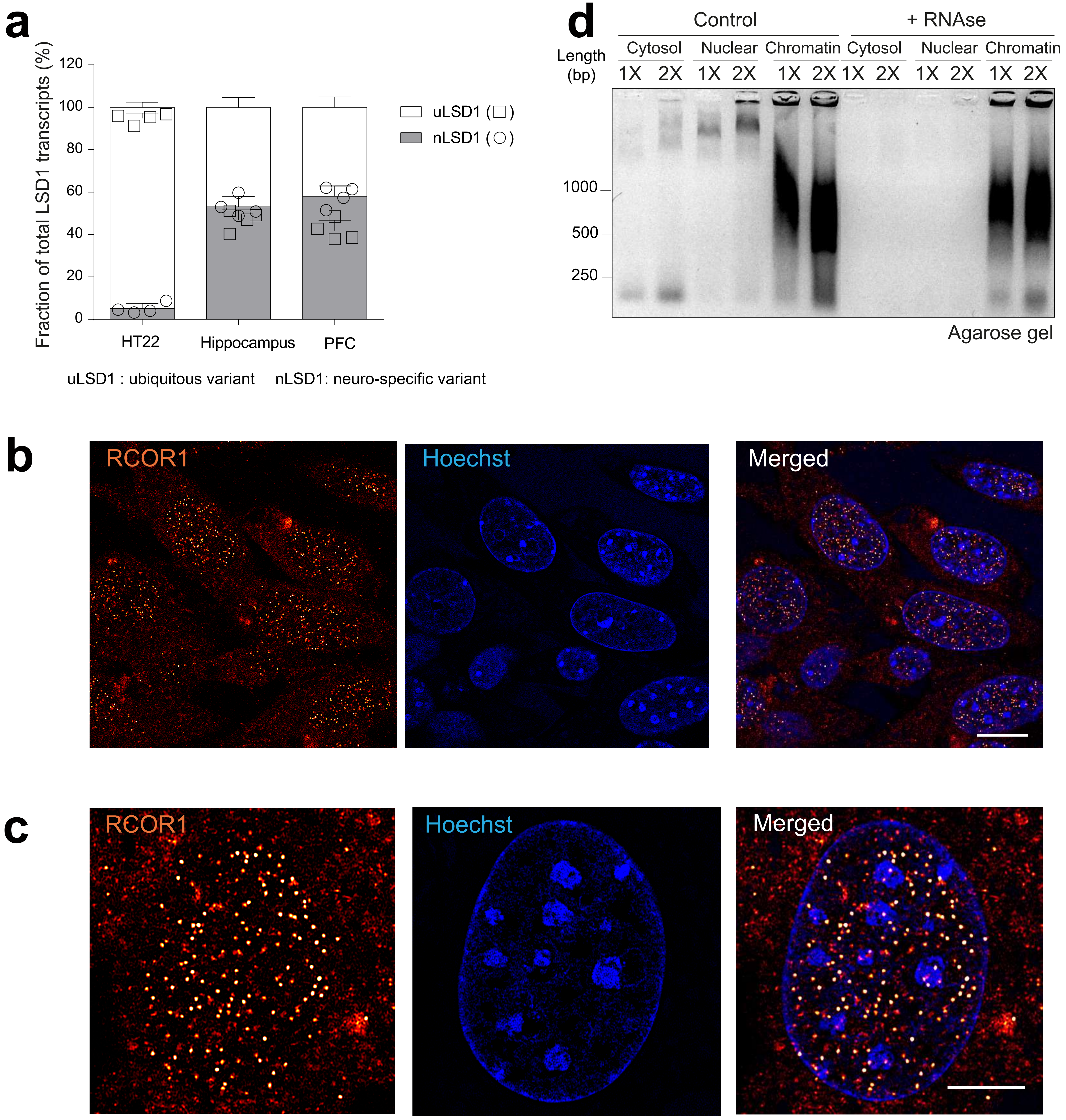
a
Fraction of total LSD1 transcripts (%)
120
100
80
60
40
20
0
HT22
Hippocampus
PFC
uLSD1 (□)
nLSD1 (○)
uLSD1 : ubiquitous variant
nLSD1: neuro-specific variant
d
Control
+ RNAse
Cytosol
Nuclear
Chromatin
Cytosol
Nuclear
Chromatin
Length (bp)
1X
2X
1X
2X
1X
2X
1X
2X
1X
2X
1X
2X
1000
500
250
Agarose gel
b
RCOR1
Hoechst
Merged
c
RCOR1
Hoechst
Merged

Supplementary figure 1. Experimental controls for the use of HT22 cell line, subcellular fractionation and RCOR1 immunofluorescence.

a. Ratio of LSD1/nLSD1 transcripts, expressed as percentage of total LSD1 transcripts on HT22 cells, mouse hippocampus and mouse prefrontal cortex (PFC). Individual values of each experiment are shown as squares or circles as stated next to the grouped columns. Data are shown as mean value with standard deviation.
b. RCOR1 immunostaining obtained at high resolution. Pseudocolored display shows RCOR1 enrichment and distribution in the nuclei of HT22 cells. White bars represent scale bars of 10 µm. Images are representative of four independent experiments.
c. RCOR1 immunostaining obtained at high resolution. Pseudocolored display shows RCOR1 enrichment and distribution in the nuclei of HT22 cells. White bars represent scale bars of 5 µm. Images are representative of four independent experiments.
d. Subcellular fractionation control. Agarose gel analyzing the presence of nucleic acids in cytosolic, nuclear soluble and chromatin fractions. As expected, nucleic acids in soluble fractions disappeared after RNAse A treatment, confirming our protocol yields chromatin-free soluble fractions. Panel is representative of two independent experiments. bp: base pairs.

This figure shows related data to Main Figure 1.

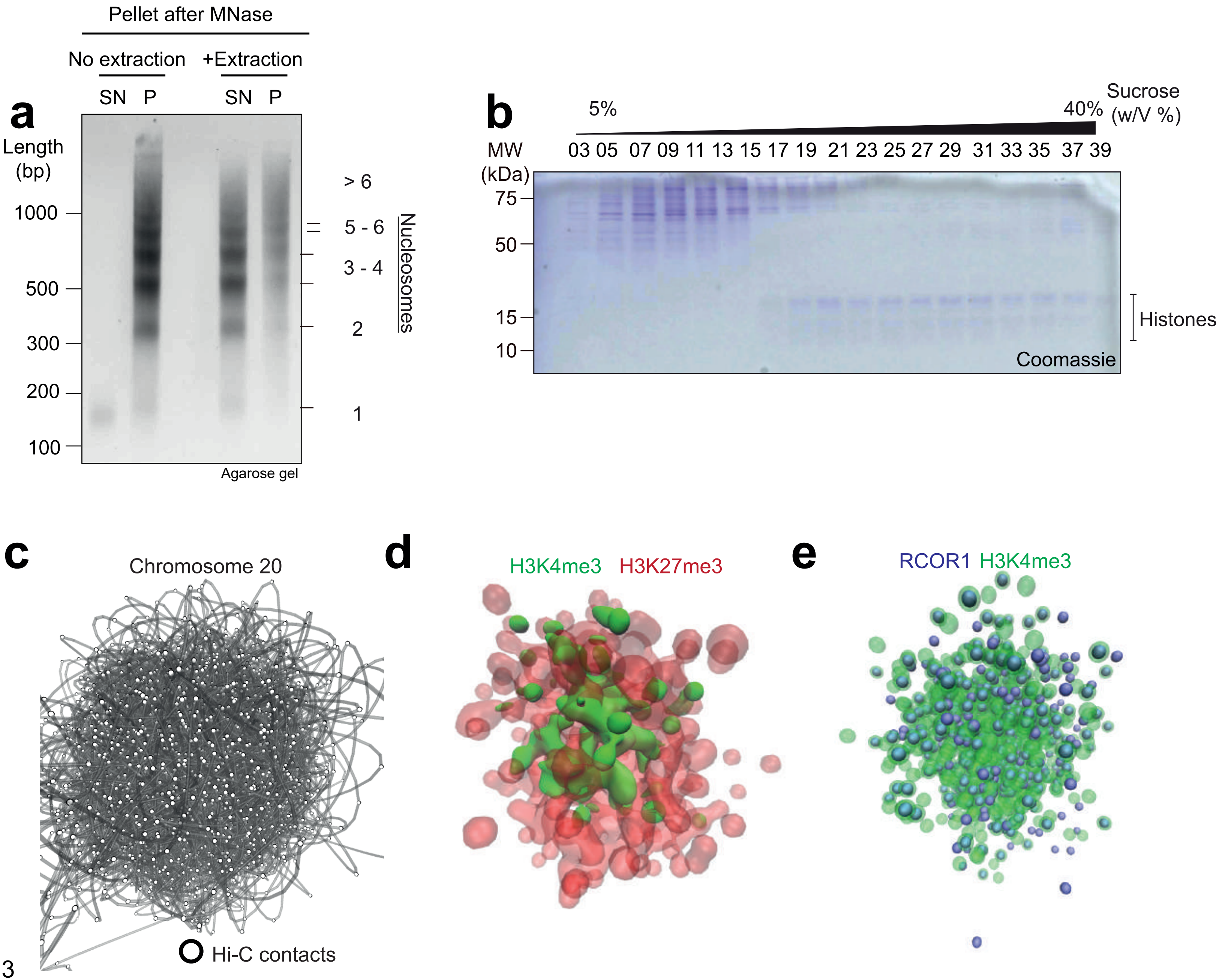
Pellet after MNase
No extraction
+Extraction
SN
P
SN
P
a
Length (bp)
1000
500
300
200
100
> 6
5 - 6
3 - 4
2
1
Nucleosomes
Agarose gel
b
5%
40%
Sucrose (w/V %)
MW (kDa)
03 05 07 09 11 13 15 17 19 21 23 25 27 29 31 33 35 37 39
75
50
15
10
Histones
Coomassie
c
Chromosome 20
Hi-C contacts
d
H3K4me3
H3K27me3
e
RCOR1
H3K4me3

Supplementary figure 2. Experimental controls for main Figure 2.

a. Agarose gel analysis of MNase digestion products before and after solubilization with 300 mM NaCl. SN: Supernatant. P: Pellet. bp: base pairs.
b. Coomassie gel analysis of SDS-PAGE separated proteins obtained from sucrose-gradient experiments after MNase treatment of HT22 chromatin.
c. High-resolution 3D model generated by Monte Carlo simulations with Hi-C contacts as constraints. White dots represent Hi-C contacts.
d. H3K4me3 and H3K27me3 ChIP-seq positions were mapped into the 3D model of chromosome 20 and highlighted with different colors to show the segregation of active and repressive chromosome compartments.
e. Representative image of the significant colocalization between RCOR1 and H3K4me3 in 3D.

This figure shows related data to Main Figure 2.

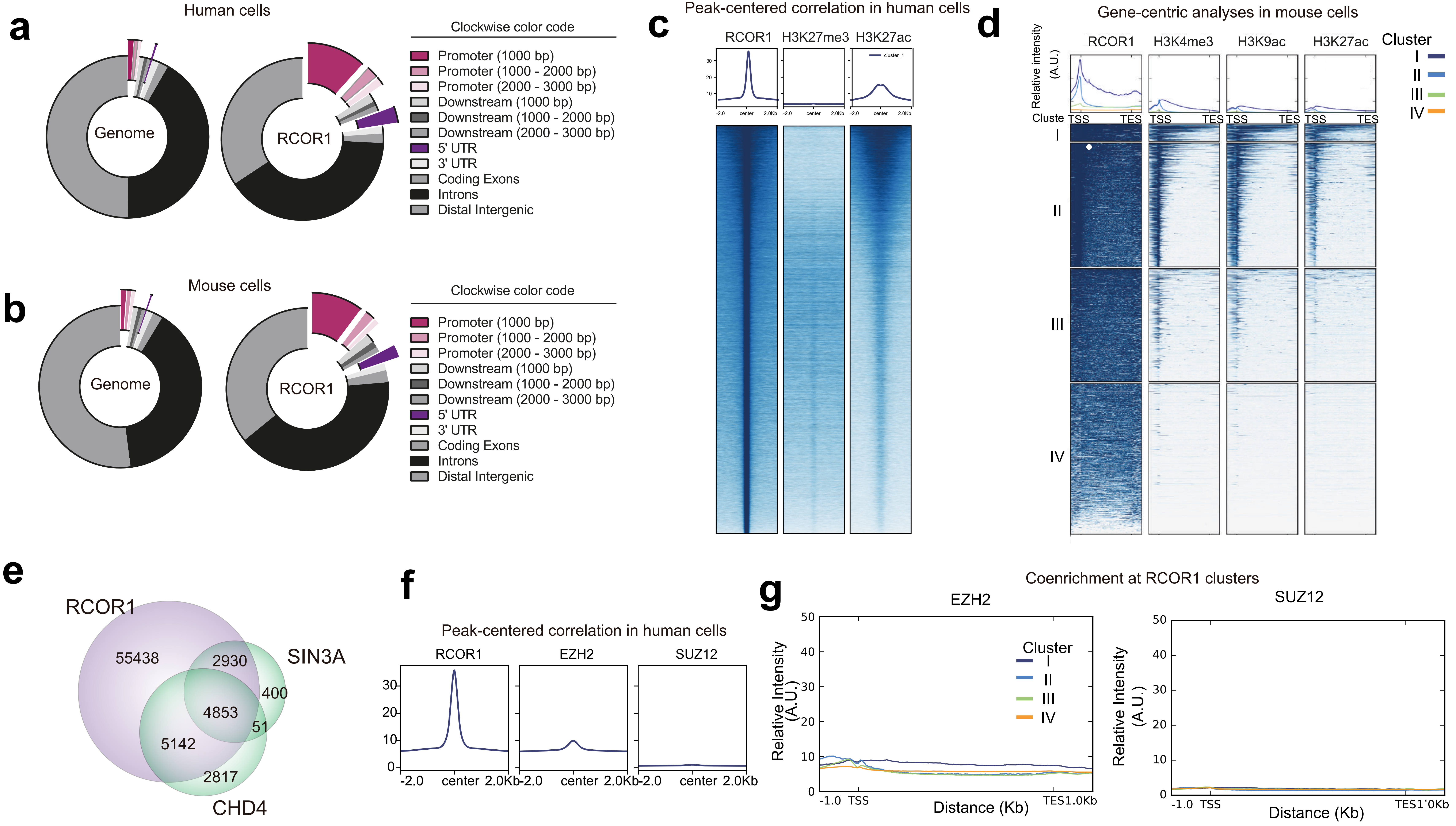

a
Human cells
Genome
RCOR1
Clockwise color code
Promoter (1000 bp)
Promoter (1000 - 2000 bp)
Promoter (2000 - 3000 bp)
Downstream (1000 bp)
Downstream (1000 - 2000 bp)
Downstream (2000 - 3000 bp)
5' UTR
3' UTR
Coding Exons
Introns
Distal Intergenic
b
Mouse cells
Genome
RCOR1
Clockwise color code
Promoter (1000 bp)
Promoter (1000 - 2000 bp)
Promoter (2000 - 3000 bp)
Downstream (1000 bp)
Downstream (1000 - 2000 bp)
Downstream (2000 - 3000 bp)
5' UTR
3' UTR
Coding Exons
Introns
Distal Intergenic
c
Peak-centered correlation in human cells
RCOR1
H3K27me3
H3K27ac
d
Gene-centric analyses in mouse cells
Relative intensity (A.U.)
RCOR1
H3K4me3
H3K9ac
H3K27ac
Cluster
I
II
III
IV
TSS
TES
e
RCOR1
SIN3A
CHD4
55438
2930
400
4853
51
5142
2817
f
Peak-centered correlation in human cells
RCOR1
EZH2
SUZ12
-2.0
center
2.0Kb
g
Coenrichment at RCOR1 clusters
EZH2
SUZ12
Relative Intensity (A.U.)
Cluster
I
II
III
IV
-1.0
TSS
TES
1.0Kb
Distance (Kb)

Supplementary figure 3. Metagenomic analyses of RCOR1 distribution in chromatin (continued from main figure 3).

a. Pie chart of relative abundance of DNA elements in the genome (left) and the occupancy of RCOR1 ChIP-seq peaks on each DNA element on human K562 cells.
b. Pie chart of relative abundance of DNA elements in the genome (left) and the occupancy of RCOR1 ChIP-seq peaks on each DNA element on mouse CH12 cells.
c. Peak-centered, genome-wide analysis of RCOR1 correlation with H3K27me3 and H3K27ac in human K562 cells.
d. Metagene profiling of RCOR1, H3K4me3, H3K9ac and H3K27ac on the four gene clusters in mouse CH12 cells.
e. Venn diagram showing the colocalization of RCOR1, SIN3A and CHD4 (NuRD complex subunit) peaks in human K562 cells.
f. Peak-centered, genome-wide correlation between RCOR1 and PRC2 subunits EZH2 and Suz12.
g. Metagene signal profiling of PRC2 subunits in four clusters described in Figure 3d.

This figure shows related data to Main Figure 3.

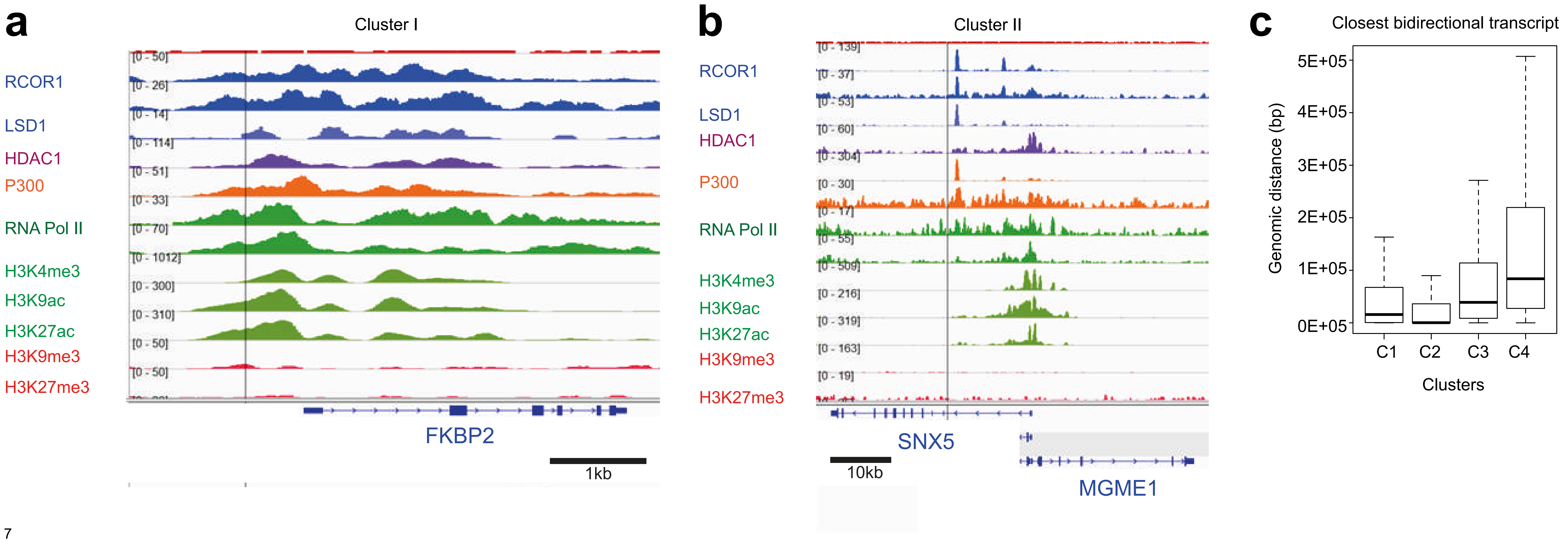
a
Cluster I
RCOR1
LSD1
HDAC1
P300
RNA Pol II
H3K4me3
H3K9ac
H3K27ac
H3K9me3
H3K27me3
FKBP2
1kb
b
Cluster II
RCOR1
LSD1
HDAC1
P300
RNA Pol II
H3K4me3
H3K9ac
H3K27ac
H3K9me3
H3K27me3
SNX5
10kb
MGME1
c
Closest bidirectional transcript
Genomic distance (bp)
5E+05
4E+05
3E+05
2E+05
1E+05
0E+05
C1
C2
C3
C4
Clusters

Supplementary figure 4. Representative genes of clusters I and II and analysis of closest bidirectional genes.

a. Screen shot for the ChIP-seq tracks on a representative gene of RCOR1 cluster I in Figure 3d.
b. Screen shot for the ChIP-seq tracks on a representative gene of RCOR1 cluster II in Figure 3d.
c. Analysis of the distance from the closest bidirectional transcript on the 4 clusters in Figure 3d. For the box plots, quartile values (25, 50, 75 percentiles) were used for bounds of box and centre, and the upper and the lower whiskers are the maximum and minimum values of the data that is within 1.5 times the interquartile range over the 75th percentile and under the 25th percentile, respectively. The interquartile range is the difference between the 75th and 25th percentiles.

This figure shows related data to Main Figures 3 and 4.

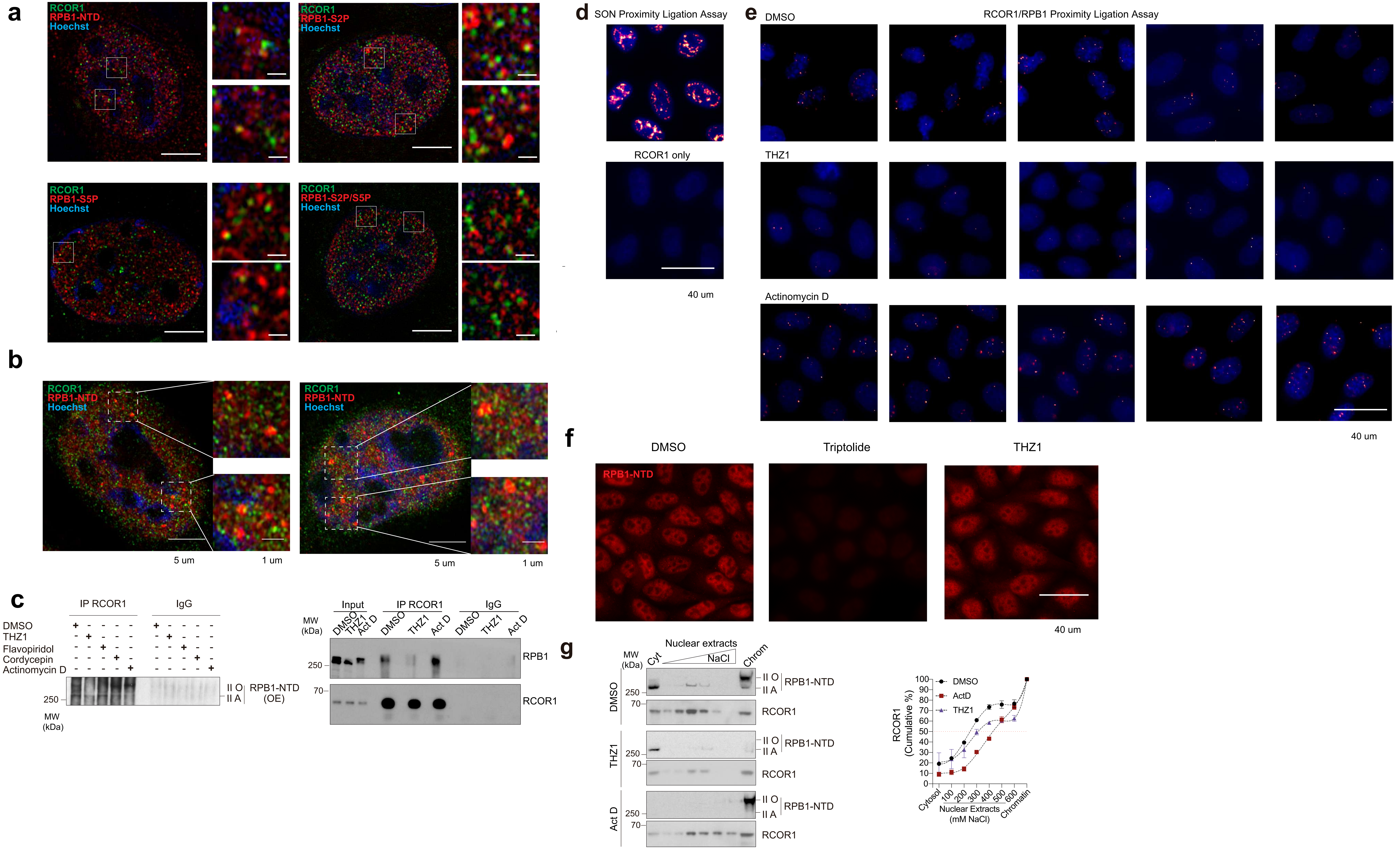
a
RCOR1
RPB1-NTD
Hoechst
RCOR1
RPB1-S2P
Hoechst
RCOR1
RPB1-S5P
Hoechst
RCOR1
RPB1-S2P/S5P
Hoechst
b
RCOR1
RPB1-NTD
Hoechst
5 um
1 um
c
IP RCOR1
IgG
DMSO
THZ1
Flavopiridol
Cordycepin
Actinomycin D
250
MW (kDa)
II O
II A
RPB1-NTD (OE)
Input
IP RCOR1
IgG
MW (kDa)
DMSO
THZ1
Act D
250
70
RPB1
RCOR1
d
SON Proximity Ligation Assay
RCOR1 only
40 um
e
RCOR1/RPB1 Proximity Ligation Assay
DMSO
THZ1
Actinomycin D
40 um
f
DMSO
Triptolide
THZ1
RPB1-NTD
40 um
g
Nuclear extracts
NaCl
MW (kDa)
Cyt
Chrom
DMSO
THZ1
Act D
250
70
II O
II A
RPB1-NTD
RCOR1
RCOR1 (Cumulative %)
DMSO
ActD
THZ1
Cytosol
Nuclear Extracts (mM NaCl)
Chromatin

Supplementary figure 5. RCOR1 and RPB1 colocalize and their subcellular distribution is impacted by transcription inhibitors.

a. HT22 cells were stained with double immunolabeling of RCOR1 (green) and different phosphorylated isoforms of RPB1 as shown in red. Right panels show magnified regions of original images. Scale bar is 5 µm. Images are representative of two independent experiments.
b. HeLa cells were stained with double immunolabeling of RCOR1 (green) and RPB1 (red). Right panels show magnified regions of original images. Images are representative of two independent experiments.
c. Left panel shows the overexposed (OE) western blot image obtained from Main Figure 5. Right panel shows a biological replicate of RCOR1 – RPB1 interaction under THZ1 and Actinomycin D treatments. Westerns are representative of three independent experiments.
d. Proximity ligation assays. Positive control consisted in staining the nuclear speckle protein SON with two different antibodies raised in mouse and rabbit. Negative control consisted in using RCOR1 antibody only.
e. Expanded number of representative fields of RCOR1/RPB1 proximity ligation assay. Scale bar is 40 µm. Proximity ligation assays were performed in three independent experiments.
f. RPB1 immunofluorescence in HT22 cells showing that THZ1 treatment does not change RPB1 levels. Triptolide was tested as a control of another transcription initiation inhibitor that induces RPB1 degradation. Images are representative of two independent experiments.
g. Western blot analyses of RPB1 and RCOR1 profiles on sequential salt-gradient extractions when THZ1 or Actinomycin D were used to inhibit

elongation. Cumulative protein levels of RCOR1 on each fraction, expressed as percent of the total detected levels. Panels are representative of three (DMSO and THZ1) and two (Act D) experiments

This figure shows related data to Main Figure 5.

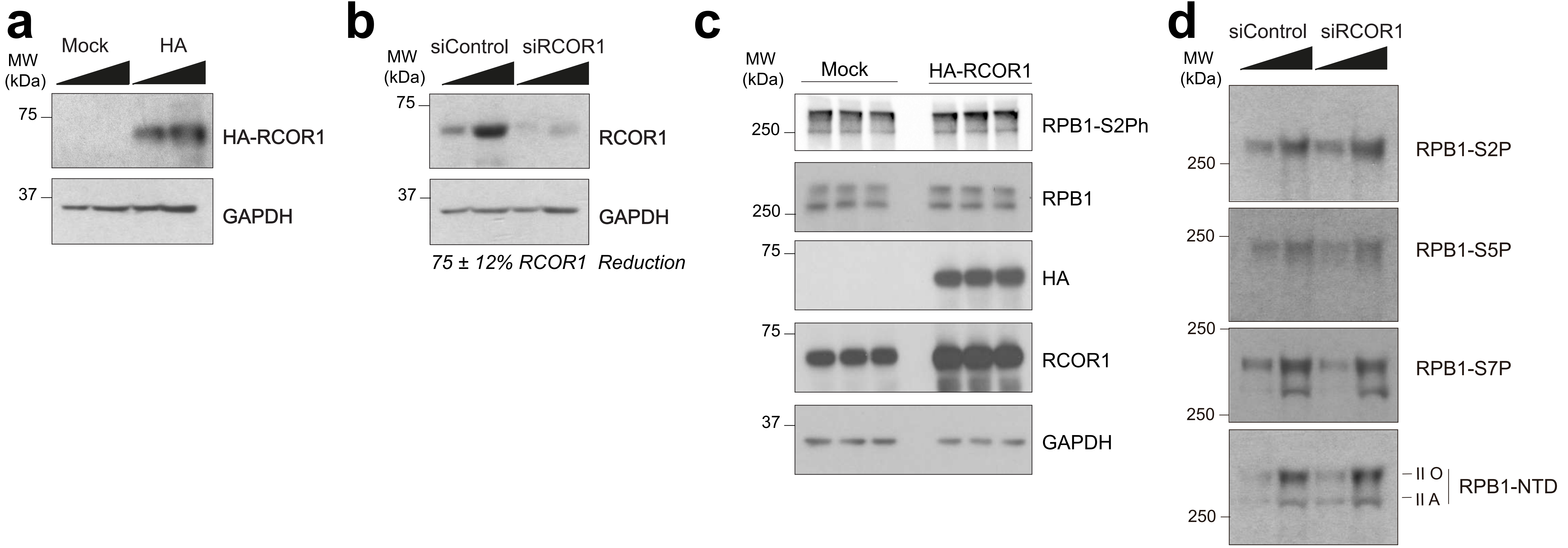
a
Mock
HA
MW (kDa)
75
37
HA-RCOR1
GAPDH
b
siControl
siRCOR1
MW (kDa)
75
37
RCOR1
GAPDH
75 ± 12% RCOR1 Reduction
c
MW (kDa)
Mock
HA-RCOR1
250
250
75
75
37
RPB1-S2Ph
RPB1
HA
RCOR1
GAPDH
d
siControl
siRCOR1
MW (kDa)
250
250
250
250
250
RPB1-S2P
RPB1-S5P
RPB1-S7P
II O
II A
RPB1-NTD

Supplementary figure 6. Experimental controls for RCOR1 overexpression and post-transcriptional silencing and RPB1-CTD phosphorylation.

a. Western blot analysis of HA-RCOR1 overexpression. GAPDH was assayed as a loading control. Panel is representative of two independent experiments.
b. Western blot analysis of RCOR1 knock down efficiency. GAPDH was assayed as loading control. Average is indicative of 3 biological replicates.
c. Western blot analysis of RPB1 S2 phosphorylation under HA-RCOR overexpression in HeLa cells. Three different biological replicates were included per condition.
d. Western blot analysis of RPB1 phosphorylations under RCOR1 knock down. This experiment was performed once.

This figure shows related data to Main Figure 6.

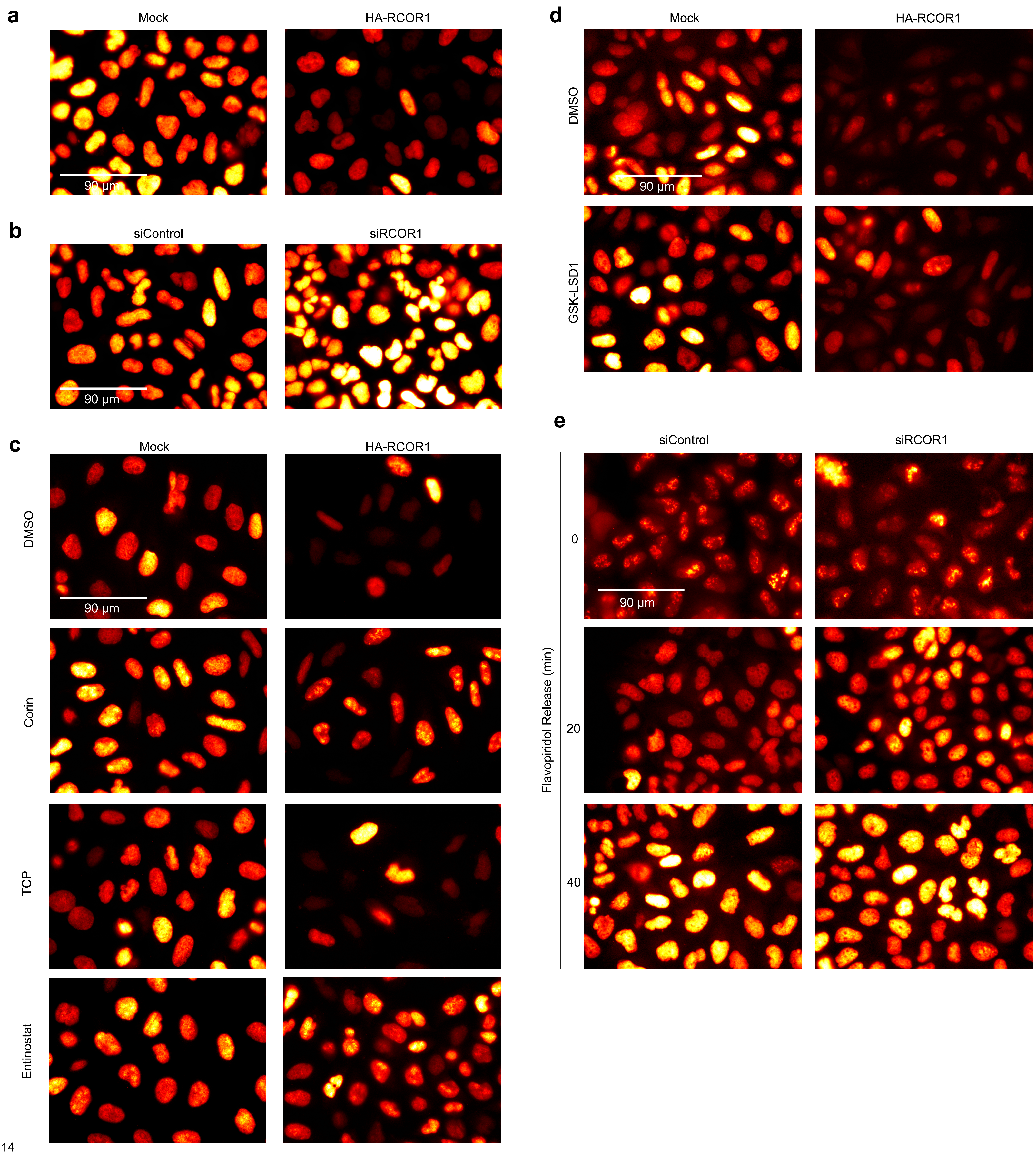

a
Mock
HA-RCOR1
90 µm
b
siControl
siRCOR1
90 µm
c
Mock
HA-RCOR1
DMSO
90 µm
Corin
TCP
Entinostat
d
Mock
HA-RCOR1
DMSO
90 µm
GSK-LSD1
e
siControl
siRCOR1
Flavopiridol Release (min)
0
90 µm
20
40

Supplementary figure 7. EU-incorporation images under all the conditions mentioned in Main Figure 6.

EU-incorporation experiments showing wider, zoomed-out fields of representative images of experiments conducted on HeLa cells. (a) RCOR1 overexpression. (b) RCOR1 knock down. (c) RCOR1 overexpression with treatments with Corin, TCP or Entinostat. (d) RCOR1 overexpression with treatments with GSK-LSD1. (e) RCOR1 knock down and Flavopiridol release.

Images are representative of three independent experiments.

This figure shows related data to Main Figure 6.

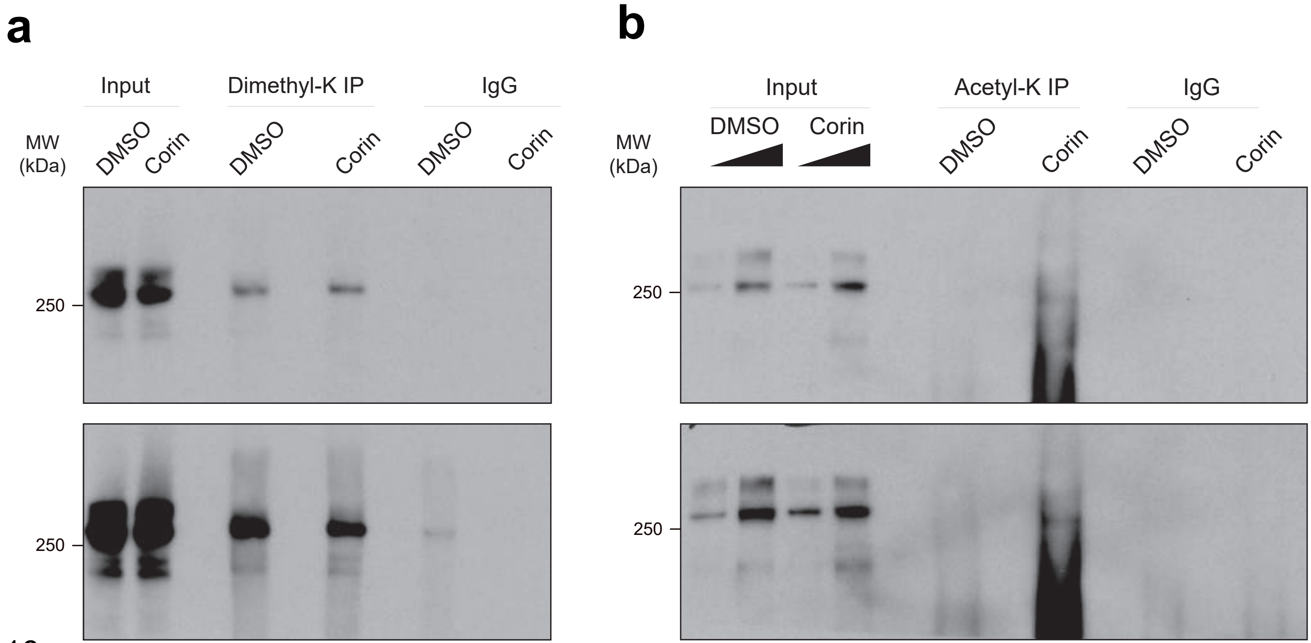
a
Input
Dimethyl-K IP
IgG
MW (kDa)
DMSO
Corin
DMSO
Corin
DMSO
Corin
250
250
b
Input
Acetyl-K IP
IgG
DMSO
Corin
MW (kDa)
DMSO
Corin
DMSO
Corin
250
250

Supplementary figure 8. Full scale western blot images for IP experiments on Main Figure 7.

Original western blot images for experiments studying RPB1 lysine dimethylation (a) and acetylation (b) under Corin treatment of HT22 cells. Blots are representative of two independent experiments.

This figure shows related data to Main Figure 7.

Supplementary Table 1. Metagenomic information – including BigWig file names and GEO Accession Numbers used in this study – obtained from publicly available sources (ENCODE project).

Target	BigWig File Name	GEO Accession Number
RCOR1	wgEncodeSydhTfbsK562Corestsc30189IggrabSig.bigWig	GSM935439
RCOR1 (Antibody #2)	wgEncodeSydhTfbsK562Corestab24166IggrabSig.bigWig	GSM935385
P300	wgEncodeSydhTfbsK562P300IggrabSig.bigWig	GSM935401
LSD1	wgEncodeBroadHistoneK562Lsd1Sig.bigWig	GSM1003570
HDAC1	wgEncodeBroadHistoneK562Hdac1sc6298StdSig.bigWig	GSM1003448
POL2	wgEncodeSydhTfbsK562Pol2StdSig.bigWig	GSM935358
H3K4me3	wgEncodeSydhHistoneK562H3k4me3bUcdSig.bigWig	GSM788087
H3K9ac	wgEncodeSydhHistoneK562H3k9acbUcdSig.bigWig	GSM788082
H3K27ac	wgEncodeBroadHistoneK562H3k27acStdSig.bigWig	GSM733656
H3K27me3	wgEncodeSydhHistoneK562bH3k27me3bUcdSig.bigWig	GSM788088
H3K9me3	wgEncodeBroadHistoneK562H3k9me3StdSig.bigWig	GSM733776
CHD4	wgEncodeBroadHistoneK562Chd4mi2Sig.bigWig	GSM1003510
SIN3A	wgEncode_K562_Sin3A_fcOverCtrl_ENCFF699SNH.bigWig	GSM2424155

EZH2	wgEncodeBroadHistoneK562Ezh239875StdSig.bigWig	GSM1003576
SUZ12	wgEncodeBroadHistoneK562Suz12051317Sig.bigWig	GSM1003545

- RNA-seq in K562 cells was obtained from the BigWig File "wgEncodeRegTxnCaltechRnaSeqK562R2x75Il200SigPooled" with accession number #GSM958729
