# Supplementary material for: Unveiling RCOR1 as a rheostat at transcriptionally permissive chromatin
Source: Nat Commun. 2022 Mar 23;13:1550. doi: 10.1038/s41467-022-29261-0 (PMC8943175; doi:10.1038/s41467-022-29261-0)
Supplement: Supplementary file 3 — Description of additional Supplementary File [file 41467_2022_29261_MOESM3_ESM.pdf]

Description for additional Supplementary Data Files

Supplementary Data 1 : Gene ontology analyses for gene cluster I generated by k-means approach on RCOR1 and Histone Modification ChIP-seq datasets. Categories enriched more than twofold are shown. The data are related to Main Figure 4.

Supplementary Data 2 : Gene ontology analyses for gene cluster II generated by k-means approach on RCOR1 and Histone Modification ChIP-seq datasets. Categories enriched more than twofold are shown. The
