# Supplementary material for: Unveiling RCOR1 as a rheostat at transcriptionally permissive chromatin
Source: Nat Commun. 2022 Mar 23;13:1550. doi: 10.1038/s41467-022-29261-0 (PMC8943175; doi:10.1038/s41467-022-29261-0)
Supplement: Supplementary file 6 — Reporting Summary [file 41467_2022_29261_MOESM6_ESM.pdf]

nature portfolio

Corresponding author(s): Joost Schymkowitz
Frederic Rousseau

Last updated by author(s): Jan 17, 2022

Reporting Summary

Nature Portfolio wishes to improve the reproducibility of the work that we publish. This form provides structure for consistency and transparency in reporting. For further information on Nature Portfolio policies, see our Editorial Policies and the Editorial Policy Checklist.

Statistics

For all statistical analyses, confirm that the following items are present in the figure legend, table legend, main text, or Methods section.

n/a	Confirmed	
☐	☒	The exact sample size (*n*) for each experimental group/condition, given as a discrete number and unit of measurement
☐	☒	A statement on whether measurements were taken from distinct samples or whether the same sample was measured repeatedly
☐	☒	The statistical test(s) used AND whether they are one- or two-sided<br>*Only common tests should be described solely by name; describe more complex techniques in the Methods section.*
☒	☐	A description of all covariates tested
☒	☐	A description of any assumptions or corrections, such as tests of normality and adjustment for multiple comparisons
☐	☒	A full description of the statistical parameters including central tendency (e.g. means) or other basic estimates (e.g. regression coefficient) AND variation (e.g. standard deviation) or associated estimates of uncertainty (e.g. confidence intervals)
☐	☒	For null hypothesis testing, the test statistic (e.g. *F*, *t*, *r*) with confidence intervals, effect sizes, degrees of freedom and *P* value noted<br>*Give P values as exact values whenever suitable.*
☒	☐	For Bayesian analysis, information on the choice of priors and Markov chain Monte Carlo settings
☒	☐	For hierarchical and complex designs, identification of the appropriate level for tests and full reporting of outcomes
☐	☒	Estimates of effect sizes (e.g. Cohen's *d*, Pearson's *r*), indicating how they were calculated

Our web collection on statistics for biologists contains articles on many of the points above.

Software and code

Policy information about availability of computer code

Data collection	Structural modeling and thermodynamic calculations were performed using FoldX 5. FTIR spectral acquisition and analysis was performed using the OPUS software (v8.5.29). ThT curves were monitored and analysed using software provided from BMG Labtech (OMEGA v5.11, MARS v3.33).
Data analysis	Umap and PCA was performed using appropriate R packages (umap, prcomp). High content screening analysis was performed using the Columbus Plus digital platform (PerkinElmer) and colocalisation analysis was performed using coloc2 in ImageJ. Data plotting was performed using Prism 9. Yasara (21.8.26) was used for structural visualisations. MST results were analysed using Nanotemper analysis software (v2.2.4).

For manuscripts utilizing custom algorithms or software that are central to the research but not yet described in published literature, software must be made available to editors and reviewers. We strongly encourage code deposition in a community repository (e.g. GitHub). See the Nature Portfolio guidelines for submitting code & software for further information.

Data

Policy information about availability of data

All manuscripts must include a data availability statement. This statement should provide the following information, where applicable:

- Accession codes, unique identifiers, or web links for publicly available datasets
- A description of any restrictions on data availability
- For clinical datasets or third party data, please ensure that the statement adheres to our policy

The data supporting the findings of this study are available from the corresponding authors upon reasonable request. The source data underlying Figs. 2, 5, 6b, 6c, 8c, 8d, 8e, 9b, 9d and 9e, Supplementary Fig. 3, Supplementary Fig. 4, and Supplementary Fig. 5c and 5d, Supplementary Fig. 6a and Supplementary Fig. 8 are provided in a Source Data file and in Supplementary Tables 1, 2 and 3. The dataset of APR core structures is provided in Supplementary Table 1. The protein

constructs used in the cellular assays are shown in Supplementary Table 2. All clinical cases are listed in Supplementary Table 3 with the main parameters, with the main clinical diagnosis, age and gender. Due to legislation and privacy protection any medical reports and files of the cases included in this study cannot be made available. Source data are provided with this paper.

Field-specific reporting

Please select the one below that is the best fit for your research. If you are not sure, read the appropriate sections before making your selection.

- [x] Life sciences
- [ ] Behavioural & social sciences
- [ ] Ecological, evolutionary & environmental sciences

For a reference copy of the document with all sections, see nature.com/documents/nr-reporting-summary-flat.pdf

Life sciences study design

All studies must disclose on these points even when the disclosure is negative.

Sample size	For the computational analysis, we included every single mutation for every steric zipper shown in Table 1 (n=10374). We generated and tested a peptide library of 83 peptides for the tau APR and 10 peptides for the ApoAI peptide. All possible peptides were selected for the tau dataset, whereas representative peptides were selected for the ApoAI set as described in the manuscript. All experiments were performed in three or more independent repeats in every case to ensure reproducibility. Experiments with patient derived material were performed in duplicates for three independent cases (n=6) due to sample availability restrictions.
Data exclusions	No data has been excluded in this study.
Replication	ThT fluorescence assays, transmission electron microscopy and FRAP measurements were performed in triplicates (n=3). Critical concentration analysis (n=4), dye-binding (n=30), FTIR (n=4), MST (n=3) sample sizes were reported. For cellular assays, each condition was tested in three independent wells (n=3). Pearson's correlation coefficients were calculated for (n=10) individual cells from 3 independent wells (n=3). For patient derived material, we used three individual cases, each of which was tested in duplicates (n=6). All attempts of replication were successful.
Randomization	Due to the in vitro nature of our study, no randomization was necessary for our biophysical and cellular studies.
Blinding	Blinding was not possible as the researchers were involved in the synthesis and in vitro screening of the peptide library. Similarly, for the cellular assays, the researchers needed to be fully aware of the samples involved to properly interpret the results.

Reporting for specific materials, systems and methods

We require information from authors about some types of materials, experimental systems and methods used in many studies. Here, indicate whether each material, system or method listed is relevant to your study. If you are not sure if a list item applies to your research, read the appropriate section before selecting a response.

Materials & experimental systems

n/a	Involved in the study	
[ ]	[x]	Antibodies
[ ]	[x]	Eukaryotic cell lines
[x]	[ ]	Palaeontology and archaeology
[x]	[ ]	Animals and other organisms
[ ]	[x]	Human research participants
[x]	[ ]	Clinical data
[x]	[ ]	Dual use research of concern

Methods

n/a	Involved in the study	
[x]	[ ]	ChIP-seq
[x]	[ ]	Flow cytometry
[x]	[ ]	MRI-based neuroimaging

Antibodies

Antibodies used	Alexa Fluor 647 goat anti-rabbit antibody (ThermoFisher, A-21245), HA-tag (C29F4) Rabbit mAb (Cell Signalling, #3724)
Validation	All antibodies used in this study have been tested by the manufacturer and have been cited by other authors as described in the manufacturer page.<br><br>Alexa Fluor 647 goat anti-rabbit antibody (ThermoFisher, A-21245) was validated for use in immunohistochemistry (1-10ug/mL recommended dilutions) by the manufacturer and other authors (10.7554/eLife.43747, 10.7554/eLife.22689) and for real-time super-resolution microscopy (https://dx.plos.org/10.1371/journal.pone.0062918).<br><br>HA-tag (C29F4) Rabbit mAb (Cell Signalling, #3724) has been validated by the supplier with SimpleChIP Enzymatic Chromatin IP Kit (Magnetic Beads) #9003 in HEK293T and with flow cytometric analysis.

Eukaryotic cell lines

Policy information about cell lines

Cell line source(s)	Tau RD P301S FRET Biosensor (ATCC CRL-3275).
Authentication	Tau RD P301S FRET Biosensor cell line was authenticated by the supplier (ATCC). Seeding competency was tested in-house with a gradient concentration of recombinant tau seeds, and aggregate properties of the inclusions were validated with the FRAP experiments reported. No additional authentication was performed.
Mycoplasma contamination	Cells tested negative for mycoplasma
Commonly misidentified lines (See ICLAC register)	None

Human research participants

Policy information about studies involving human research participants

Population characteristics	Autopsy brain samples from 3 individuals, a female, age 71 and 2 males, ages 71 and 87, diagnosed with AD. Population characteristics are reported in detail in Supplementary Table 3.
Recruitment	Brain tissue from autopsy cases was received from UZ/KU Leuven Biobank. For all samples an informed consent sheet has been signed either by the patient during life or by the next in kin after death.
Ethics oversight	The research involving human tissue samples was carried out under ethical approval by the UZ Leuven ethical committee (Leuven/Belgium; File-No. S63759). An informed consent for autopsy and scientific use of autopsy tissue with clinical information was granted.

Note that full information on the approval of the study protocol must also be provided in the manuscript.
