# Supplementary material for: Unveiling RCOR1 as a rheostat at transcriptionally permissive chromatin
Source: Nat Commun. 2022 Mar 23;13:1550. doi: 10.1038/s41467-022-29261-0 (PMC8943175; doi:10.1038/s41467-022-29261-0)
Supplement: Supplementary file 7 — Source Data [file 41467_2022_29261_MOESM7_ESM.zip › Rivera et al 2022 - NCOMMS - Source Data File .pdf]

SOURCE DATA FILE

Unveiling RCOR1 as a rheostat at transcriptionally permissive chromatin

Carlos Rivera [1,2,3,‡], Hun-Goo Lee [2,3,‡], Anna Lappala[2,3], Danni Wang[2,3], Verónica Noches[1], Montserrat Olivares-Costa[1], Marcela Sjöberg-Herrera[1], Jeannie T. Lee [2,3,#,*] & María Estela Andrés [1,#,*]

[1] Department of Cellular and Molecular Biology, Faculty of Biological Sciences, Pontificia Universidad Católica de Chile. Santiago, 8331150, Chile. [2] Department of Molecular Biology, Massachusetts General Hospital. Boston, MA 02114, USA. [3] Department of Genetics. The Blavatnik Institute, Harvard Medical School. Boston, MA 02114, USA.[‡] These authors contributed equally to this work. [#] These authors agreed to be considered as corresponding authors. [*] To whom correspondence should be addressed: María Estela Andrés, Ph.D. E-mail: mandres@bio.puc.cl. Jeannie T. Lee, MD, Ph.D. E-mail: lee@molbio.mgh.harvard.edu

DESCRIPTION : This file contains uncropped, unprocessed images of all the western blot images used in this study. Red-dashed rectangular sections are highlighted to show the section that was cropped for use in the final figures. All additional data not present here are available upon request.

FIGURE 1 C - SUBCELLULAR FRACTIONATION

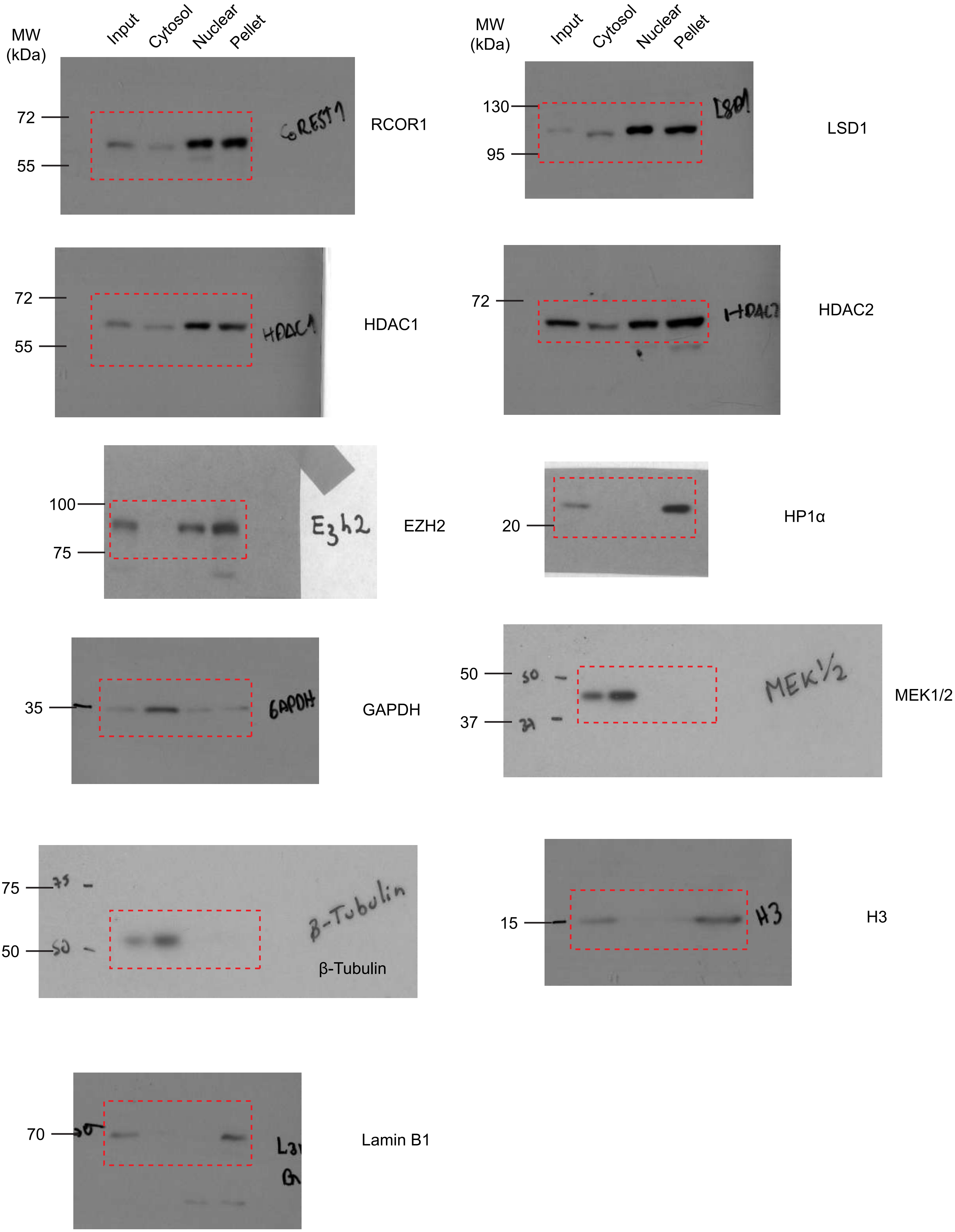

FIGURE 1 D - NaCl extraction gradient

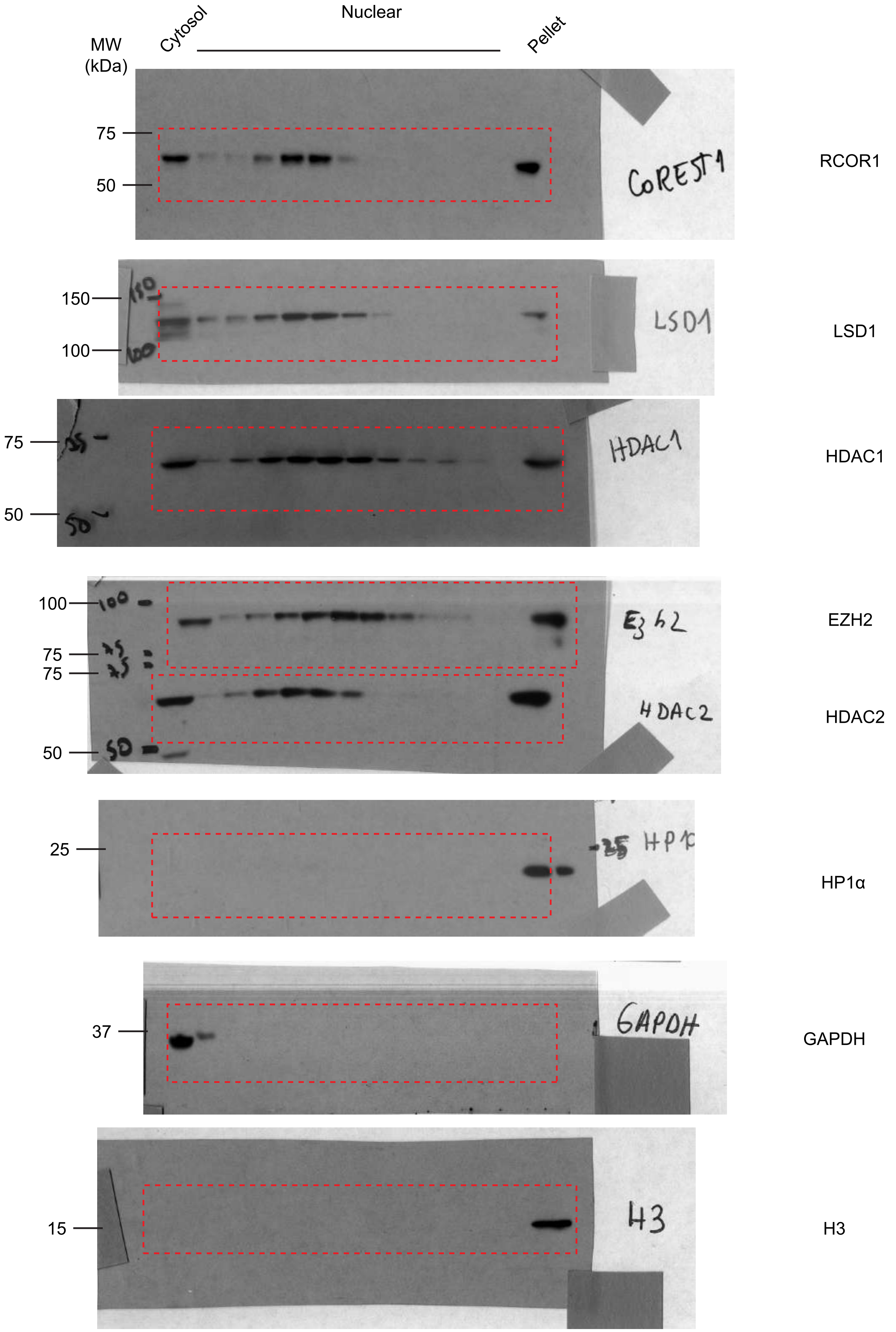

FIGURE 1 E - RCOR1 IP specificity controls

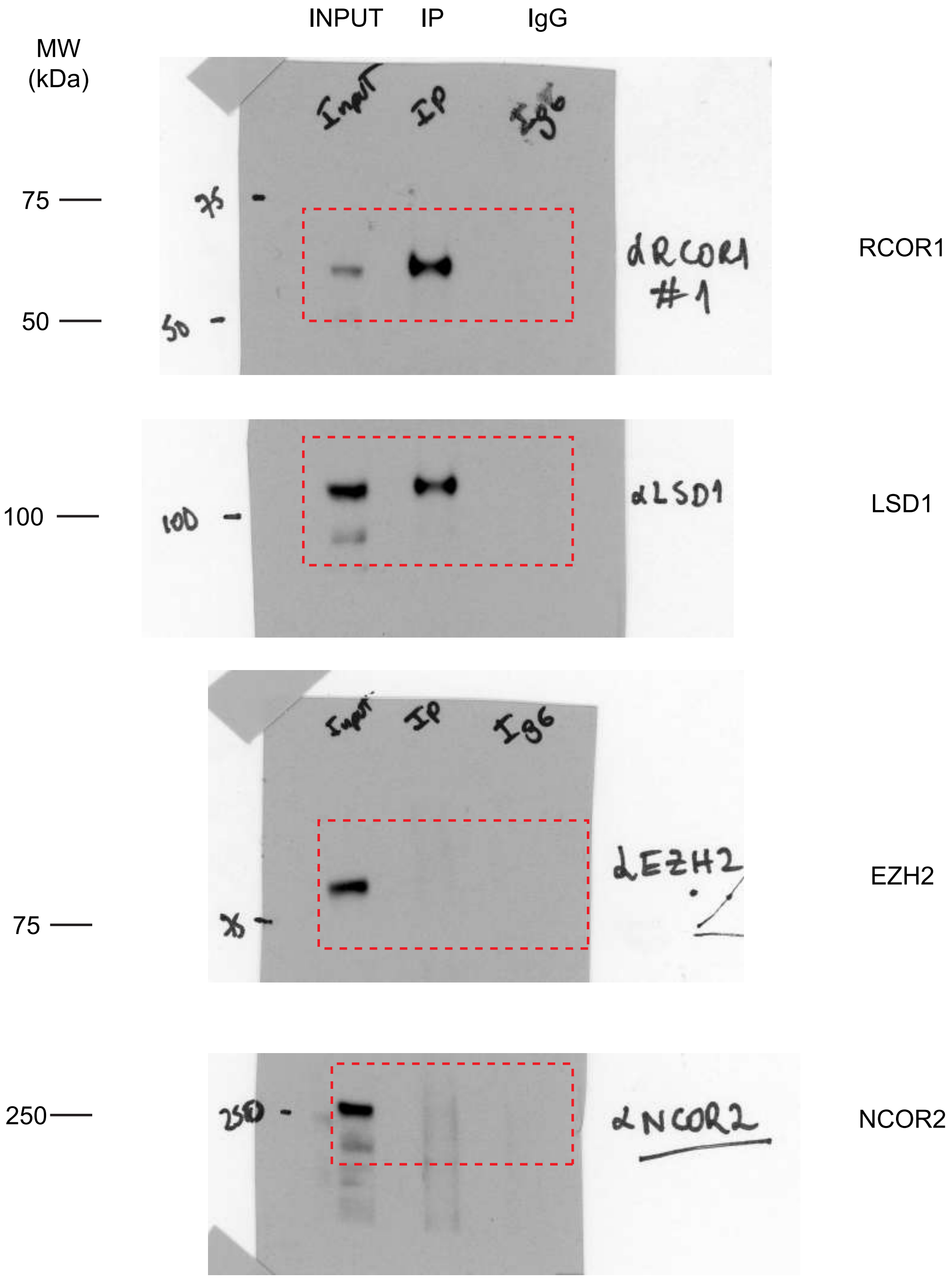

FIGURE 1 F - RCOR1 IP in different subcellular fractions

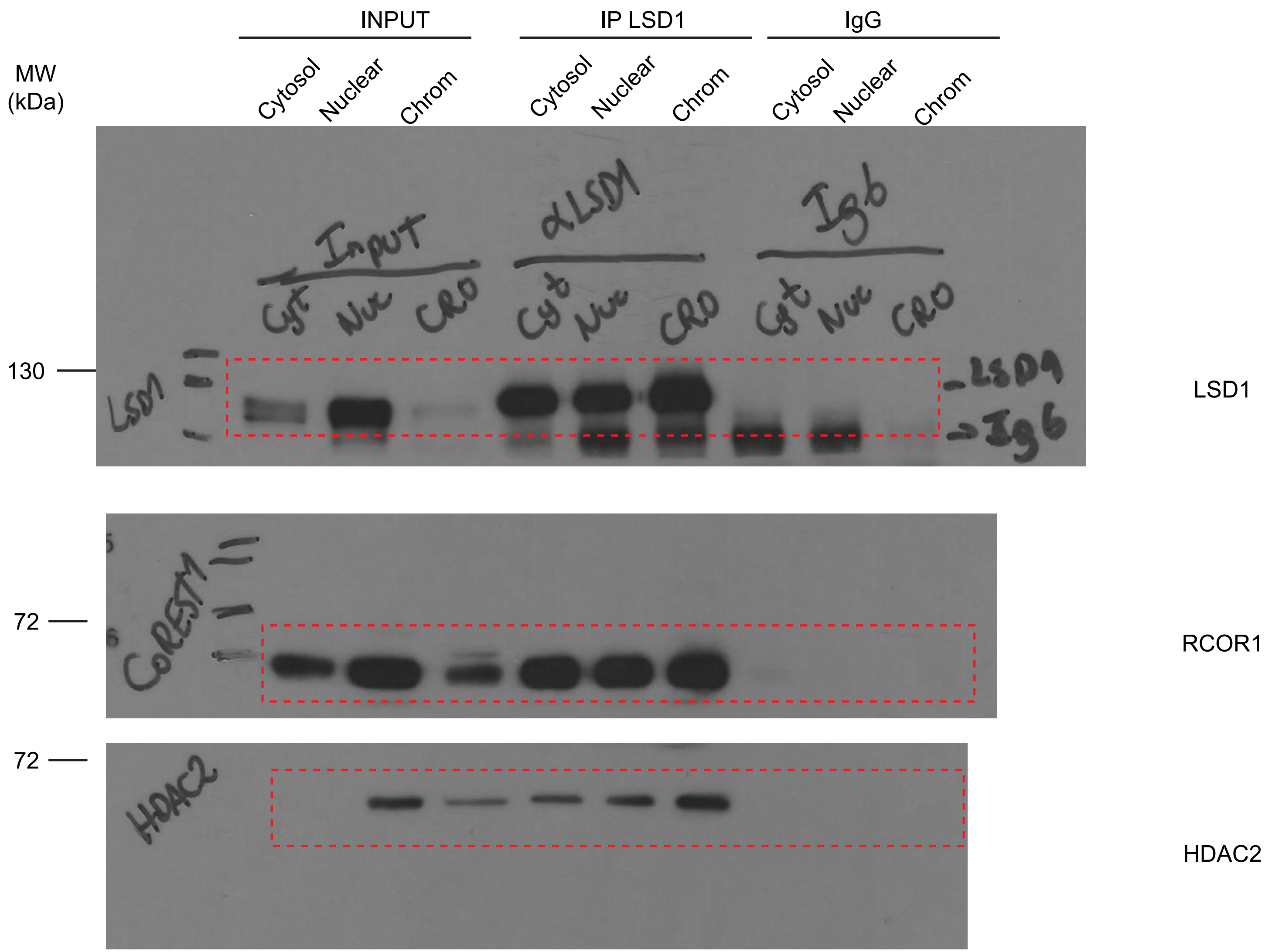

FIGURE 2B - RCOR1 IP FROM MNased-Chromatin samples

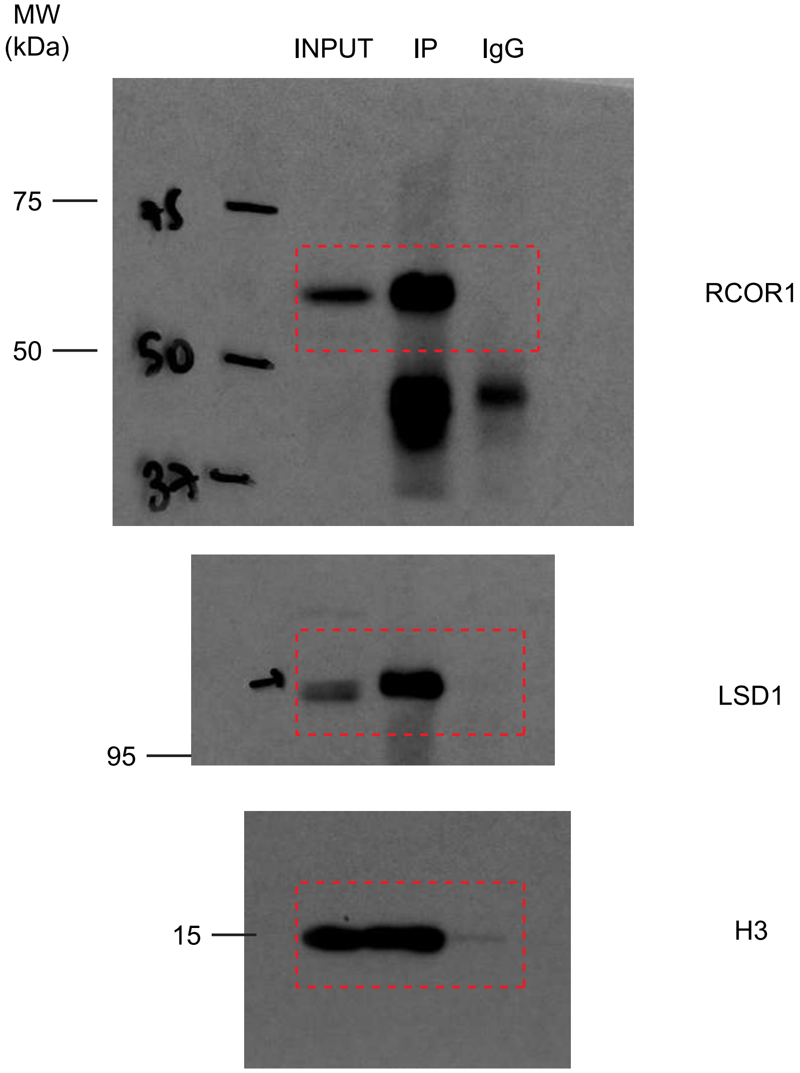

FIGURE 2C - RCOR1 IP and Histone Modifications CoIPs

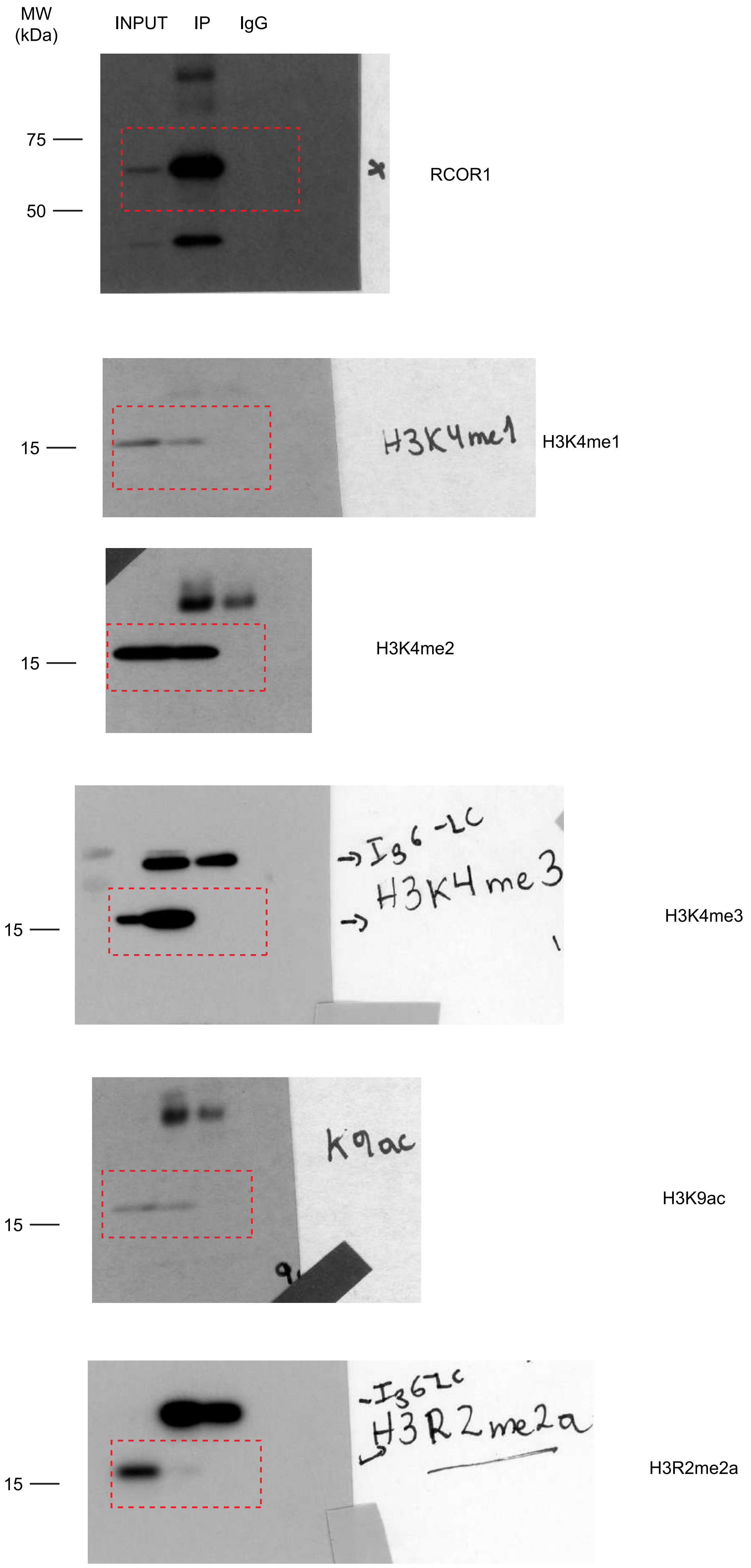

FIGURE 2E - Sucrose gradients

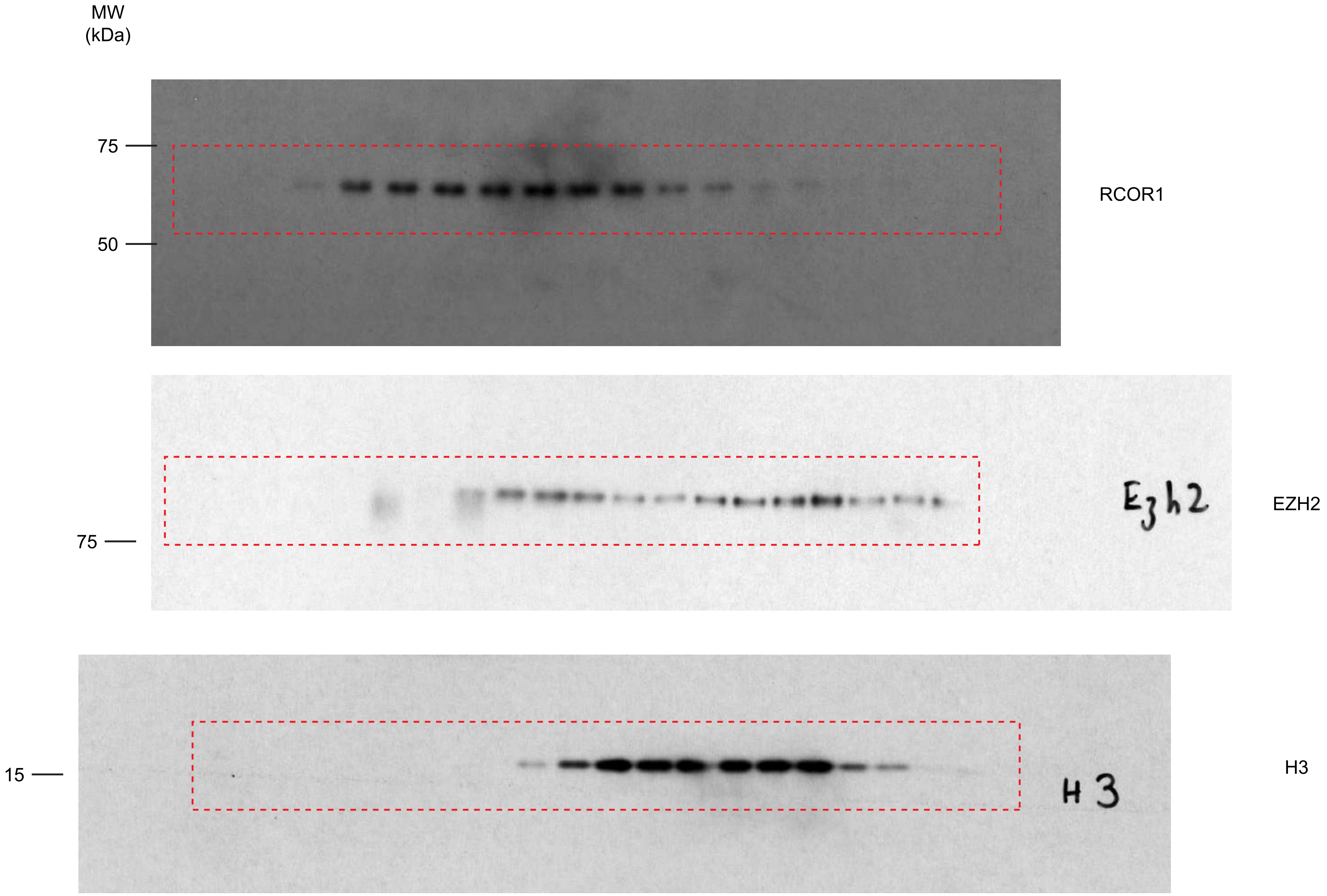

FIGURE 5A - RCOR1 IP and RPB1 CoIP

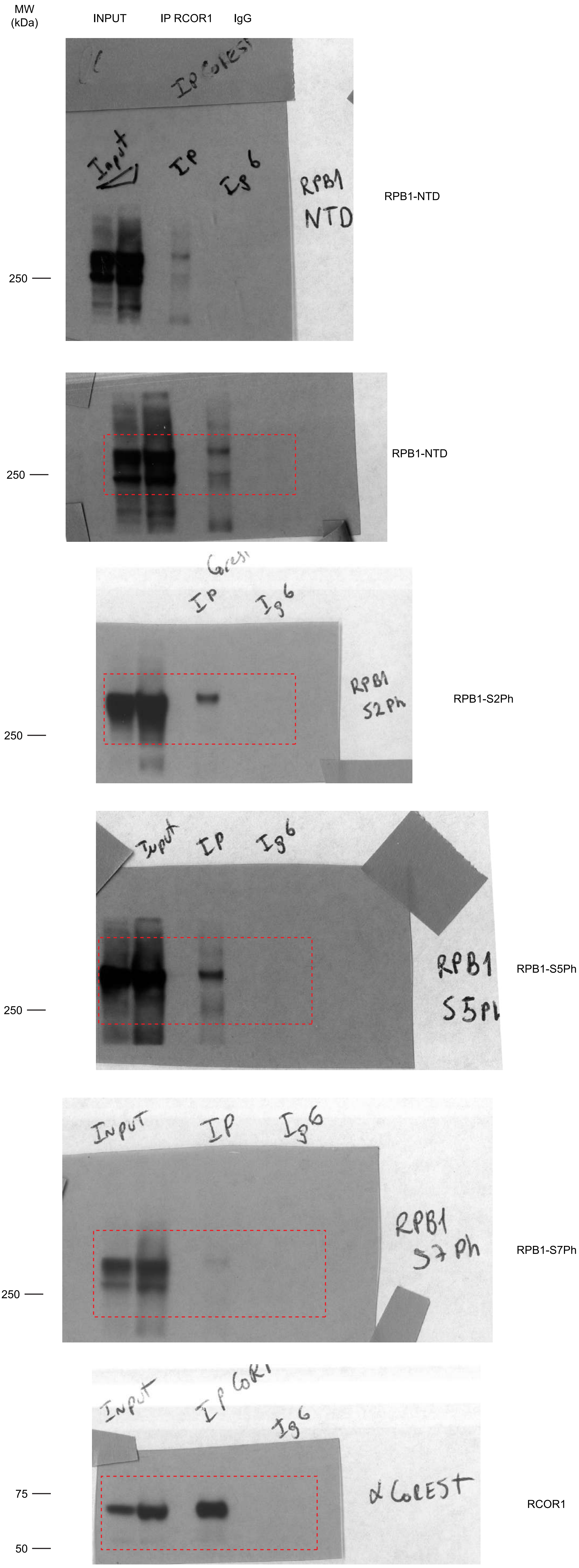

FIGURE 5B - LSD1/HDAC1 IP and RPB1 CoIP

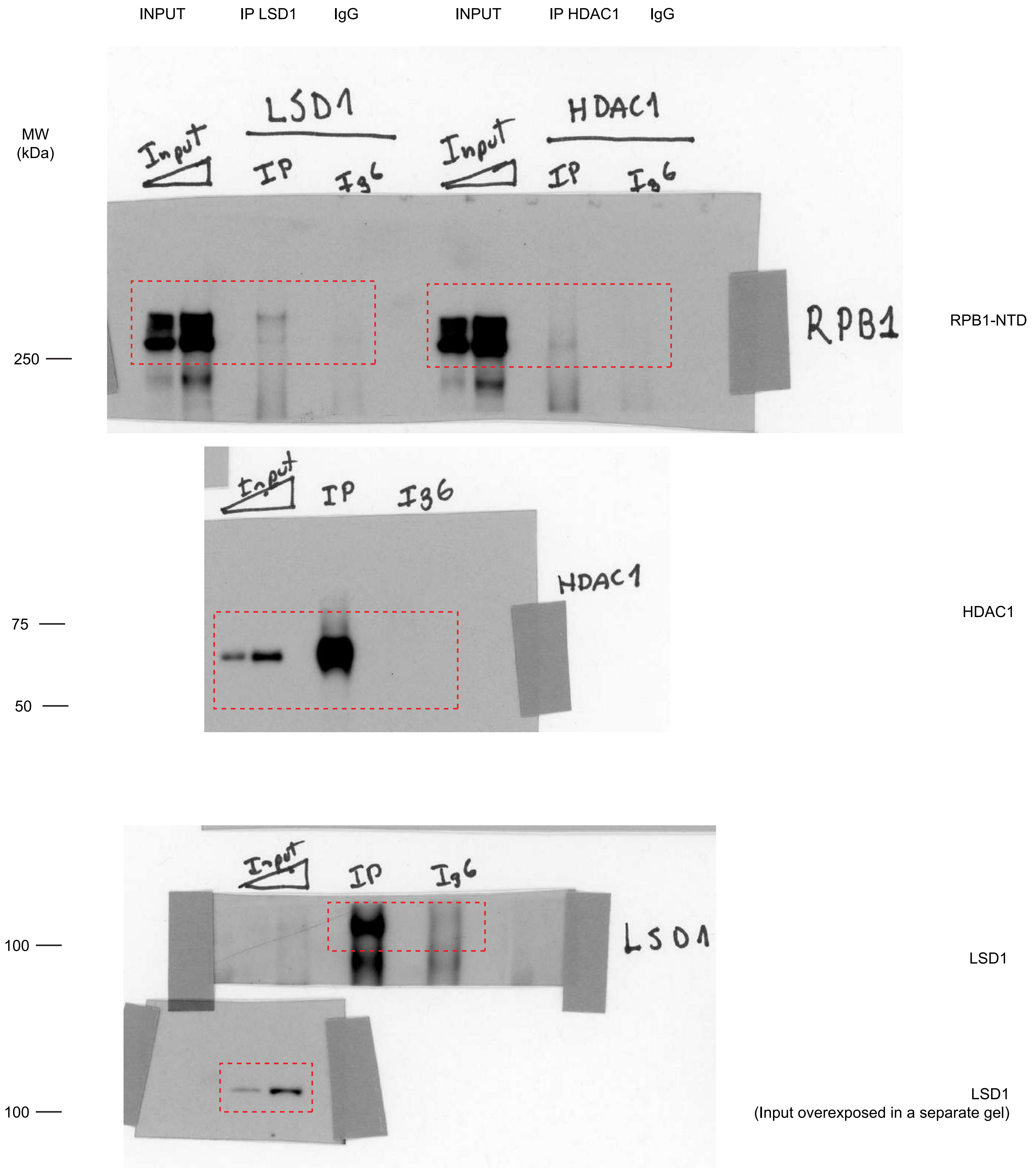

FIGURE 5C- CoIP RCOR1 - RPB1 in MNased Chromatin Extracts

		1 µg antibody		2 µg antibody	
	INPUT	IP	IgG	IP	IgG

MW (kDa)

250 —

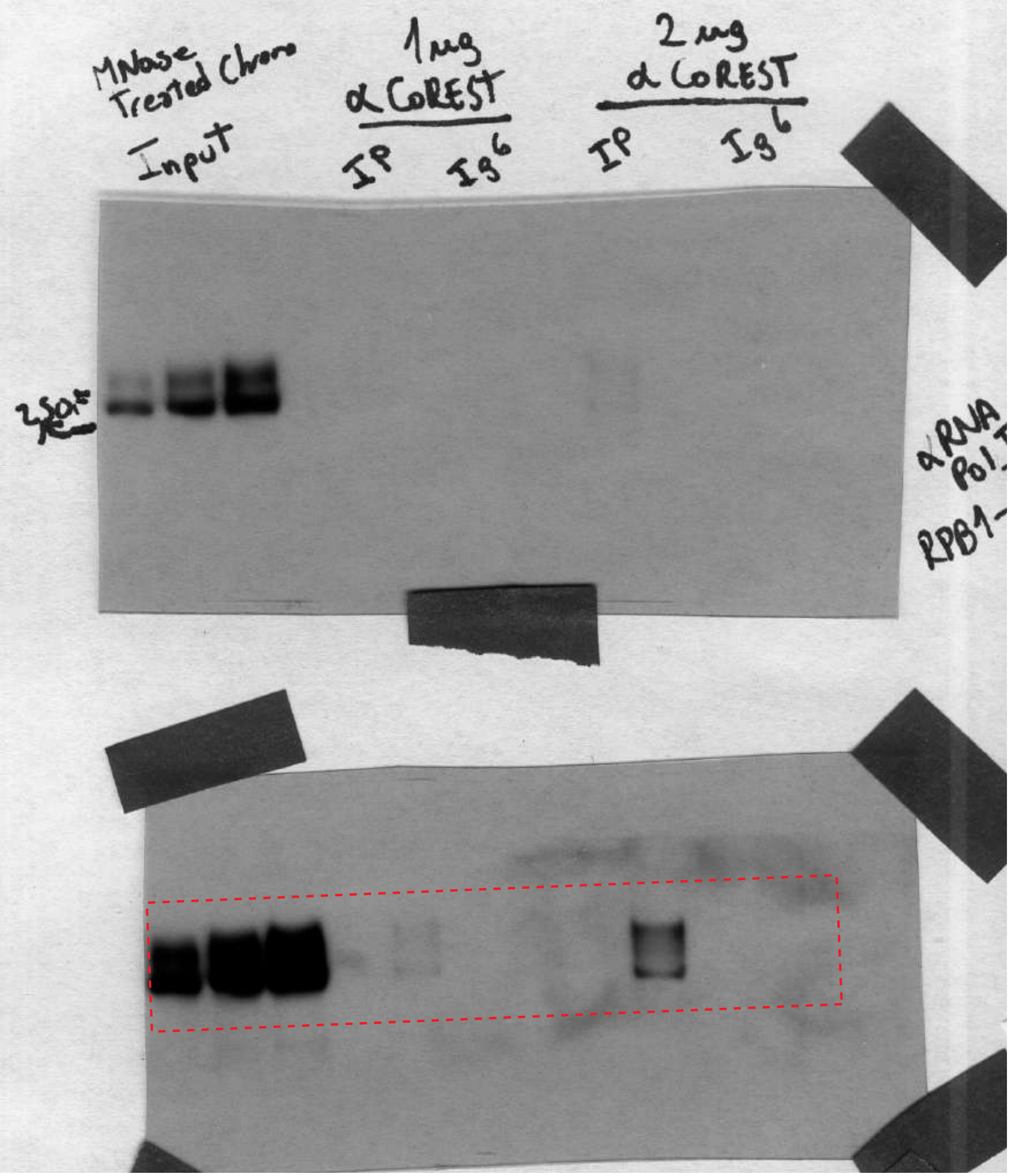

RPB1-NTD

250 —

RPB1-NTD

FIGURE 5D- CoIP HA-RCOR1 - RPB1

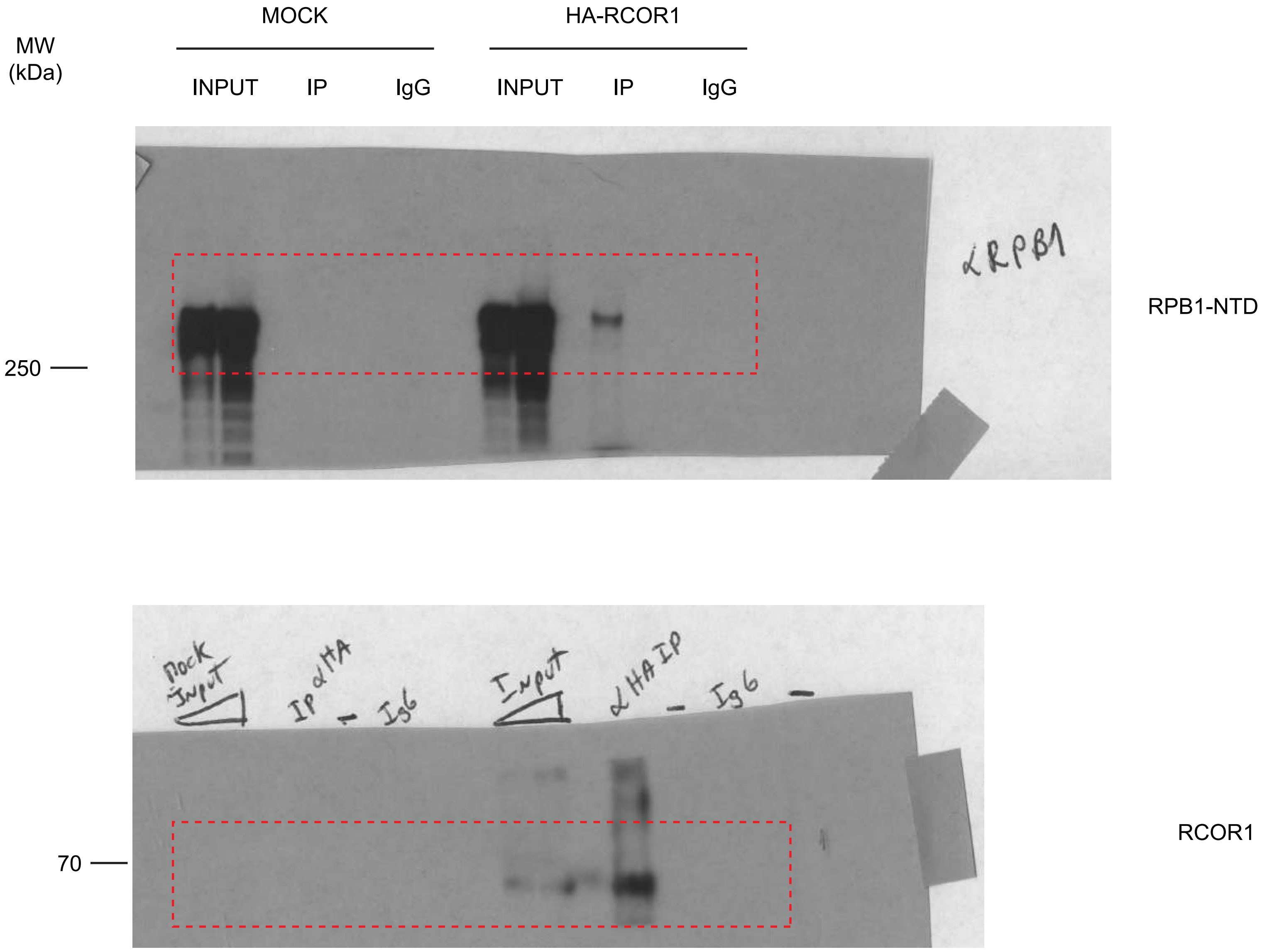

FIGURE 5F - CoIP RCOR1 - RPB1 with transcription inhibitors

INPUT — 1 2 3 4 5 | IP RCOR1 — 1 2 3 4 5 | IgG — 1 2 3 4 5

MW (kDa)

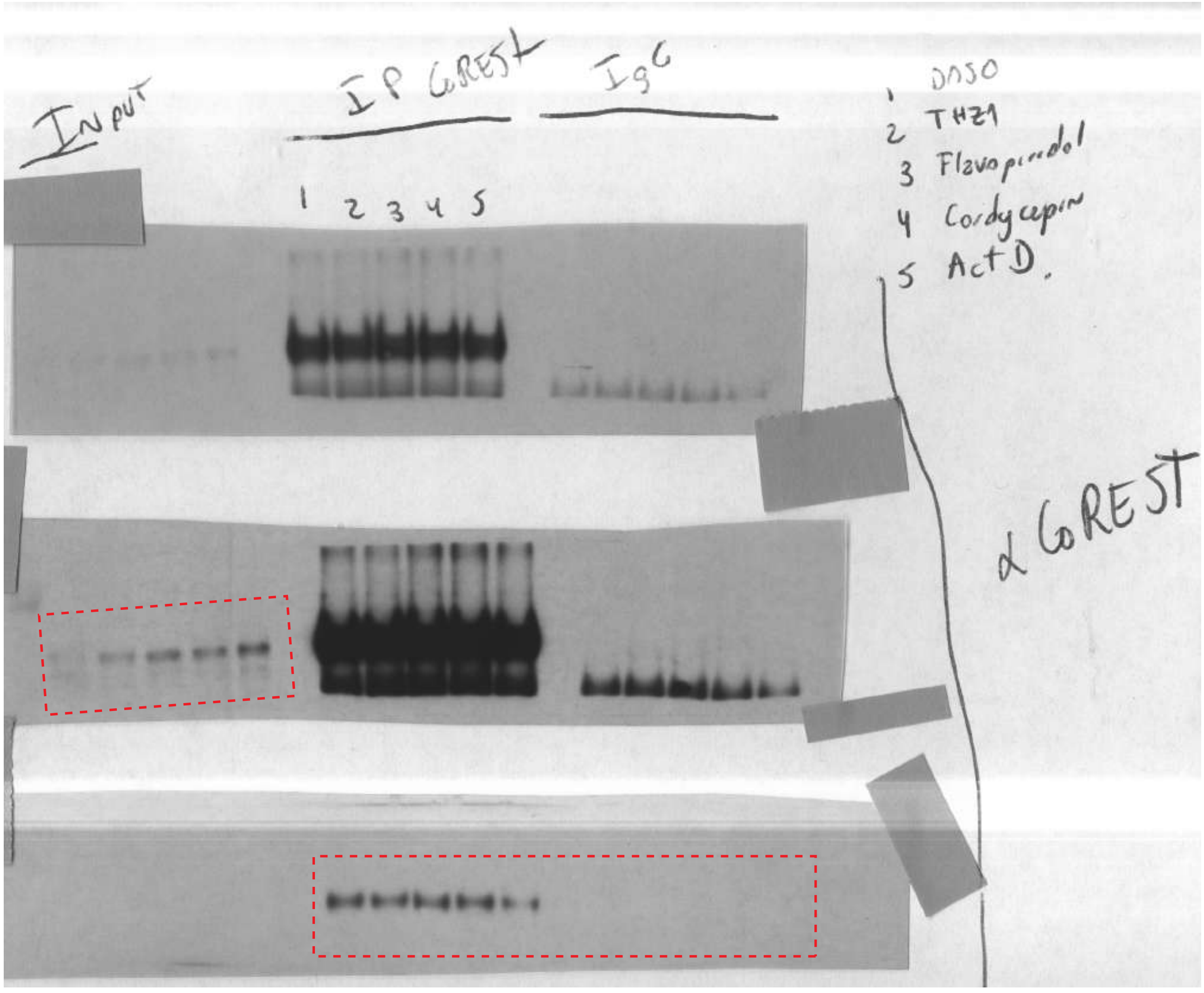

70 —

70 —

1: DMSO
2: THZ1
3: Flavopiridol
4: Cordycepin
5: Actinomycin D

RCOR1

RCOR1

INPUT — 1 2 3 4 5 | IP RCOR1 — 1 2 3 4 5 | IgG — 1 2 3 4 5

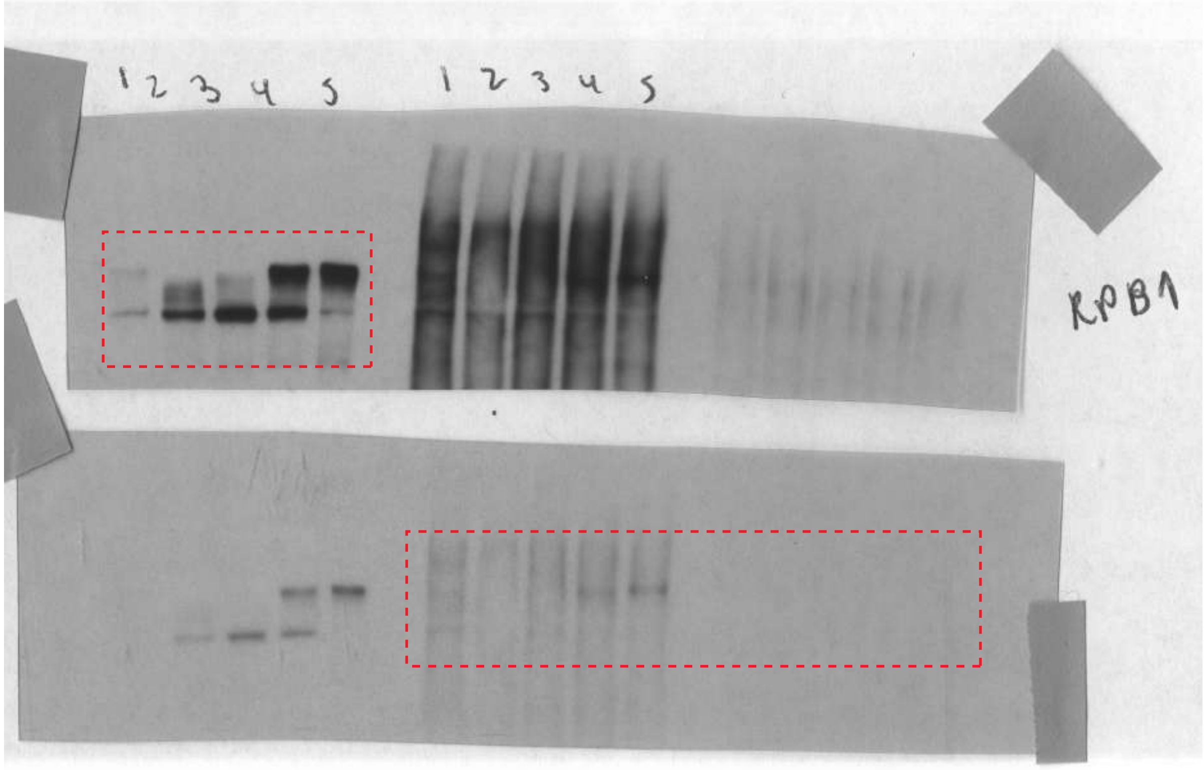

250—

250—

RPB1-NTD

RPB1-NTD

FIGURE 7B - Histone modifications after Corin treatment

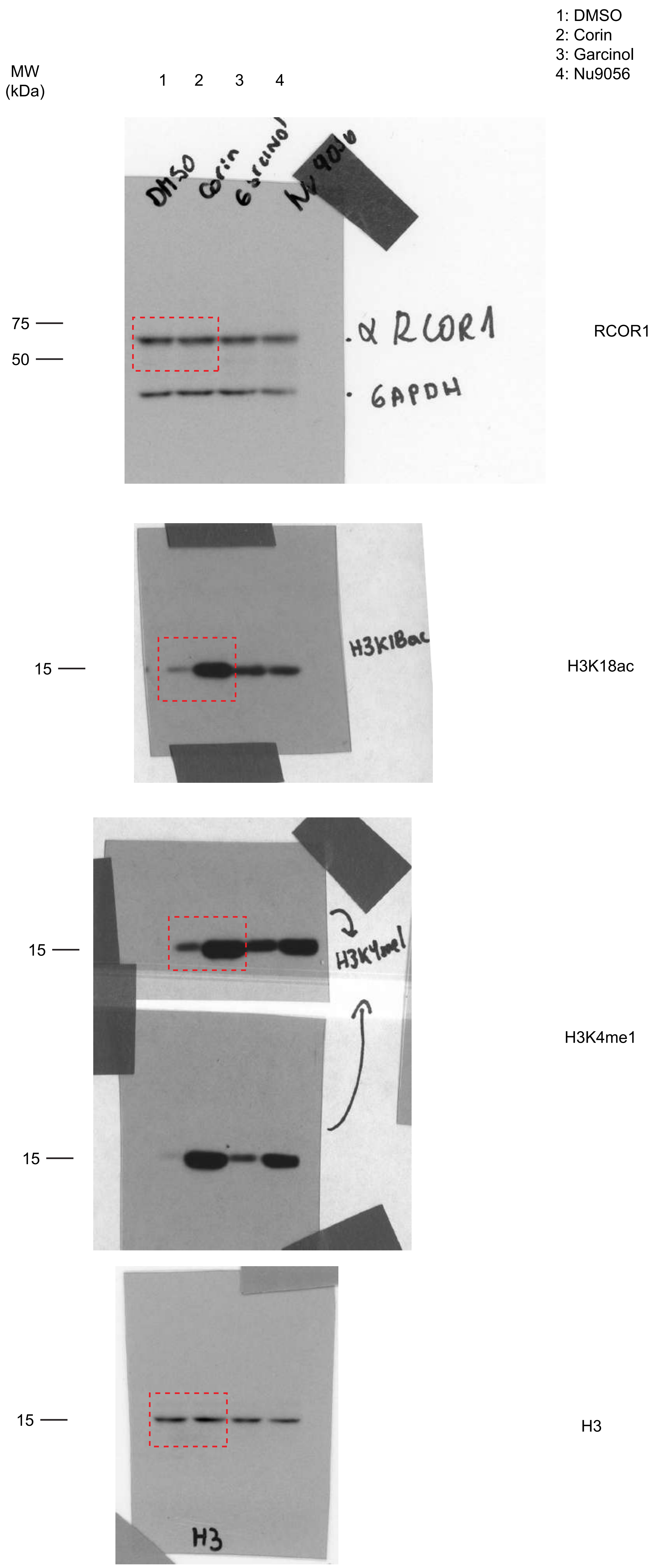

FIGURE 7D - CoIP RCOR1-RPB1 under Corin treatment

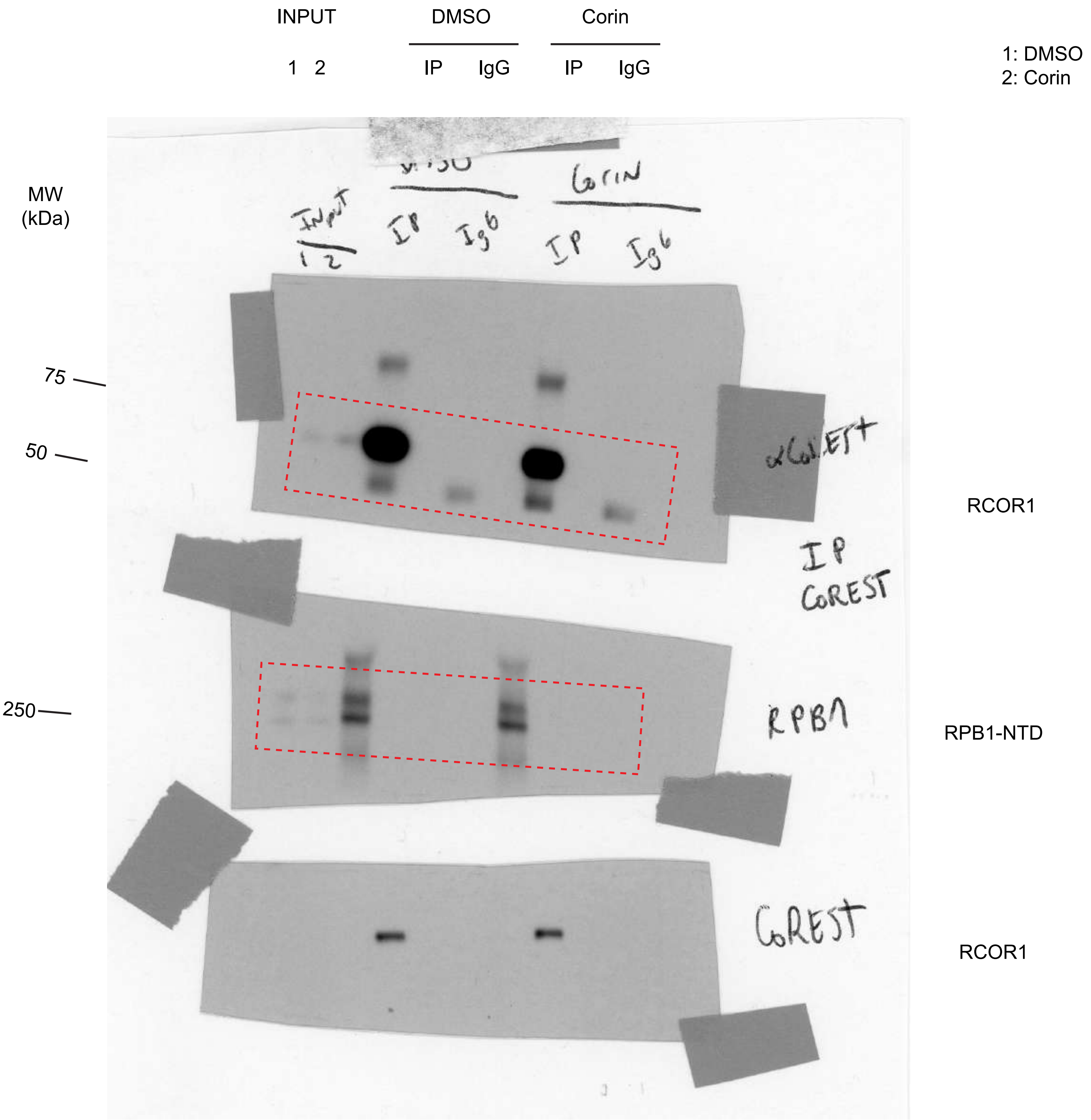

FIGURE 7F - Dimethyl-K IP + RPB1 Western blot under Corin treatment

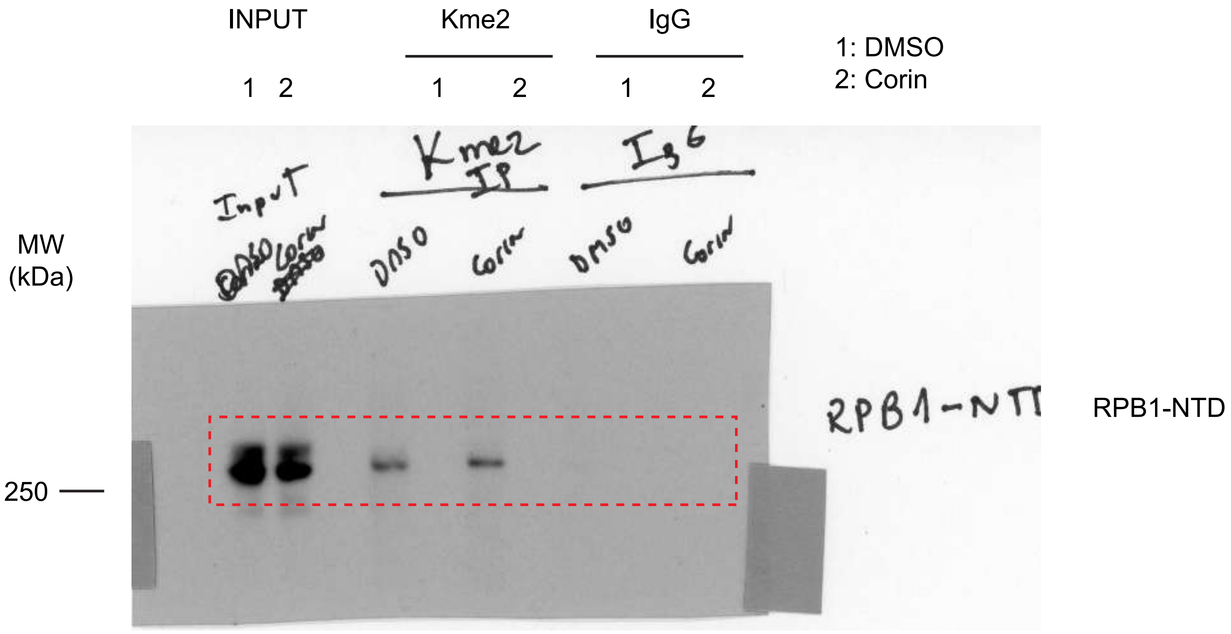

FIGURE 7G - Acetyl-K IP + RPB1 Western blot under Corin treatment

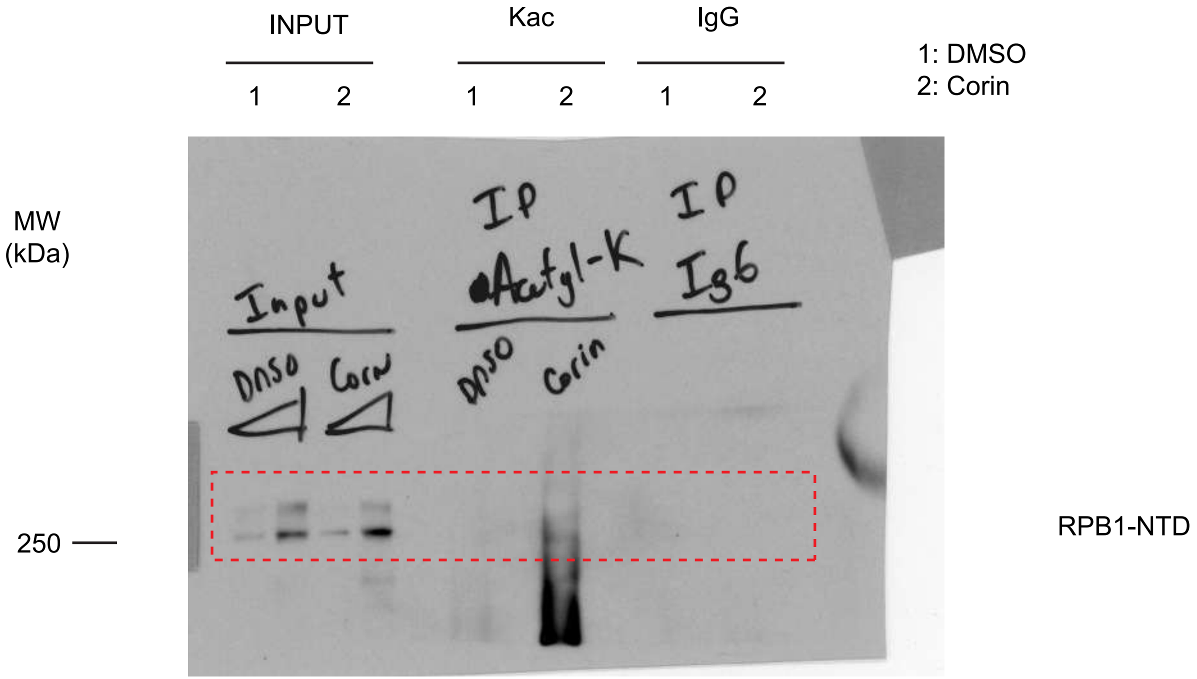

FIGURE 7H - RPB1 IP + RPB1-K7ac Western blot

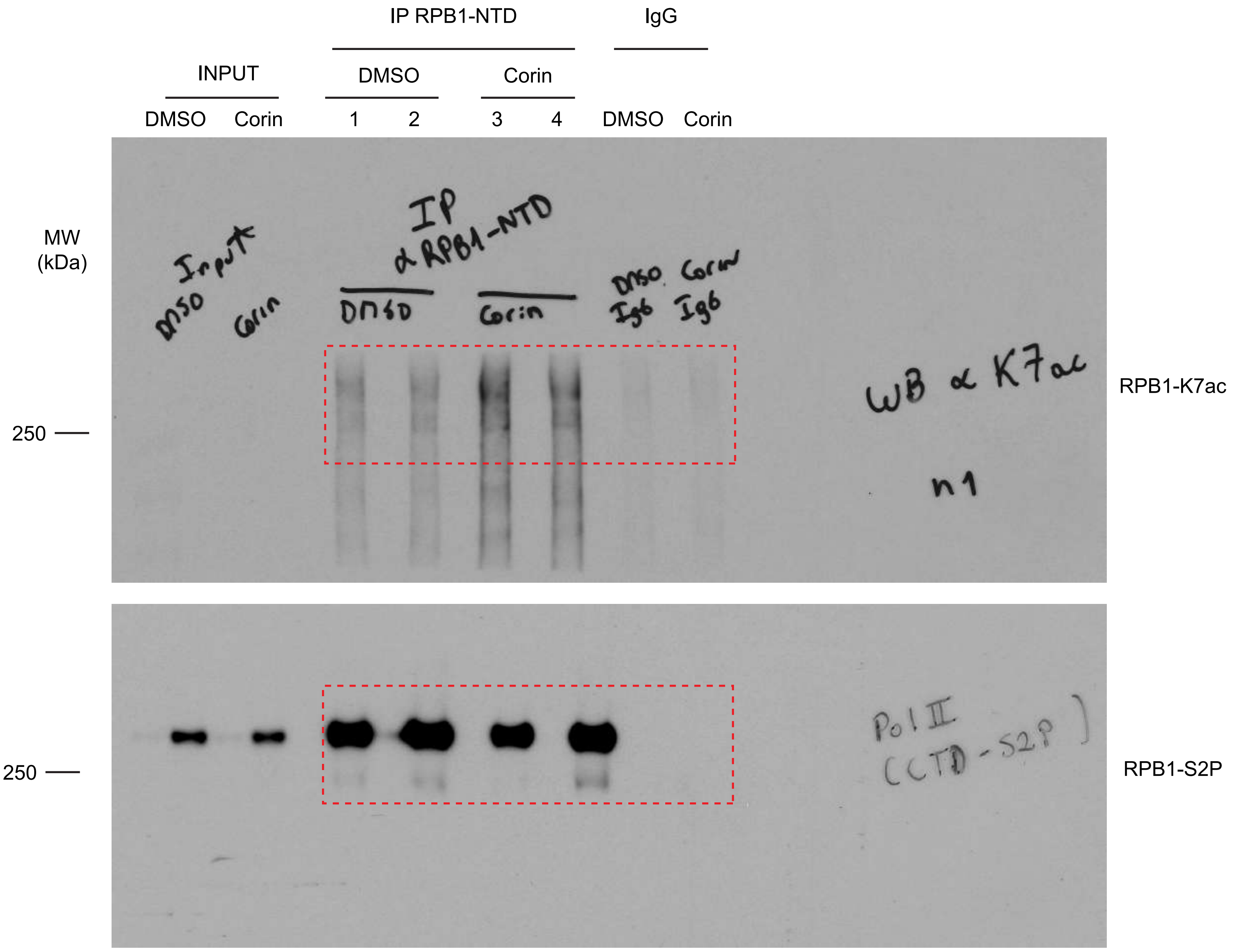

FIGURE 7I - RPB1-K7ac Subcellular distribution DMSO vs Corin

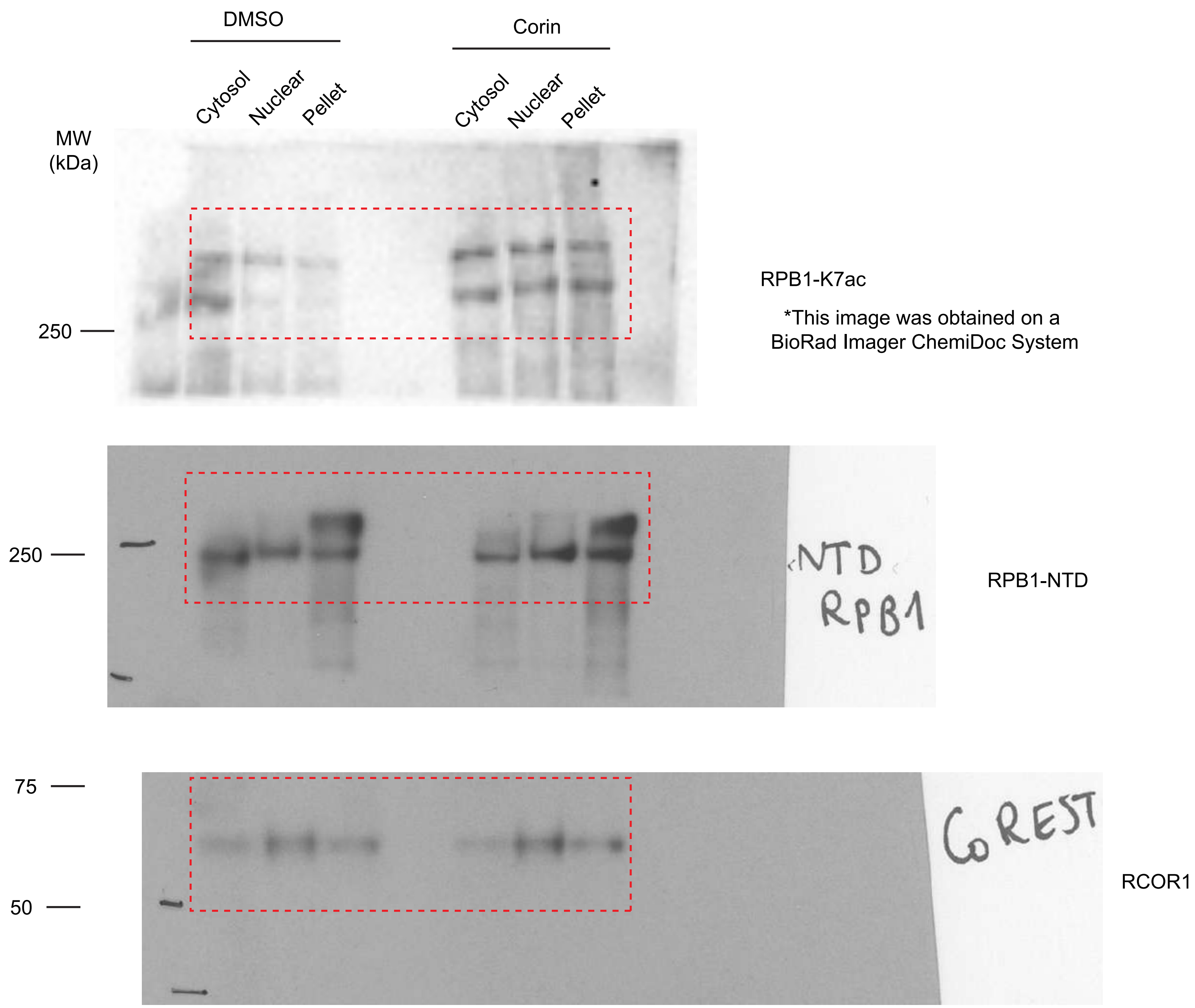

FIGURE S5C - CoIP RCOR1 RPB1 under THZ1 or ActD treatments

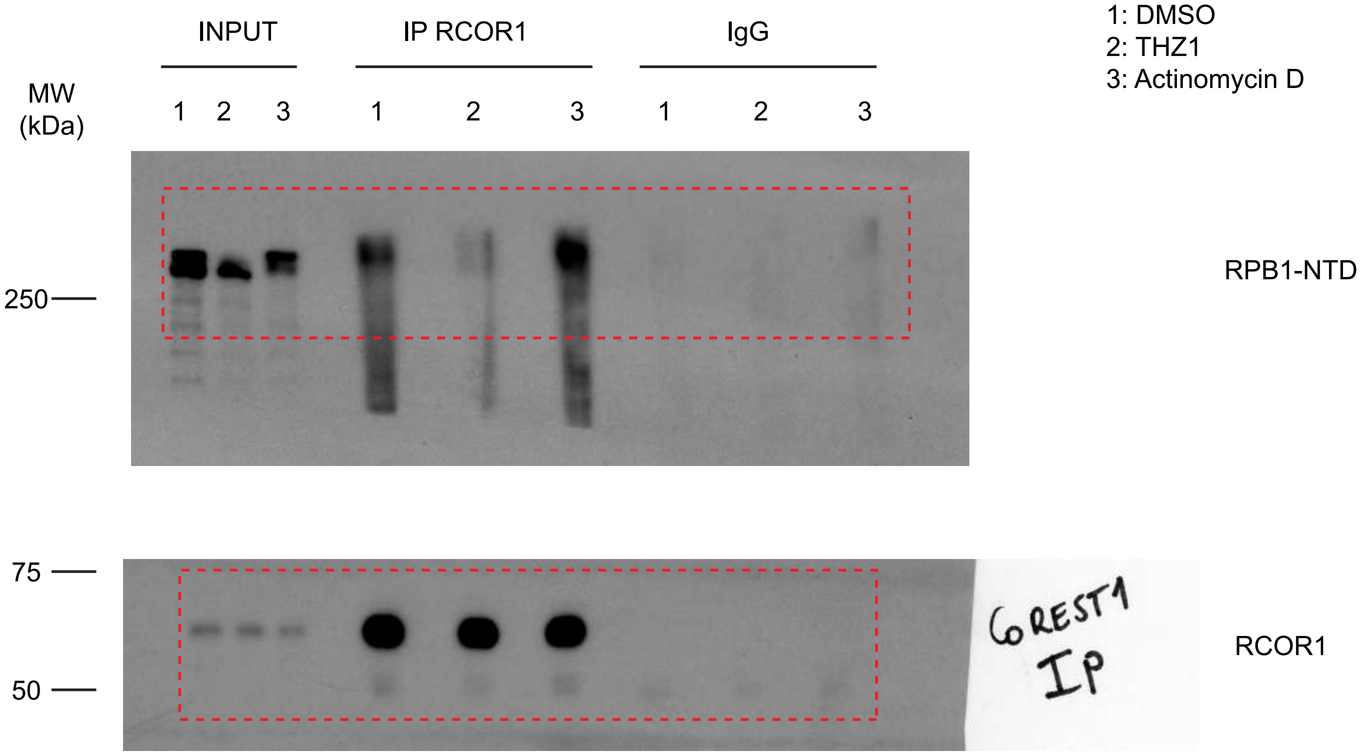

FIGURE S5G - Subcellular distribution of RCOR1 and RPB1 under transcriptional inhibition

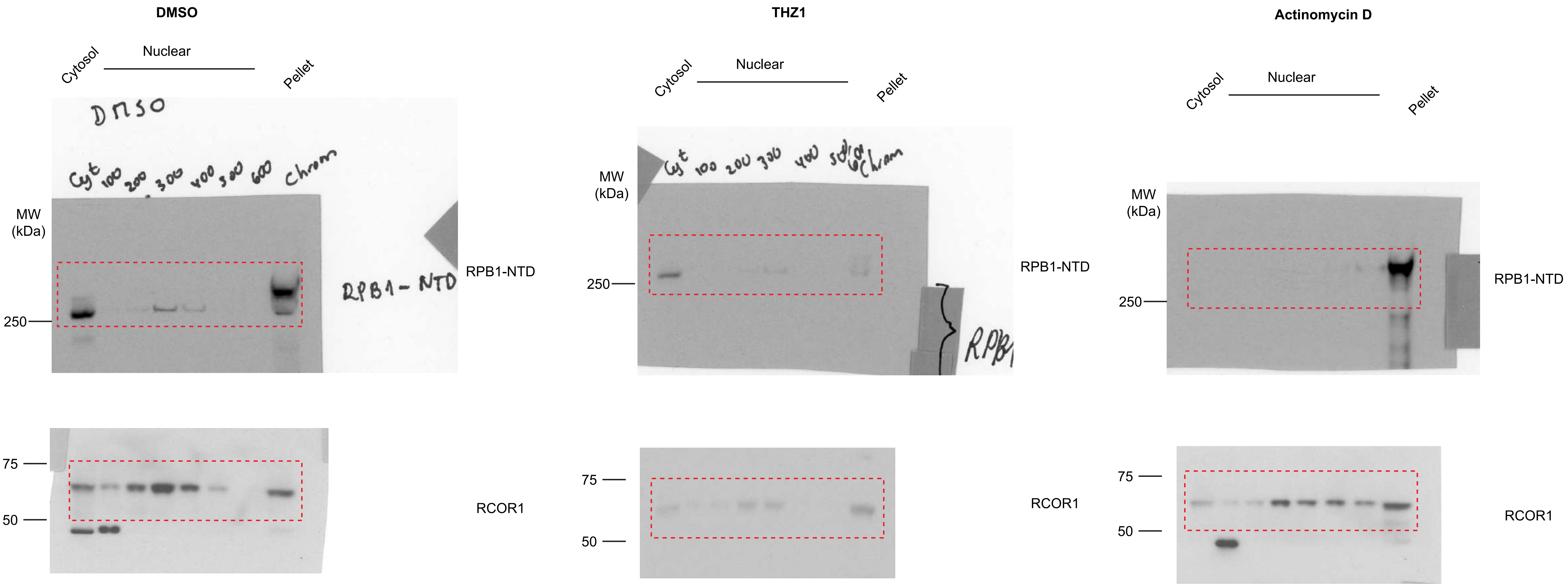

FIGURE S6A - HA-RCOR1 Overexpression

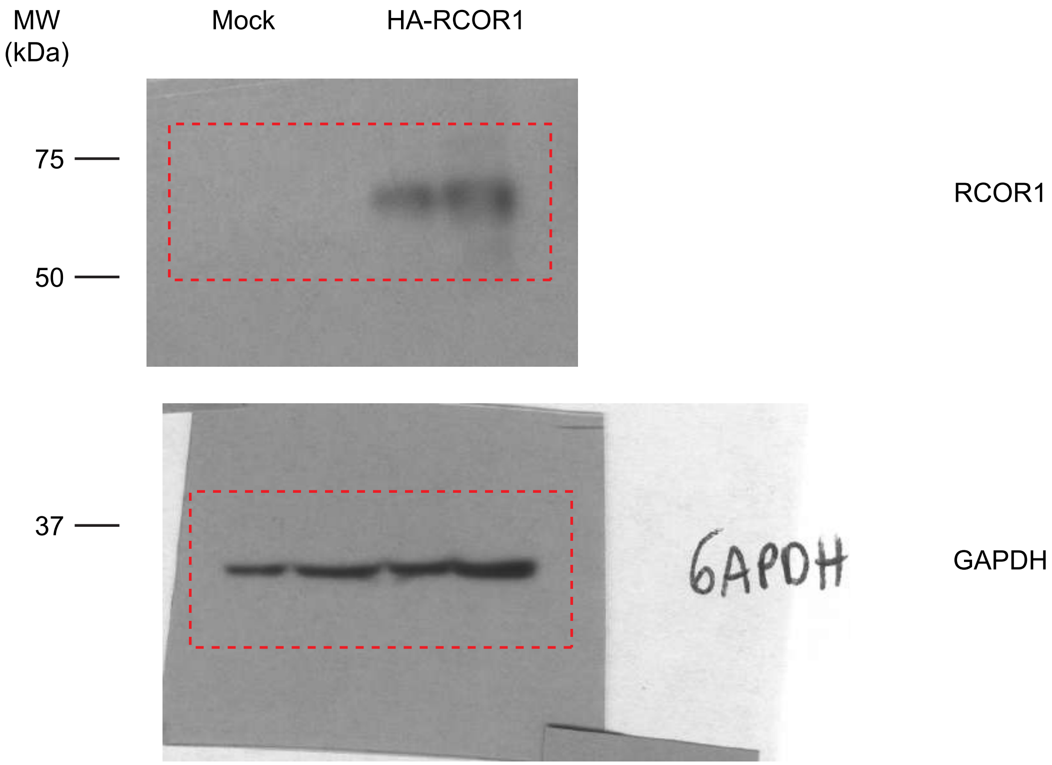

FIGURE S6B - RCOR1 knock down

MW (kDa)

siControl siRCOR1

75

50

37

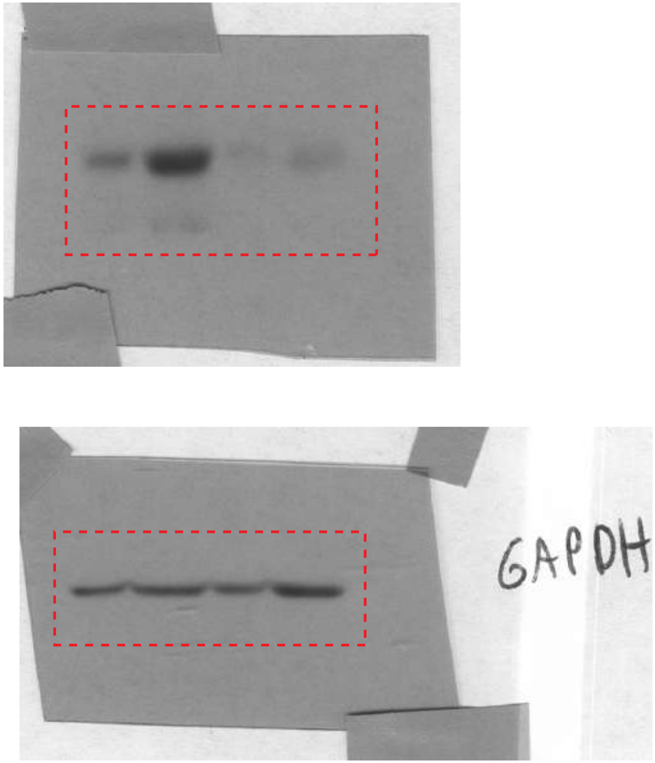

RCOR1

GAPDH

FIGURE S6C - RCOR1 overexpression and RPB1 phosphorylation

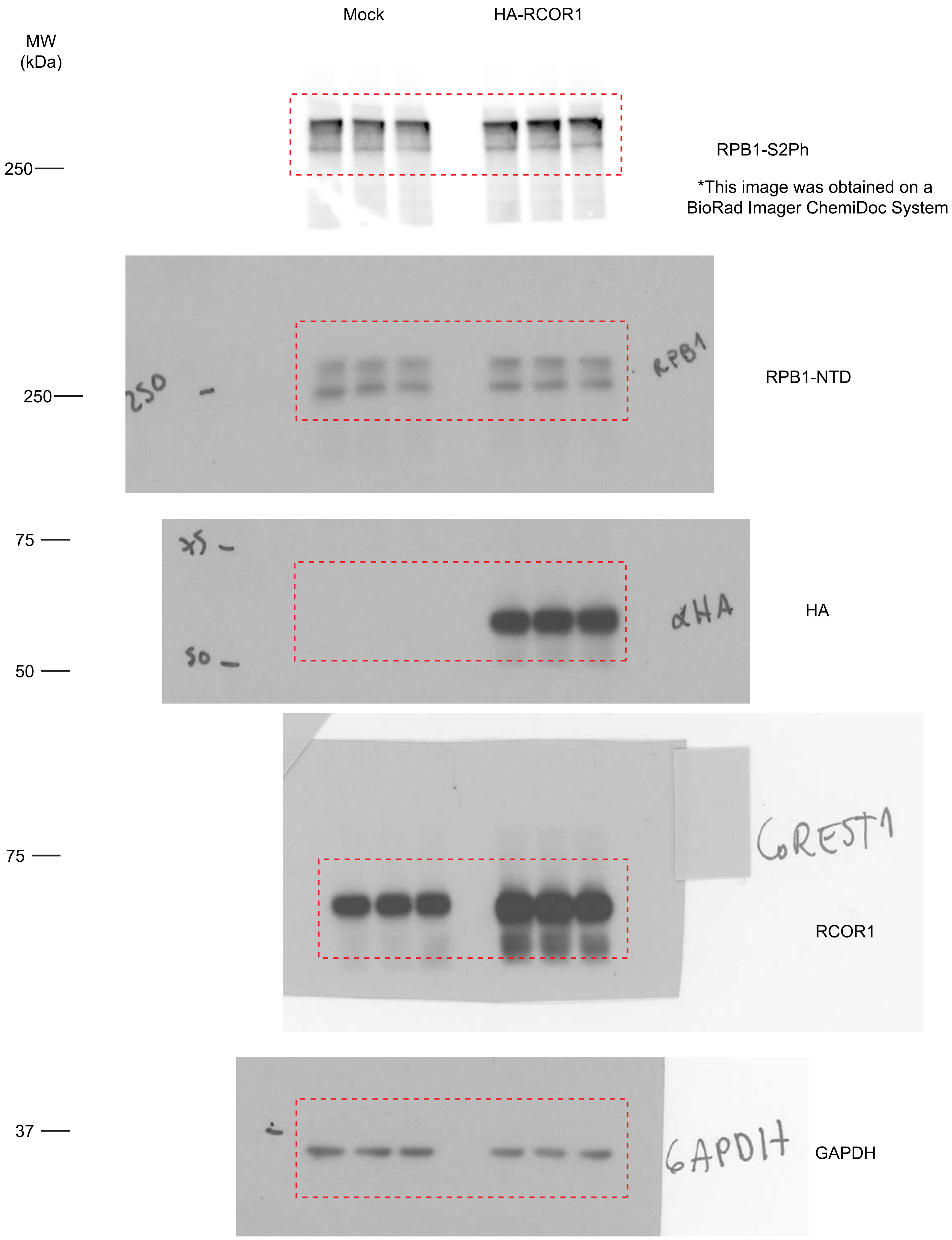

FIGURE S6D - RCOR1 knockdown and RPB1 Phosphorylation

MW (kDa)

siControl siRCOR1

250

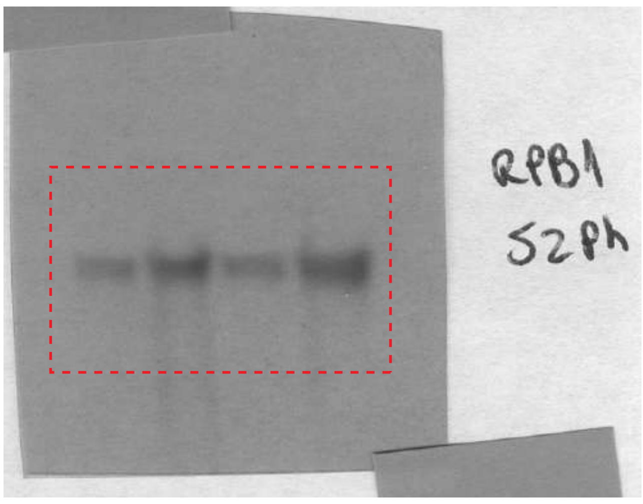

RPB1-S2Ph

250

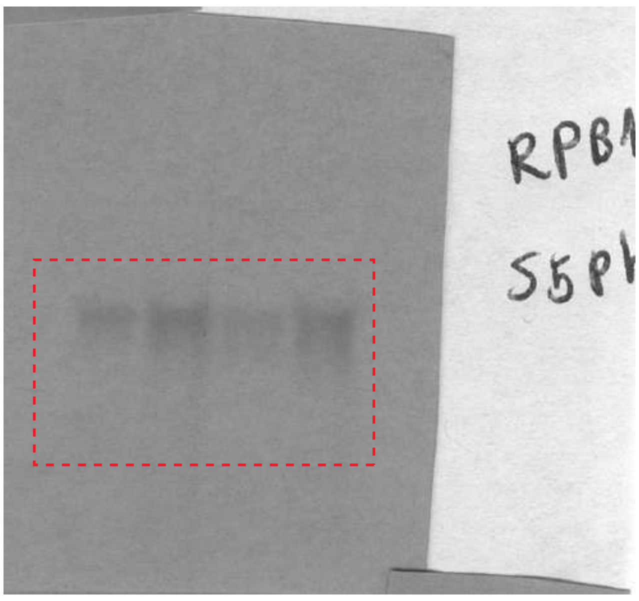

250

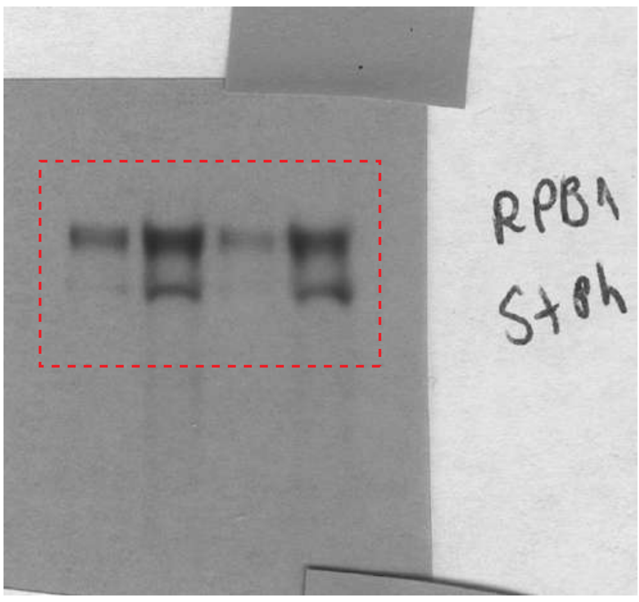

250

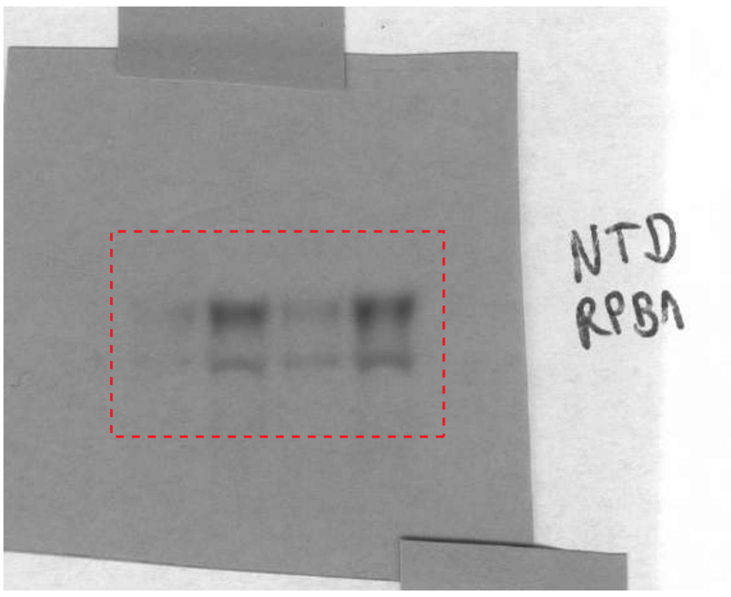

RPB1-NTD
