# Supplementary material for: Unveiling RCOR1 as a rheostat at transcriptionally permissive chromatin
Source: Nat Commun. 2022 Mar 23;13:1550. doi: 10.1038/s41467-022-29261-0 (PMC8943175; doi:10.1038/s41467-022-29261-0)
Supplement: Supplementary file 2 — Peer review file [file 41467_2022_29261_MOESM2_ESM.pdf]

nature portfolio

Peer Review File

Unveiling RCOR1 as a rheostat at transcriptionally permissive chromatin

Open Access This file is licensed under a Creative Commons Attribution 4.0 International License, which permits use, sharing, adaptation, distribution and reproduction in any medium or format, as long as you give appropriate credit to the original author(s) and the source, provide a link to the Creative Commons license, and indicate if changes were made. In the cases where the authors are anonymous, such as is the case for the reports of anonymous peer reviewers, author attribution should be to 'Anonymous Referee' followed by a clear attribution to the source work. The images or other third party material in this file are included in the article's Creative Commons license, unless indicated otherwise in a credit line to the material. If material is not included in the article's Creative Commons license and your intended use is not permitted by statutory regulation or exceeds the permitted use, you will need to obtain permission directly from the copyright holder. To view a copy of this license, visit http://creativecommons.org/licenses/by/4.0/.

REVIEWER COMMENTS</B>

Reviewer #1 (Remarks to the Author):

This manuscript investigates mechanistic features of the LSD1/HDAC1/CoREST complex and its role in regulating gene expression. The first part of the manuscript uses cell immunofluorescence imaging and cell and nuclear fractionation (including micrococcal nuclease) studies to analyze the location of the CoREST (RCOR1) complex. The authors find that there is a strong correlation of the CoREST complex subunits associated with various nuclear/chromatin fractions. The authors then show, unexpectedly, that the CoREST complex preferentially associates with active histone marks like H3K4me3 and H3K18ac rather than heterochromatin marks like H3K27me3. Comparison with PRC2 component EZH2 displayed a different pattern in this regard. They use a computational method based on HiC data related to chromosome 20 in K562 cells to analyze the network of associations of histone marks and CoREST complex components. In addition, existing ENCODE ChIP-Seq data from K562s was analyzed to localize CoREST complex subunits within chromatin globally. Their model based on these simulations and experiments is consistent with the concept that the CoREST complexes is primarily in a position to participate in functions at actively transcribed genes (both at promoters and in gene bodies). To understand what was driving this association, since it was impossible to understand based on the conventional idea of a particular transcription factor like REST or SNAIL being responsible, the authors performed coIP experiments with RCOR1. They identified RNA Pol II, specifically the catalytic subunit RBP1, as associating with RCOR1. They found that CTD phosphorylated RBP1 was especially associating with RCOR1 in this regard. Furthermore, a series of inhibitors that can block Cdk9 (flavopiridol), Cdk7 (THZ1), as well as actinomycin D and cordycepin were used to parse the timing of RCOR1/RBP1 (and its phospho-state) association in the stages of transcription initiation, proximal pausing, and elongation in HT22 cells. THZ1 blocked association whereas actinomycin D and cordycepin enhanced it, so it was deduced that RCOR1 is recruited to Pol II at initiation. To look at the potential functional significance of this RCOR1/RBP1 association, the authors analyzed transcription using EU labeling in HT22 cells and employed corin, entinostat, and tranylcypromine (TCP) as pharmacological probes of the CoREST complex, class I HDACs, and LSD1, respectively. The upshot was that both corin and entinostat enhanced transcription whereas TCP did not. It was concluded that it was the HDAC rather than the LSD1 activity of the CoREST complex that was most important. The authors hypothesized that perhaps this HDAC activity of the CoREST complex could be deacetylating RBP1 itself as part of its repressive activity. The authors observed enhanced RBP1 acetylation (but not methylation) with corin treatment. Overall, I found this to be a fascinating read and was intrigued by the unorthodox mechanistic ideas about how the CoREST complex may be working. If the authors are correct about their model that the CoREST complex regulates transcription in a transcription factor independent way in the fashion delineated here, this would be a ground-breaking discovery. I imagine that there will be some (perhaps most) members of the molecular biology community who will be so skeptical of this idea that they will demand a long list of additional experiments for the authors to "prove" this model beyond doubt. I do not wish to join this legion of critics. I feel like the authors have done a lot of work here at the biochemical and cellular level to make a reasonable case that their idea should be considered carefully. Obvious questions that are relevant and not yet answered are what is the molecular basis of RBP1/RCOR1 interaction (if it is indeed direct) and how is it regulated? How important is CTD acetylation and how does it contribute? Follow-up studies will certainly be needed but given what has been done and the newness of the idea, I don't want to impede the release of this manuscript. Below, I do have a set of what I would consider minor (and hopefully constructive) requests to strengthen the current findings.

1. The reliance on THZ1, a covalent Cdk7 inhibitor is not ideal as this compound can block

other kinases, and Cdk7 itself phosphorylates many things. I would suggest repeating some of the key experiments with triptolide which blocks the TFIIH XPB subunit and has been used by Lis and colleagues to investigate the role of initiation.

2. The authors state that TCP did not affect transcription (Fig 8D) leading them to conclude that the demethylase activity of the CoREST complex is dispensable in the rheostat model here. To my eye, there was a bump in transcript signal with TCP, even if it did not achieve statistical significance. It might be advisable to use a more specific LSD1 inhibitor like GSK-LSD1 to clarify this point. TCP is a more potent monoamine oxidase inhibitor than an LSD1 inhibitor and perhaps its off targets are a problem.

3. The CTD acetylation depicted in Fig 8 could be more convincing. Perhaps the authors could get a hold of the CTD K7ac antibody described by Melanie Ott's lab (PMID: 31054975) and blot with it?

4. Ott and coworkers have suggested that CTD acetylation is associated with stress response/MAP kinase related gene activation. Could the authors mine existing data, or if they feel it it is worth it, use corin (or RCOR1 knockdown) in the context of EGF to determine if stress genes are especially modulated by CoREST inhibition?

5. It would be nice to mine ChIP-Seq data beyond RCOR1 for other HDAC corepressors like Sin3 or NuRD to see if the RCOR1 localization in chromatin is special and distinct. Perhaps there could be a more general (and unappreciated) role of corepressor complexes with rheostat functions?

Reviewer #2 (Remarks to the Author):

The manuscript by Rivera et al shows that the transcriptional repressor RCOR1 is found mainly in open chromatin on active genes where it interacts with Pol II on active promoters and, specifically, can interact with paused Pol II. Perturbation of RCOR1 levels leads to changes in immediate transcription consistent with a previously defined role of RCOR1 as a repressor, but given the new data, suggesting that RCOR1 is a fine-tune regulator of active transcription rather than a classic repressor. The paper is interesting overall. The main point shown in the paper is that RCOR1 preferentially interacts with euchromatin, but the message that authors wish to impart is that RCOR1 functions as a regulator of transcription by throttling Pol II output at promoters. The overall message is supported by mostly excellent Figure 7 but the message is abbreviated in essentially one figure.

Major points.
Line 140-148. Separation of RCOR1 and EZH2 is proposed based on 1D. Co-localization o LSD1, RCOR1 and HDAC1/2 is observed in 1E. Should the samples shown in 1E be probed with EZH2? This is an essential control for separation with EZH2 if authors wish to make such a statement.
Figure 2B and Figure 6B. A negative control factor or histone modification western is missing.
Figure 4. It is unclear what this figure that analyzes public data adds to this manuscript. That RCOR1 signal is enriched at promoters is not by itself surprising as many point-source proteins are or appear to be enriched at promoters. It is more impressive when ChIP-seq signal is not enriched at promoters such as the case for CTCF, for example. Secondly, the two repressive histone marks are actually enriched outside of promoters, so showing them in a promoter-centric manner might not be useful. Third, what is the rationale for choosing 4 clusters?

The manuscript has quite a few interesting observations that convincingly show euchromatin localization of RCOR1. However, much of the impact of the work is not in localization, but in showing RCOR1 as a rapid-acting rheostat. This very message hinges on Figure 7H and specifically on one 20-minute datapoint showing faster release from FP blockage in the presence of lower levels of RCOR1. This is very significant and impactful, but is one effective datapoint sufficient to drive this message?
What do authors mean by transcription speed? Is it frequency of initiation, frequency or release from pausing, or elongation velocity?
Minor point
Line 41. "Thus" as a connection between these two sentences does not make sense. How does the ability to define chromatin landscapes by only two sets of marks follow from both types of chromatin requiring the activity of chromatin modifiers?
45-46. "These specific chromatin signatures …". This sentence is too simplistic and misleading. There is no reason to believe that these marks act on Pol II recruitment directly as it is stated.
51-52. When referring to comparison of RCOR1 member activity, was it in vitro? If so, this needs to be specified.
58. Should be "stimulates"
61. Should this be "includes" instead of "encloses"?
66-67. Are authors implying that RCOR-LSD1 complex can interact with DNA, not just nucleosomes, in the absence of additional DNA-binding transcription factor(s)?
81. "Suggesting RCOR1" should be "suggesting that RCOR1".
93-96. This sentence does not make sense. The first part is too obvious to draw specific conclusions. Secondly, how collisions with chromatin - which are only presumed anyway based on tracking of DNA binding proteins - would contribute to establishing the equilibrium. Maybe simplify the sentence by saying simply "we hypothesized that….".
98. The text mentions low amounts of the neuronal variant, but the figure S1A shows relative levels.
Figure 1F. A panel accompanying this figure to show the extent of digestion and fragment size distribution should be shown.
103. Can authors comment on what fraction of RCOR1 is found in proximity to HDACs? In the absence of a negative colocalization protein control, which might be hard to arrange, it is hard to determine to what extent these are found together.
Figure 1A. RCOR1 green signal is present in the leftmost panel along with blue DNA so it is hard to say that it is excluded. Is this an artefact of this particular panel?
Figure 1C. GAPHD is present in the nucleus and nuclear pellet. In personal experience this is one of the better markers of cytoplasm – can authors comment about possible cytosol contamination of the nuclear prep? This does not seem to be the case in 1D.
119-121. Clarify the terminology between chromatin and chromatin-insoluble fractions.
181. Delete "it".
182-183. Looking at Figure 2D, it appears that RCOR co-fractionates with EZH2 in normal salt, but might be dislodged from a complex with EZH2 with salt as shown in 1D. This interpretation appears to be alternative what authors propose here. In other words, I do not see compelling evidence for separation of EZH2 from RCOR1 at physiological conditions. Can authors comment?
Figure 2E. The H3K27Me3 signal appears to be sparce as it should be, so it may be hard to ascertain absence of contacts with K27Me3.
Figure 3D, E. What is the significance of showing both of these panels in the main figure?
209. It appears to me that by this point the wording "paradoxical" should be toned down or deleted. This section does not resolve this paradox anyway.
Figure 4B. Either colors should be changed for legibility or text labels should be added.
223-224. Why single out bidirectional promoters here? This transition is too abrupt and does not necessarily fit the flow of the manuscript. If authors wish to keep this for results and not discussion, this may need to be explained and a citation added.

Figure 4D. Can cluster I be dominated by shorter genes or very highly expressed genes? Figure 5 mentions expression, but could it be that these are short genes? The accompanying supplemental Figure S4 should have scale bars.
285-286. Can a conclusion that the binding of RCOR1 to Pol II involves its associated enzymes be made here? These can still be in theory completely independent events. In other words, the interactions detected here can be in principle coincidental, not functional.
310-315. Can author point to the figure panel/lane or explain where they find enrichment in RCOR1-RPB1 complexes. I am unable to see this. To me the lines with DMSO, Flavopiridol, Cordycepin, and Act D look the same.
Figure 7 C, F, H. Is there a way to show wider-field zoomed out microscopic images including more cells perhaps as a supplement?
Figure 7 L and K. Given a smear in 7L that is being cut what seems like in the middle, the entire gel should be shown at least in the supplement.

Point by point answer to reviewers:

Reviewer #1 (Remarks to the Author):

This manuscript investigates mechanistic features of the LSD1/HDAC1/CoREST complex and its role in regulating gene expression. The first part of the manuscript uses cell immunofluorescence imaging and cell and nuclear fractionation (including micrococcal nuclease) studies to analyze the location of the CoREST (RCOR1) complex. The authors find that there is a strong correlation of the CoREST complex subunits associated with various nuclear/chromatin fractions. The authors then show, unexpectedly, that the CoREST complex preferentially associates with active histone marks like H3K4me3 and H3K18ac rather than heterochromatin marks like H3K27me3. Comparison with PRC2 component EZH2 displayed a different pattern in this regard. They use a computational method based on HiC data related to chromosome 20 in K562 cells to analyze the network of associations of histone marks and CoREST complex components. In addition, existing ENCODE ChIP-Seq data from K562s was analyzed to localize CoREST complex subunits within chromatin globally. Their model based on these simulations and experiments is consistent with the concept that the CoREST complexes is primarily in a position to participate in functions at actively transcribed genes (both at promoters and in gene bodies). To understand what was driving this association, since it was impossible to understand based on the conventional idea of a particular transcription factor like REST or SNAIL being responsible, the authors performed coIP experiments with RCOR1. They identified RNA Pol II, specifically the catalytic subunit RBP1, as associating with RCOR1. They found that CTD phosphorylated RBP1 was especially associating with RCOR1 in this regard. Furthermore, a series of inhibitors that can block Cdk9 (flavopiridol), Cdk7 (THZ1), as well as actinomycin D and cordycepin were used to parse the timing of RCOR1/RBP1 (and its phospho-state) association in the stages of transcription initiation, proximal pausing, and elongation in HT22 cells. THZ1 blocked association whereas actinomycin D and cordycepin enhanced it, so it was deduced that RCOR1 is recruited to Pol II at initiation. To look at the potential functional significance of this RCOR1/RBP1 association, the authors analyzed transcription using EU labeling in HT22 cells and employed corin, entinostat, and tranylcypromine (TCP) as pharmacological probes of the CoREST complex, class I HDACs, and LSD1, respectively. The upshot was that both corin and entinostat enhanced transcription whereas TCP did not. It was concluded that it was the HDAC rather than the LSD1 activity of the CoREST complex that was most important. The authors hypothesized that perhaps this HDAC activity of the CoREST complex could be deacetylating RBP1 itself as part of its repressive activity. The authors observed enhanced RBP1 acetylation (but not methylation) with corin treatment.

Overall, I found this to be a fascinating read and was intrigued by the unorthodox mechanistic ideas about how the CoREST complex may be working. If the authors are correct about their model that the CoREST complex regulates transcription in a transcription factor independent way in the fashion delineated here, this would be a ground-breaking discovery. I imagine that there will be some (perhaps most)

members of the molecular biology community who will be so skeptical of this idea that they will demand a long list of additional experiments for the authors to "prove" this model beyond doubt. I do not wish to join this legion of critics. I feel like the authors have done a lot of work here at the biochemical and cellular level to make a reasonable case that their idea should be considered carefully. Obvious questions that are relevant and not yet answered are what is the molecular basis of RBP1/RCOR1 interaction (if it is indeed direct) and how is it regulated? How important is CTD acetylation and how does it contribute? Follow-up studies will certainly be needed but given what has been done and the newness of the idea, I don't want to impede the release of this manuscript. Below, I do have a set of what I would consider minor (and hopefully constructive) requests to strengthen the current findings.

We thank the reviewer for this positive feedback and constructive insights about our present work. We have addressed the relatively minor concerns in the revised manuscript.

1. The reliance on THZ1, a covalent Cdk7 inhibitor is not ideal as this compound can block other kinases, and Cdk7 itself phosphorylates many things. I would suggest repeating some of the key experiments with triptolide which blocks the TFIIH XPB subunit and has been used by Lis and colleagues to investigate the role of initiation.

We performed the experiments with triptolide as suggested. Here, we show by immunostainings that low doses (100 nM) and short treatments (30 min) of triptolide down-regulated RNA Pol II levels, whereas THZ1 did not (Figure S5F). Triptolide has already been reported to induce RNA Pol II degradation (10.1371/journal.pone.0023993). Therefore, we consider that the use of triptolide would have led to a misinterpretation of the results.

Since there are no other bona-fide inhibitors for RNA Pol II transcription initiation, we decided to strengthen the evidence for the interaction of RCOR1 with RNA Pol II by performing a proximity ligation assay (Fig. 5G). This assay allowed us to visualize the spots where both RCOR1 and RPB1 are colocalizing, and by counting the number of colocalizing spots per cell we found a significant colocalization of RCOR1 with RPB1I, which significantly decreased upon THZ1 treatment and increased with actinomycin D, reinforcing the evidence that RCOR1 is recruited after transcription initiation. In addition, we would like to provide replicates of immunoprecipitation experiments showing that THZ1 decreases the co-precipitation of RPB1 under RCOR1 IPs (Figure S5C).

2. The authors state that TCP did not affect transcription (Fig 8D) leading them to conclude that the demethylase activity of the CoREST complex is dispensable in the rheostat model here. To my eye, there was a bump in transcript signal with

TCP, even if it did not achieve statistical significance. It might be advisable to use a more specific LSD1 inhibitor like GSK-LSD1 to clarify this point. TCP is a more potent monoamine oxidase inhibitor than an LSD1 inhibitor and perhaps its off targets are a problem.

We thank the referee for the suggestion. We agree TCP was not the best choice for inhibiting LSD1. We decided to test GSK-LSD1 as a more specific way to inhibit LSD1 in order to test if this enzyme is responsible for the global repression caused by RCOR1 overexpression. Intriguingly, we found that it is needed and LSD1 inhibition by GSK-LSD1 can partially rescue the RCOR1 overexpression phenotype. New Figure 6D shows the new set of experiments. With this data, we conclude that both enzyme activities associated with RCOR1 are involved in regulating active transcription.

3. The CTD acetylation depicted in Fig 8 could be more convincing. Perhaps the authors could get a hold of the CTD K7ac antibody described by Melanie Ott's lab (PMID: 31054975) and blot with it?

We acknowledge the referee for this suggestion. After contacting Dr. Ott, she kindly shared the antibody that allowed us to carry out these experiments. Using this valuable tool, we conducted additional experiments to show that RCOR1 regulates the deacetylation of RPB1-K7. First, we compared the levels of RPB1-K7ac in two biological replicates of HT22 cells treated with Corin or DMSO, finding that Corin induced an accumulation of RPB1-K7ac (Figure 7H). Then, we show that this accumulation occurs in three subcellular compartments where RPB1 is detected (cytosol, nuclear soluble, and chromatin fractions) (Figure 7 I). Both approaches showed that Corin increases the acetylation levels of RPB1-K7ac, indicating that RPB1 is an RCOR1-HDAC target for deacetylation of its CTD non-canonical lysine residues, positioning RCOR1 as a regulator of this novel modification associated with both promoter-proximal pausing and elongation stages of mammalian transcription (10.1016/j.molcel.2013.10.009).

4. Ott and coworkers have suggested that CTD acetylation is associated with stress response/MAP kinase related gene activation. Could the authors mine existing data, or if they feel it it is worth it, use corin (or RCOR1 knockdown) in the context of EGF to determine if stress genes are especially modulated by CoREST inhibition?

As suggested by the reviewer, we tested the involvement of the RCOR1 complex in the regulation of EGF pathway genes. As shown in figure 7J, we successfully found the EGF receptor present in HT22 cells, allowing us to study the role of the RCOR1 complex in the stimulation of RPB1-K7ac dependent genes. We report that Corin significantly enhanced EGF-induced *cFos* and *egr2* transcription, underscoring that the complex is regulating genes that are induced in a RPB1-K7ac dependent way.

5. It would be nice to mine ChIP-Seq data beyond RCOR1 for other HDAC corepressors like Sin3 or NuRD to see if the RCOR1 localization in chromatin is

special and distinct. Perhaps there could be a more general (and unappreciated) role of corepressor complexes with rheostat functions?

We analyzed the overlap between RCOR1, SIN3A, and CHD4 (NurD subunit). The data showed a significantly higher number of RCOR1 peaks compared to Sin3A and CHD4 indicating that RCOR1 targets a broader area of the genome (Figure S3E). While the 81% of RCOR1 peaks did not overlap the other co-repressors, we detected a strong overlap between Sin3A (94.5%) and CHD4 (77.7%) with RCOR1, suggesting that more than one HDAC-containing corepressor complex is often involved in the regulation of subsets of the RCOR1 target regions.

Reviewer #2 (Remarks to the Author):

The manuscript by Rivera et al shows that the transcriptional repressor RCOR1 is found mainly in open chromatin on active genes where it interacts with Pol II on active promoters and, specifically, can interact with paused Pol II. Perturbation of RCOR1 levels leads to changes in immediate transcription consistent with a previously defined role of RCOR1 as a repressor, but given the new data, suggesting that RCOR1 is a fine-tune regulator of active transcription rather than a classic repressor. The paper is interesting overall. The main point shown in the paper is that RCOR1 preferentially interacts with euchromatin, but the message that authors wish to impart is that RCOR1 functions as a regulator of transcription by throttling Pol II output at promoters. The overall message is supported by mostly excellent Figure 7 but the message is abbreviated in essentially one figure.

We thank Reviewer #2 for the constructive feedback. Regarding the concern about showing the most impactful message in a single figure, we would like to mention that, without the fundamental and exploratory research performed in the first 4 main figures, we would not have been able to follow a direction towards unveiling a non-conventional role of RCOR1 at active regions. In response to the specific concern, however, we have included many new experiments, as highlighted below and summarized in the letter to the editor. In the revised manuscript, our key new findings are now presented in Figures 6 and 7.

Major points.

Line 140-148. Separation of RCOR1 and EZH2 is proposed based on 1D. Co-localization o LSD1, RCOR1 and HDAC1/2 is observed in 1E. Should the samples shown in 1E be probed with EZH2? This is an essential control for separation with EZH2 if authors wish to make such a statement.

We thank the reviewer for requesting this important control. As shown in the revised new figure 1F, RCOR1 immunoprecipitation that efficiently precipitates LSD1 shows no interaction with EZH2, further strengthening the evidence that RCOR1 complexes do not share chromatin territories with EZH2 complexes.

Figure 2B and Figure 6B. A negative control factor or histone modification western is missing.

We now include a histone modification (H3R2me2a) as a negative control in new figure 2C. Coimmunoprecipitation assays with RCOR1 show that specifically precipitates RPB1 in its different phosphorylated forms, while it is unable to precipitate the equivalent catalytic subunits of RNA Pol I and RNA Pol III (Fig. 5A).

Figure 4. It is unclear what this figure that analyzes public data adds to this manuscript. That RCOR1 signal is enriched at promoters is not by itself surprising as many point-source proteins are or appear to be enriched at promoters. It is more impressive when ChIP-seq signal is not enriched at promoters such as the case for CTCF, for example. Secondly, the two repressive histone marks are

actually enriched outside of promoters, so showing them in a promoter-centric manner might not be useful. Third, what is the rationale for choosing 4 clusters?

Our main purpose for the promoter-centric metagene analysis was to test whether RCOR1 binds to euchromatin regions. Unlike transcriptional repressors like PRC2 that are enriched in H3K27me3 enriched repressed genes, our data show RCOR1 is mainly found upstream of actively transcribing genes, near TSS. We agree that promoter-centric analysis does not cover genome-wide information. Thus, we decided to run a non-gene-centric approach by analyzing the correlation (Spearman) between RCOR1 and both active and repressive histone marks by 10kb bin regardless of genic or intergenic regions. These new analyses show that RCOR1 depicts a higher correlation with active histone marks than with repressive ones (please see new figure 3C). We also provide a peak-centered meta-analysis of the correlation between RCOR1 and H3K27ac or H3K27me3 (Figure S3D) showing a positive correlation of RCOR1 with H3K27ac but not with H3K27me3, in line with our existing findings. We thank this reviewer for this criticism, now we can confidently claim that RCOR1 is associated with active histone modification at the genome-wide level.

Regarding the rationale for the number of clusters selection, 4 clusters were the result of optimization for extracting essential features of RCOR1 and other factors binding and histone marks - decreasing the number loses the cluster-specific features (e.g. cluster 1 vs 2 - K4me3 peaks after vs before TSS will be lost if we choose k=3) and increasing k causes redundant clusters.

We included an explanation for the clustering approach in the current version of the manuscript (lines 228-234). Their individual detailed features are:

•Cluster 1: strong H3K4me3 peaks at DOWNstream of TSS and RCOR1 – canonical H3K4me3 pattern.
•Cluster 2: stronger H3K4me3 peak at UPstream of TSS - implying RCOR1 might have a specific role in bidirectional genes of divergent genes.
•Cluster 3: weak RCOR1 binding with weaker H3K4me3 and other active histone marks - showing RCOR1 primarily binds to highly expressing genes.
•Cluster 4: no RCOR1 binding and relatively higher enrichment of H3K27me3.

The manuscript has quite a few interesting observations that convincingly show euchromatin localization of RCOR1. However, much of the impact of the work is not in localization, but in showing RCOR1 as a rapid-acting rheostat. This very message hinges on Figure 7H and specifically on one 20-minute datapoint showing faster release from FP blockage in the presence of lower levels of RCOR1. This is very significant and impactful, but is one effective datapoint sufficient to drive this message?

We thank Reviewer #2 for appreciating the intriguing observations and for the constructive input. The point of concern prompted us to reinforce the evidence for

the role of RCOR1 as a rheostat that regulates the transcription of active genes. We now include three other experimental approaches.

(1) We performed an in vitro transcription assay using chromatin-free nuclear extracts obtained from HeLa cells treated with DMSO or Corin. Time courses were sampled using equal amounts of template and extracts and the transcription had a higher yield when using Corin-derived extracts, reaching a statistical significant difference at 90 minutes of assay (Figure 6G).

(2) We are also highlighting in this current version the effect of short-term inhibition of the RCOR1 complex on transcription by incubating cells for 4 hours with Corin. Imaging of nascent RNA showed a significant increase in transcription in Corin-treated cells (Figure 7A-D), similarly to our data obtained when knocking RCOR1 down. This effect was accompanied by an increase of active histone marks that are targets of the RCOR1 complex (Fig. 7B).

(3) We have also tested the effect of inhibiting the RCOR1 complex on the expression of some specific genes of the EGF pathway. As shown in Figure 7J, Corin enhanced the EGF-induced upregulation of *cFos* and *egr2* mRNA expression, which depend on EGF in a RPB1-K7ac way, but not *eif4a1,* which does not. Although this is an end-point experiment, these EGF targets are immediate early genes (IEGs), which are rapid and transiently induced, suggesting that RCOR1 regulates that process. In fact, the treatment of Corin was sufficient to induce upregulation of their mRNA levels, suggesting that the RCOR1 complex is downstream of their EGF activation pathway. Last, but not least, we would like to highlight that the current version of our manuscript strengthened the data that reveal RCOR1 as a regulator of RNA Pol II CTD acetylation, which we found to be specifically targeted towards the non-canonical residue lysine 7, present in 8 out of 52 heptapeptide repeats on mammalian RPB1. Interestingly, RPB1-K7ac is enriched in promoter-proximal pausing regions as well as at elongation steps of transcription, similar to the times where we found RCOR1 associating with the transcriptional machinery. Our findings underscore the RCOR1 complex as an eraser of this modification and also as a downstream acting factor of the EGF-activation mechanism of genes that respond to EGF in a RPB1-K7 acetylation dependent manner.

Altogether, these new data plus Flavopiridol release experimental data strongly support our conclusion that the RCOR1 complex is a rheostat that regulates the transcription of genes at actively expressed chromatin.

What do authors mean by transcription speed? Is it frequency of initiation, frequency or release from pausing, or elongation velocity?

Because we do not have actual kinetic data, we decided to avoid the use of "transcription speed". Rather we now use transcriptional activity or yield.

Minor point

Line 41. “Thus” as a connection between these two sentences does not make sense. How does the ability to define chromatin landscapes by only two sets of marks follow from both types of chromatin requiring the activity of chromatin modifiers?

We rephrased the section in order to provide a clearer statement regarding the need for chromatin modifiers to partially establish or maintain features of chromatin domains.

45-46. “These specific chromatin signatures …”. This sentence is too simplistic and misleading. There is no reason to believe that these marks act on Pol II recruitment directly as it is stated.

We rephrase that paragraph in the Introduction section (lines 34-42)

51-52. When referring to comparison of RCOR1 member activity, was it in vitro? If so, this needs to be specified.

Corrected (line 47)

58. Should be “stimulates”

Corrected (line 54)

61. Should this be ”includes” instead of “encloses”?

Corrected (line 56).

66-67. Are authors implying that RCOR-LSD1 complex can interact with DNA, not just nucleosomes, in the absence of additional DNA-binding transcription factor(s)?

As we mentioned in the text, it has been shown that the RCOR1-LSD1 complex directly interacts with nucleosomal DNA. Regarding its eventual interaction with nucleosome-free DNA, in vitro experiments have been reported showing a submicromolar interaction between them (DOI: 10.1073/pnas.1419468112). Also, Kim et al (DOI: 10.1016/j.molcel.2020.04.019) recently showed that RCOR1 binds directly to both histones and DNA components of the nucleosome.

81. “Suggesting RCOR1” should be “suggesting that RCOR1”.

Corrected (line 61)

93-96. This sentence does not make sense. The first part is too obvious to draw specific conclusions. Secondly, how collisions with chromatin - which are only presumed anyway based on tracking of DNA binding proteins - would contribute to establishing the equilibrium. Maybe simplify the sentence by saying simply “we hypothesized that….”.

We agree with the reviewer and have rephrased it to be clearer (lines 91-92).

98. The text mentions low amounts of the neuronal variant, but the figure S1A shows relative levels.

Corrected (line 94)

Figure 1F. A panel accompanying this figure to show the extent of digestion and fragment size distribution should be shown.

We now show in Figure S2A that before extracting MNase-treated chromatin fragments, mostly all the products are found in the nuclear pellet, but when extracting them with our protocol, we were able to solubilize the majority of digested chromatin, ranging from 1 to >6 nucleosome units.

103. Can authors comment on what fraction of RCOR1 is found in proximity to HDACs? In the absence of a negative colocalization protein control, which might be hard to arrange, it is hard to determine to what extent these are found together.

We are aware that the colocalization between HDAC1/2 and RCOR1 is not complete. This is not surprising since (1) HDAC1/2 proteins are known to be part of multiple co-repressor complexes such as NuRD, Sin3A, MiDAC, RCOR, among others (DOI:10.1038/msb.2013.26) and (2) the composition of the RCOR1 complex is dynamic (DOI: 10.1111/jnc.12457). That is why we decided to quantify the degree of 2D-spatial colocalization between them, as shown in Figure 1B. The Vann Steensel´s approach shows that the positions of the fluorescent signals coming from RCOR1 and its associated enzymes are non-stochastically and positively correlated. In addition, our negative control turned out to be DNA dense regions, where we observed an exclusion instead of a peak of correlation.

Figure 1A. RCOR1 green signal is present in the leftmost panel along with blue DNA so it is hard to say that it is excluded. Is this an artefact of this particular panel?

We are aware that although some of the RCOR1 spots are located to these pericentric heterochromatin clusters, the majority of RCOR1 is located outside these territories as quantitated by the Van Steensel’s test (Figure 1 B). Thus, we decided to rephrase the message from “excluded” to “mostly excluded”.

We also show a group of HT22 cells exhibiting a similar pattern (Figures S1B S1C):

Figure 1C. GAPHD is present in the nucleus and nuclear pellet. In personal experience this is one of the better markers of cytoplasm – can authors comment about possible cytosol contamination of the nuclear prep? This does not seem to be the case in 1D.

We now include two additional cytosolic markers analyzed in the same subset of fractions. MEK1/2 and Tubulin exhibited a better cytosolic enrichment than GAPDH.

119-121. Clarify the terminology between chromatin and chromatin-insoluble fractions.

We rephrase the paragraph (lines 111-115).

181. Delete “it”.

Corrected.

182-183. Looking at Figure 2D, it appears that RCOR co-fractionates with EZH2 in normal salt, but might be dislodged from a complex with EZH2 with salt as shown in 1D. This interpretation appears to be alternative what authors propose here. In other words, I do not see compelling evidence for separation of EZH2 from RCOR1 at physiological conditions. Can authors comment?

As shown in new Figure 1E, EZH2 is not detected on RCOR1 immunocomplexes. In addition, as shown in Figures S3F and S3G, ChIP seq data correlations between RCOR1 and EZH2 showed some degree of overlapping at genome-wide scales but not when looking specifically at gene-centered RCOR1 signals. Suggesting that in active genes, RCOR1 and EZH2 are not colocalizing.

Figure 2E. The H3K27Me3 signal appears to be sparce as it should be, so it may be hard to ascertain absence of contacts with K27Me3.

We agree on this point. To make sure about our observations we quantitated the correlation between H3K27me3 and RCOR1, showing a positive but less significant proximity between them. In addition, we are now providing a genome wide spearman correlation and peak-centered global meta-analysis of RCOR1 and different ChIP-seq peaks of histone modifications including H3K27me3 (Fig 3C and S3D). Both approaches support our microscopy findings.

Figure 3D, E. What is the significance of showing both of these panels in the main figure?

We initially decided to put both images as a way to provide a visualization of RCOR1 and H3K4me3 with and without DNA. In this new version, we decided to only keep ex Figures 3C, 3D and 3F as new Figures 2H and 2I, moving the rest of ex Figure 3 to Figures S2C-E.

209. It appears to me that by this point the wording “paradoxical” should be toned down or deleted. This section does not resolve this paradox anyway.

We deleted the word “paradoxical”.

Figure 4B. Either colors should be changed for legibility or text labels should be added.

We thank the reviewer for this suggestion, in this new version we used different colors for each genomic region (New Figure S3A-B) that follow the same color code as the enrichment analyses (Figure 3B).

223-224. Why single out bidirectional promoters here? This transition is too abrupt and does not necessarily fit the flow of the manuscript. If authors wish to keep this for results and not discussion, this may need to be explained and a citation added.

We believe that the bidirectional promoter regulation by RCOR1 is an interesting observation. Although pursuing this observation further falls outside the scope of the current manuscript, keeping the finding in the paper will stimulate the field to investigate new directions, particularly in the relationship between RCOR1 and

bidirectional transcription. As suggested by the reviewer, we added two references and improved the transition and flow of the manuscript (lines 250-253).

Figure 4D. Can cluster I be dominated by shorter genes or very highly expressed genes? Figure 5 mentions expression, but could it be that these are short genes? The accompanying supplemental Figure S4 should have scale bars.

It is true that short genes are enriched in cluster 1. However, many genes longer than 10 Kb also show RCOR1 enrichment in the gene body including FAM78A (18kb), VEGFA (16kb), GATA2 (14kb) and others. We added the scale bar in our representative genes depicted in Figure S4.

More importantly, the main difference between cluster 1 and cluster 2 is the K4me3 peak location of the clustered genes - which might reflect the distinct function of RCOR1 in those two clusters. This scope related to bidirectional transcription can be very important, but we thought that this perspective is better to be addressed in a separate study more specifically and thoroughly.

285-286. Can a conclusion that the binding of RCOR1 to Pol II involves its associated enzymes be made here? These can still be in theory completely independent events. In other words, the interactions detected here can be in principle coincidental, not functional.

We agree and have rephrased the statement (lines 298-299).

310-315. Can author point to the figure panel/lane or explain where they find enrichment in RCOR1-RPB1 complexes. I am unable to see this. To me the lines with DMSO, Flavopiridol, Cordycepin, and Act D look the same.

The key finding of this set of experiments was that RCOR1 is getting recruited to RNA Pol II between transcriptional initiation and elongation. We rephrased the paragraph to make it clearer. In addition, to further support our evidence in a quantitative way, we performed proximity ligation assays whose results are now part of Figure 5G and show a significant increase of RCOR1/RPB1 colocalization under Actinomycin D treatments.

Figure 7 C, F, H. Is there a way to show wider-field zoomed out microscopic images including more cells perhaps as a supplement?

Please see the new Figure S7.

Figure 7 L and K. Given a smear in 7L that is being cut what seems like in the middle, the entire gel should be shown at least in the supplement.

Please see the uncropped Western in the new Figure S8.

REVIEWERS' COMMENTS

Reviewer #1 (Remarks to the Author):

The authors have made a comprehensive and very laudable effort to respond to reviewer comments. Their manuscript describes a new model for the role of the CoREST complex in gene regulation and provides a substantial body of data to delineate this function. I fully support publication.

Reviewer #2 (Remarks to the Author):

In the revised manuscript by Rivera, authors addressed concerns and then some. It is very much appreciated. This is now an exciting and well-flowing story. The data are solid. Kudos to the authors. Minor proofreading comments with one comment about a specific Western panel.

Line 30. Associating HDACs should be associating with HDACs.
Line 29, 51, 70. Check whether transcriptionally permissive should have the hyphen or not. Probably not. (Compare with lane 80).
Line 61. Delete "a".
Line 84. Double check if associating HDACs should be associating with HDACs.
Line 135. Should "specific" be either "distinct" or the word deleted altogether? "Specific" would implies functions.
Line 288. Is it really pertinent to state that RPB1 harbors catalytic function? True, much of Pol II active center is technically and structurally made up by RPB1, but by itself the subunit is not functional.
Line 294. The only substantive comment. Figure 5A Western blot panels with Pol I and Pol III largest subunits simply do not look good. It may be the particular image, but one can not tell absence of binding in either panel, for Pol III because of a smear and low input intensity (compare with Rpb1-S2P) or because of high background. Can these two subpanels be removed? They do not appear to add much about the mechanism relevant to this manuscript anyway, and currently the outcomes are not clear.
Line 368. "defficient" is misspelled.

Point by point answer to reviewers:

Reviewer #1 (Remarks to the Author):

The authors have made a comprehensive and very laudable effort to respond to reviewer comments. Their manuscript describes a new model for the role of the CoREST complex in gene regulation and provides a substantial body of data to delineate this function. I fully support publication.

We thank the reviewer again for the positive comments and useful suggestions in reviewing our work.

Reviewer #2 (Remarks to the Author):

In the revised manuscript by Rivera, authors addressed concerns and then some. It is very much appreciated. This is now an exciting and well-flowing story. The data are solid. Kudos to the authors. Minor proofreading comments with one comment about a specific Western panel.

We are very grateful for helping us to improve our work.

Line 30. Associating HDACs should be associating with HDACs.

DONE

Line 29, 51, 70. Check whether transcriptionally permissive should have the hyphen or not. Probably not. (Compare with lane 80).

We have used transcriptionally permissive throughout the manuscript without the hyphen

Line 61.

Delete “a”.

DONE

Line 84. Double check if associating HDACs should be associating with HDACs.

DONE

Line 135. Should “specific” be either “distinct” or the word deleted altogether? “Specific” would implies functions.

DELETED

Line 288. Is it really pertinent to state that RPB1 harbors catalytic function? True, much of Pol II active center is technically and structurally made up by RPB1, but by itself the subunit is not functional.

CORRECTED

Line 294. The only substantive comment. Figure 5A Western blot panels with Pol I and Pol III largest subunits simply do not look good. It may be the particular image, but one can not tell absence of binding in either panel, for Pol III because of a smear and low input intensity (compare with Rpb1-S2P) or because of high background. Can these two subpanels be removed? They do not appear to add much about the mechanism relevant to this manuscript anyway, and currently the outcomes are not clear.

Subpanels corresponding to PolI and PolIII were removed

Line 368. “defficient” is misspelled.

CORRECTED
